# Lumboperitoneal shunt insertion without fluoroscopy guidance: Accuracy of placement in a series of 107 procedures

**DOI:** 10.12688/f1000research.11089.2

**Published:** 2017-06-30

**Authors:** Sabah Al-Rashed, Haider Kareem, Neeraj Kalra, Linda D’Antona, Mouness Obeidat, Bhavesh Patel, Ahmed Toma

**Affiliations:** 1 The National Hospital for Neurology and Neurosurgery, London, WC1N 3BG, UK; 2 Charing Cross Hospital, London, W6 8RF, UK

**Keywords:** Lumboperitoneal shunt, LP shunt, fluoroscopy

## Abstract

***Background**: *Lumboperitoneal (LP) shunts were the mainstay of cerebrospinal fluid diversion therapy for idiopathic intracranial hypertension (IIH). The traditionally cited advantage of LP shunts over ventriculoperitoneal (VP) shunts is the ease of insertion in IIH. This needs to be placed at the level of L3/4 to be below the level of the spinal cord. The objective of this study was to analyse the position of LP shunts inserted without portable fluoroscopy guidance. ***Methods**: *A retrospective analysis of radiology was performed for patients who underwent lumboperitoneal shunts between 2006 and 2016 at the National Hospital for Neurology and Neurosurgery. Patients who had insertion of a LP shunt without fluoroscopy guidance were selected.  Patients without post-procedural imaging were excluded.

A retrospective analysis of the clinical notes was also performed. ***Results**: *Between 2006 and 2016, 163 lumboperitoneal shunts were inserted in 105 patients. A total of 56 cases were excluded due to lack of post-procedural imaging; therefore, 107 post-procedural x-rays were reviewed. In 17 (15.8%) cases the proximal end of the LP shunt was placed at L1/L2 level or above. ***Conclusions**: *Insertion of LP shunts without portable fluoroscopy guidance gives a 15.8% risk of incorrect positioning of the proximal end of the catheter. We suggest that x-ray is recommended to avoid incorrect level placement. Further investigation could be carried out with a control group with fluoroscopy against patients without.

## Introduction

Historically, lumboperitoneal (LP) shunts were the mainstay of cerebrospinal fluid (CSF) diversion therapy for idiopathic intracranial hypertension (IIH). The traditionally cited advantage of LP shunts over ventriculoperitoneal (VP) shunts is the ease of insertion in IIH patients who usually have small and sometimes difficult to catheterise ventricles ^1– 4^.

Multiple studies have shown that when functional, LP shunts are effective in alleviating headaches and improving or stabilising visual symptoms in patients with IIH ^5– 8^. Studies have shown that IIH patients who underwent LP shunting had improvement in both visual acuity and visual fields with patients also reporting an improvement in headache symptoms post LP shunting ^6, 9^. In these previous studies, and in many others, the most common complication was shunt obstruction, with up to 65% of cases requiring revision in one study ^9^. Other less frequent yet significant complications of LP shunts include infection, radiculopathy, shunt migration, syrinx, low pressure headaches, tonsillar herniation, subdural haematomas and potential damage of the distal end of the spinal cord.

The conus medullaris is the tapered, lower end of the spinal cord. Multiple cadaveric studies have demonstrated the level of the conus medullaris to be between T12 and L3 ^10^. Other studies report that the conus reaches the adult level by two years of age and lies at an average position of L1 to L2 ^11^. This position was also confirmed by a large radiological study performed in 1998 ^10^. Due to the proximity of the distal end of the spinal cord, it is best practice to avoid the insertion of LP shunts higher than L2/3 level. The ideal position for this procedure is considered to be at L3/L4 level or below.

The primary advantage of a LP over a VP shunt is the ability to cannulate the CSF space, in this case the thecal sac, as opposed to having to cannulate the very commonly found slit ventricles associated with IIH when considering a VP shunt ^1– 4^. However, there are also a series of challenges associated with this procedure.

Generally LP shunt patients are positioned in the lateral position to provide simultaneous access to the lumbar spine and flank. Percutaneous cannulation of the thecal sac can be very challenging, often requiring specific long needles. Additionally, it is often difficult to get the flexion (“foetal position”) required in these patients to open the interlaminar space and allow for the needle to access the thecal sac. Once the proximal catheter enters the thecal sac it needs to be threaded cranially into position, which is at times challenging as the catheter often kinks within the significant tissue volume. Following placement of the proximal catheter, the remainder then needs to be tunneled through the subcutaneous tissue into the flank region. At this point, while in the lateral position and with the significant amount of adipose tissue, the surgeon then needs to identify the peritoneum. This can prove to be quite challenging given the non-anatomic patient’s position as well as the fact that gravity is working against the surgeon. At this point the catheter is then passed into the flank within the peritoneum.

The insertion of the catheter into the lumbar CSF space determines the success or failure of the LP shunt. Since this involves a manual manoeuvre with a “blind” tap, the catheter may be inadvertently placed incorrectly and migration of the shunt catheters is a common experience. The lumbar catheter can migrate relative to the thecal sac (usually into the subcutaneous space), and the peritoneal catheter can likewise come out of the peritoneum. The incidence ranges from 3–20%. Migration complications have been noted to be more common in the paediatric population ^4, 12, 13^. When a catheter migrates out of the thecal sac, a subcutaneous collection of spinal fluid can be observed.

Newly onset radicular pain has been noted to occur with LP shunts. This may result from catheter migration or localised inflammation leading to arachnoiditis. The onset of symptoms may necessitate shunt revision. The incidence of developing newly onset radicular pain ranges from 5–6% ^13, 14^.

The efficacy of fluoroscopic guidance in the placement of a lumbar catheter in patients treated with an LP shunt has been reported ^9^. The method includes using intraoperative portable fluoroscopy with contrast medium. The direction of the inserted catheter can be confirmed, and loop formation or absence thereof can be detected intraoperatively. It is possible to confirm that the catheter has not migrated into the extra-CSF space or the intervertebral foramen containing the spinal nerve roots. Improved visibility of the catheter in the spine, by filling it with contrast medium, is the key to the success of this procedure ^15^. Intraoperative fluoroscopic guidance has become widely available in last two decades. Despite its efficacy, it exposes the patients and the staff to radiation. Moreover, there are possible side effects and restrictions related to the use of contrast medium, such as allergy, anaphylactic shock and acute renal failure. For these and other reasons, there is still a significant number of LP shunts operations that are performed without fluoroscopic guidance.

The National Hospital for Neurology and Neurosurgery offers dedicated hydrocephalus services and receives quaternary referrals from centres situated in the UK and abroad. The present study describes the accuracy of LP shunt placement without intraoperative fluoroscopic guidance over a 10-year period.

## Methods

### Patients and procedures

An analysis of the hospital electronic records identified 163 LP shunt procedures performed without fluoroscopic guidance on patients with a diagnosis of idiopathic intracranial hypertension (IIH). They were performed between 2006 and 2016. Cases with no post-procedural imaging were excluded (56), due to the impossibility to identify the level of the proximal catheter.

### Data collection and analysis

Post-procedural imaging was reviewed and reported by two independent operators who were blinded to each other’s results and verified using visible anatomical landmarks on the x-ray, the location of the proximal end of the catheter was recorded. In all cases, the imaging used was lumbar x-ray. Clinical notes were also reviewed for those patients who had incorrect positioning of the proximal catheter to identify potentially related signs and symptoms, such as lumbar radiculopathy.

The data collection and analysis was carried out using Microsoft Excel 2010.

## Results

Between 2006 and 2016, 163 LP shunt procedures were performed on patients with IIH without intraoperative fluoroscopic guidance. After exclusion of the cases without post-procedural imaging (56), a total of 107 procedures performed on 73 patients were selected. A total of 57 patients were female and 16 were male (M:F, 1:3.5), the mean age was 41 years (± 13 SD) ranging from 16–69 years.

**Figure 1.**
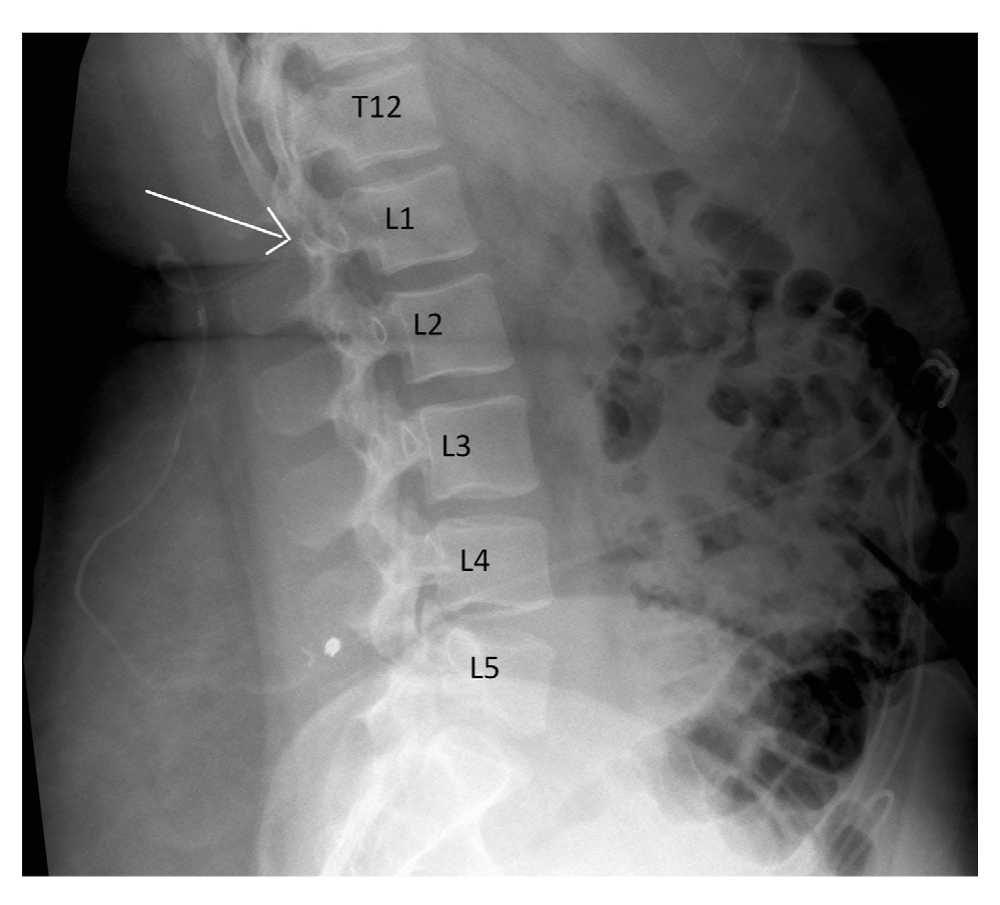
Lateral lumbar x-ray showing incorrect level of lumboperitoneal shunt insertion.

The review of all post-procedural imaging showed that in 17 cases (15.8%) patients had the proximal catheter placed at the level of L1/L2 or above (T12/L1, 1.8%; L1/2, 14.0%) ( Figure 1). On the other hand, in 94 cases (84.2%), patients had the proximal tip of the catheter placed at the level of L2/L3 or below (L2/3, 33.0%; L3/4, 37.4%; L4/5, 12.0%; L5/S1, 1.8%) ( Table 1).

**Table 1.** Number of cases of lumboperitoneal shunt insertion according to the level of insertion.

Level of insertion	Number of cases (%)
T12/L1	2 (1.8)
L1/L2	15 (14.0)
L2/L3	35 (32.7)
L3/L4	40 (37.4)
L4/L5	13 (12.0)
L5/S1	2 (1.8)

An analysis of the clinical notes of the patients who had mispositioned LP shunts was carried out for a minimal post-operative period of one year. None of the patients complained of signs or symptoms related to possible distal spinal cord damage.

X-rays showing the final position of the lumboperitoneal (LP) shunt in patients that underwent LP shunt insertion without fluoroscopic guidance Click here for additional data file. Copyright: © 2017 Al-Rashed S et al. 2017 Data associated with the article are available under the terms of the Creative Commons Zero "No rights reserved" data waiver (CC0 1.0 Public domain dedication).

## Discussion

This study demonstrates that, without intraoperative fluoroscopic guidance, an LP shunt insertion procedure can lead to a mispositioned proximal catheter in 15.8% of cases. Despite none of our patients presenting with any signs or symptoms of spinal cord damage, this risk needs to be considered when performing this procedure “blindly”.

One of the biggest challenges in performing LP shunts in IIH patients is often related to their habitus. It is in fact recognised that a strong association between IIH and obesity exists ^16^. Approximately 70–80% of IIH patients are obese and over 90% are overweight ^16^. In this group of patients finding the anatomical landmarks, maintaining them and inserting the lumbar catheter at the correct level, can represent a technical challenge; this is especially true when the procedure is performed without fluoroscopy guidance.

We suggest that the use of intra-operative imaging guidance should be adopted: this practice could reduce the incidence of mispositioned LP shunts and therefore decrease the risk of significant spinal cord damage, which may have serious, irreversible consequences.

The results of this series must be interpreted considering the limitations of the nature of any retrospective study. It could be argued that results achieved by our unit could vary markedly from those achieved at other units. We also do not take into account for operator experience, which may be partially responsible for the differences in success rate, and again may vary from individual to individual. Ultimately, to prove the efficacy and benefits of intraoperative imaging for LP shunt insertion, large, prospective, randomised controlled studies should be performed.

## Conclusions

While this series is too small to conclude whether intraoperative imaging should be used to minimize the risk of misplaced proximal LP shunt catheters, it prepares the basis for further prospective studies. Our results suggest that LP shunt insertion without fluoroscopic guidance has a 15.8% risk of misplacement of the end position of the proximal catheter, and for this reason the use of intraoperative image guidance is suggested to reduce the risk of spinal cord damage and its potentially catastrophic consequences, although no spinal cord injury was noted as a result of misplacement in our patient population.

## Ethical statement

Ethical approval and registration was obtained from the National Hospital for Neurology and Neurosurgery.

This study was performed as part of an audit to analyse the current practice against the department policy standards.

## Data availability

The data referenced by this article are under copyright with the following copyright statement: Copyright: © 2017 Al-Rashed S et al.

Data associated with the article are available under the terms of the Creative Commons Zero "No rights reserved" data waiver (CC0 1.0 Public domain dedication). 

**Dataset 1: X-rays showing the final position of the lumboperitoneal (LP) shunt in patients that underwent LP shunt insertion without fluoroscopic guidance.** Each page of the dataset indicates a different procedure. doi, 10.5256/f1000research.11089.d154686 ^17^
