# Supplementary material for: Lumboperitoneal shunt insertion without fluoroscopy guidance: Accuracy of placement in a series of 107 procedures
Source: F1000Res. 2017 Jun 30;6:565. Originally published 2017 Apr 25. [Version 2] doi: 10.12688/f1000research.11089.2 (PMC5506531; doi:10.12688/f1000research.11089.2)

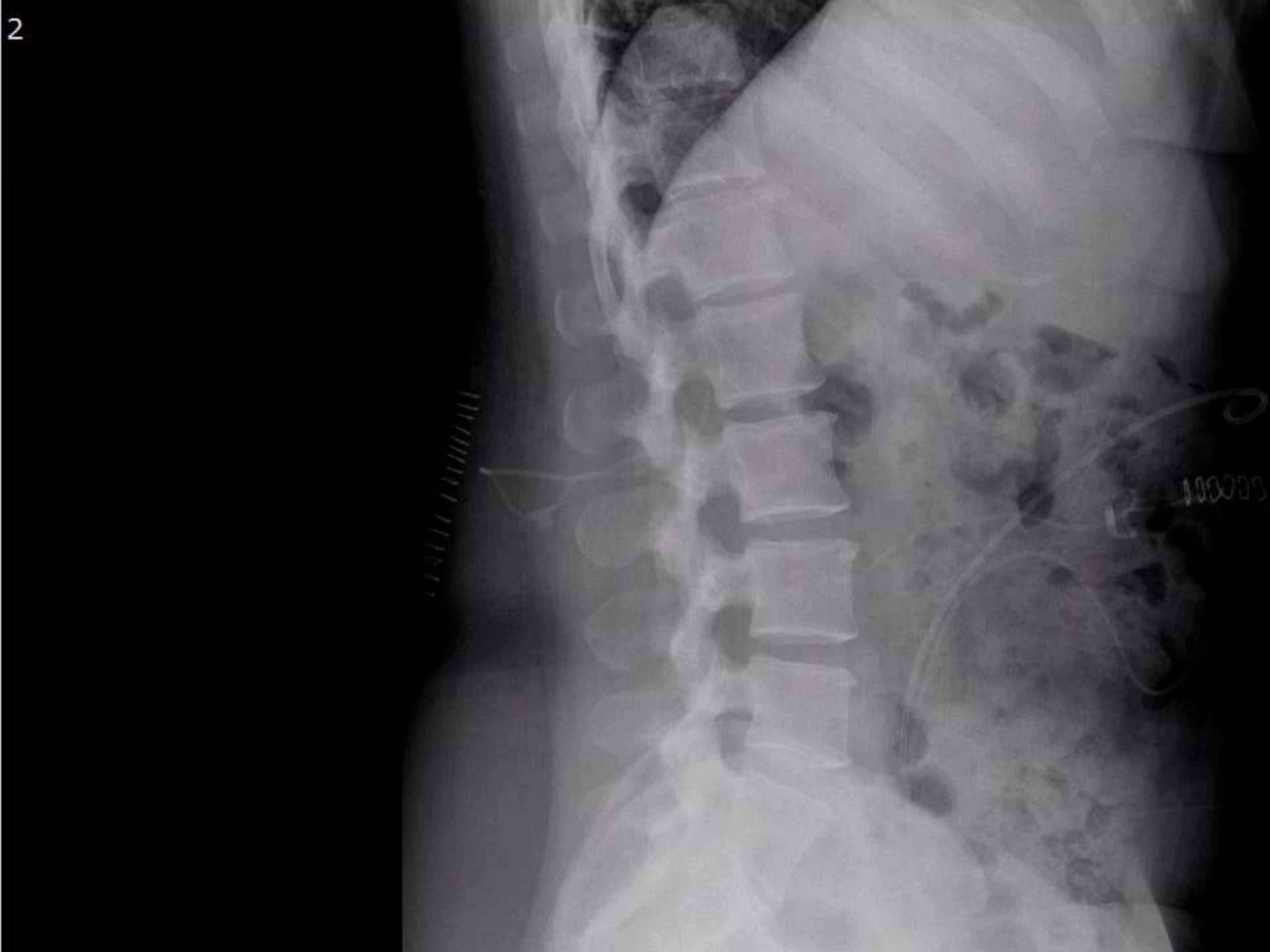
2

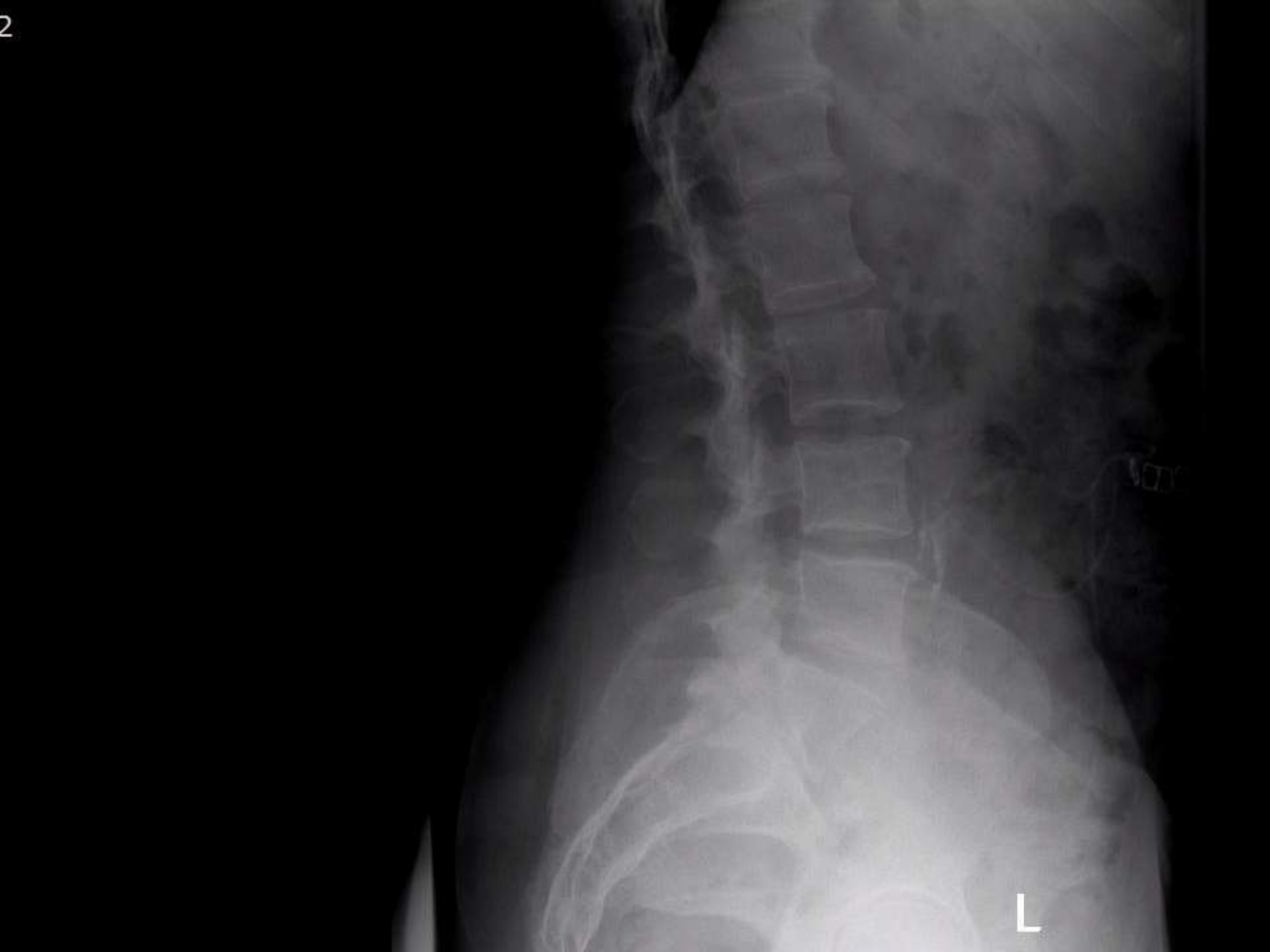
2
L

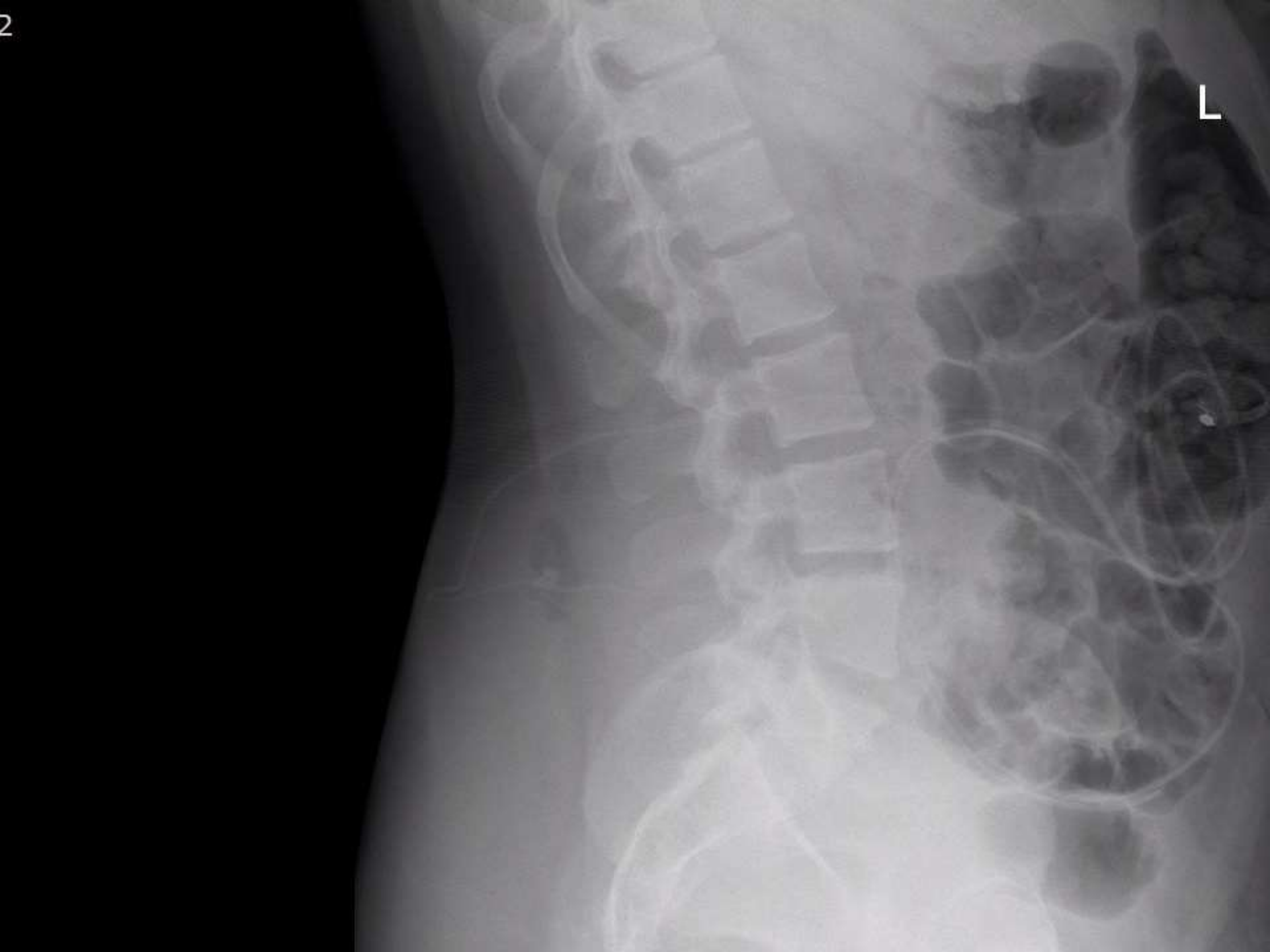
2
L

2

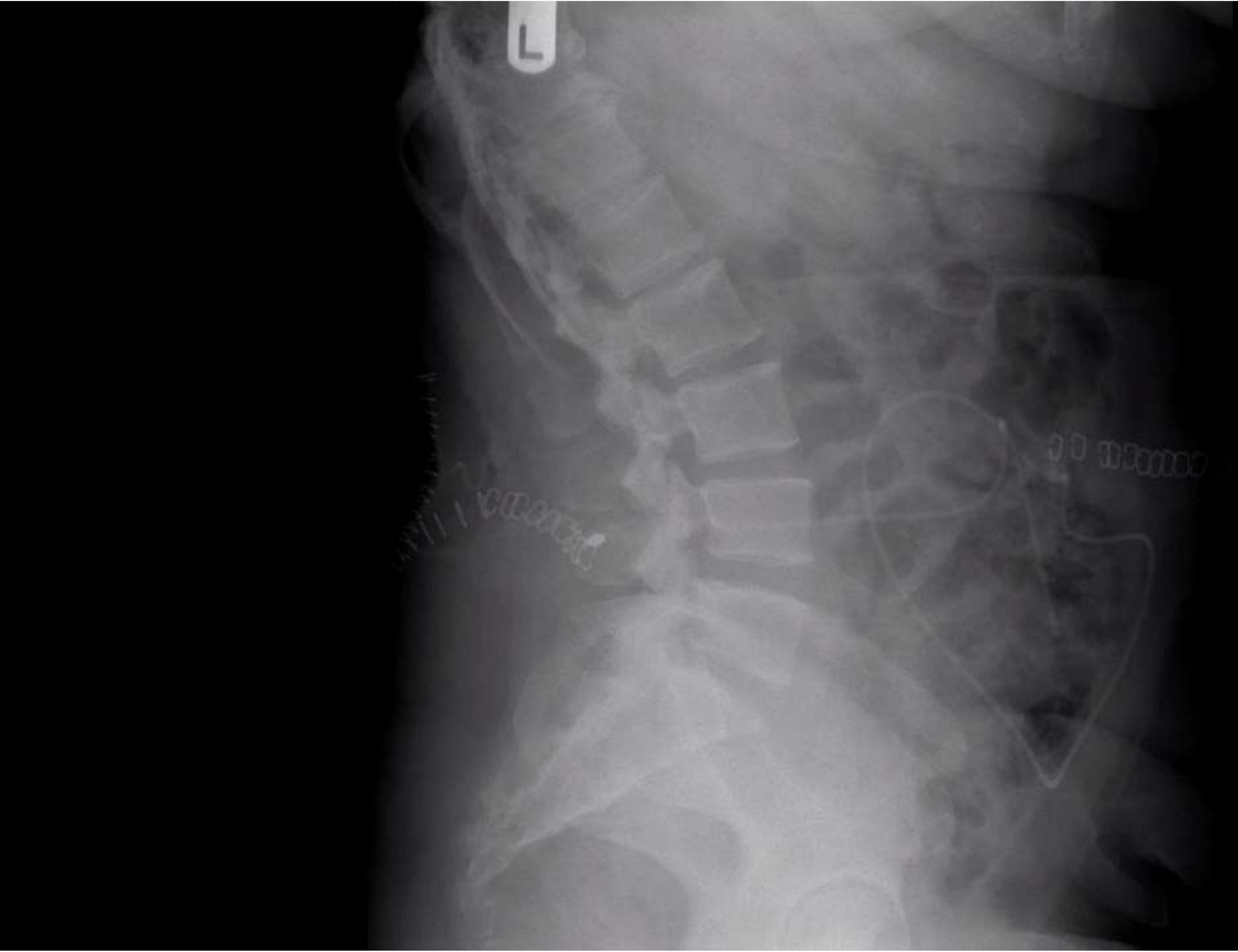

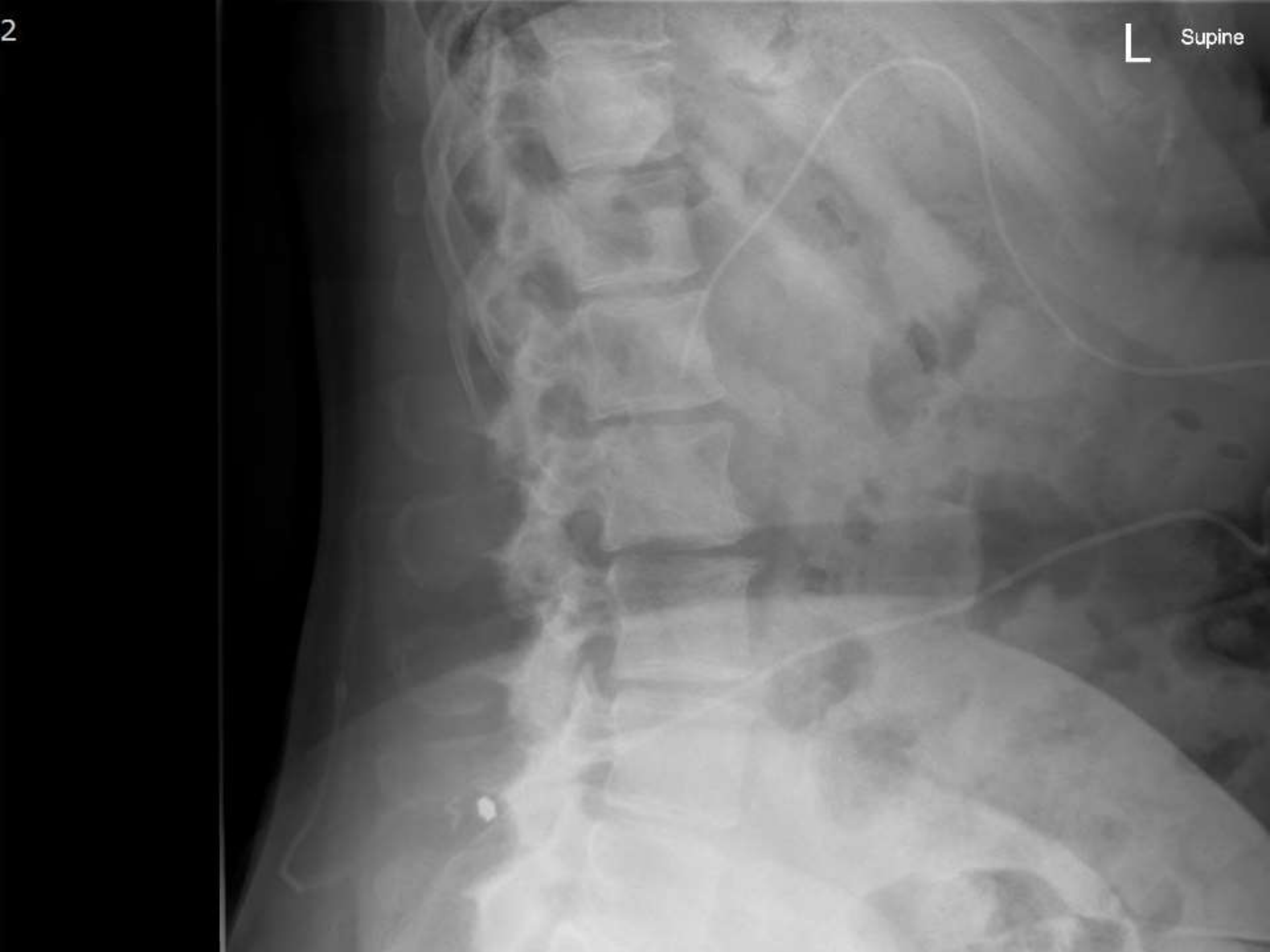
2
L
Supine

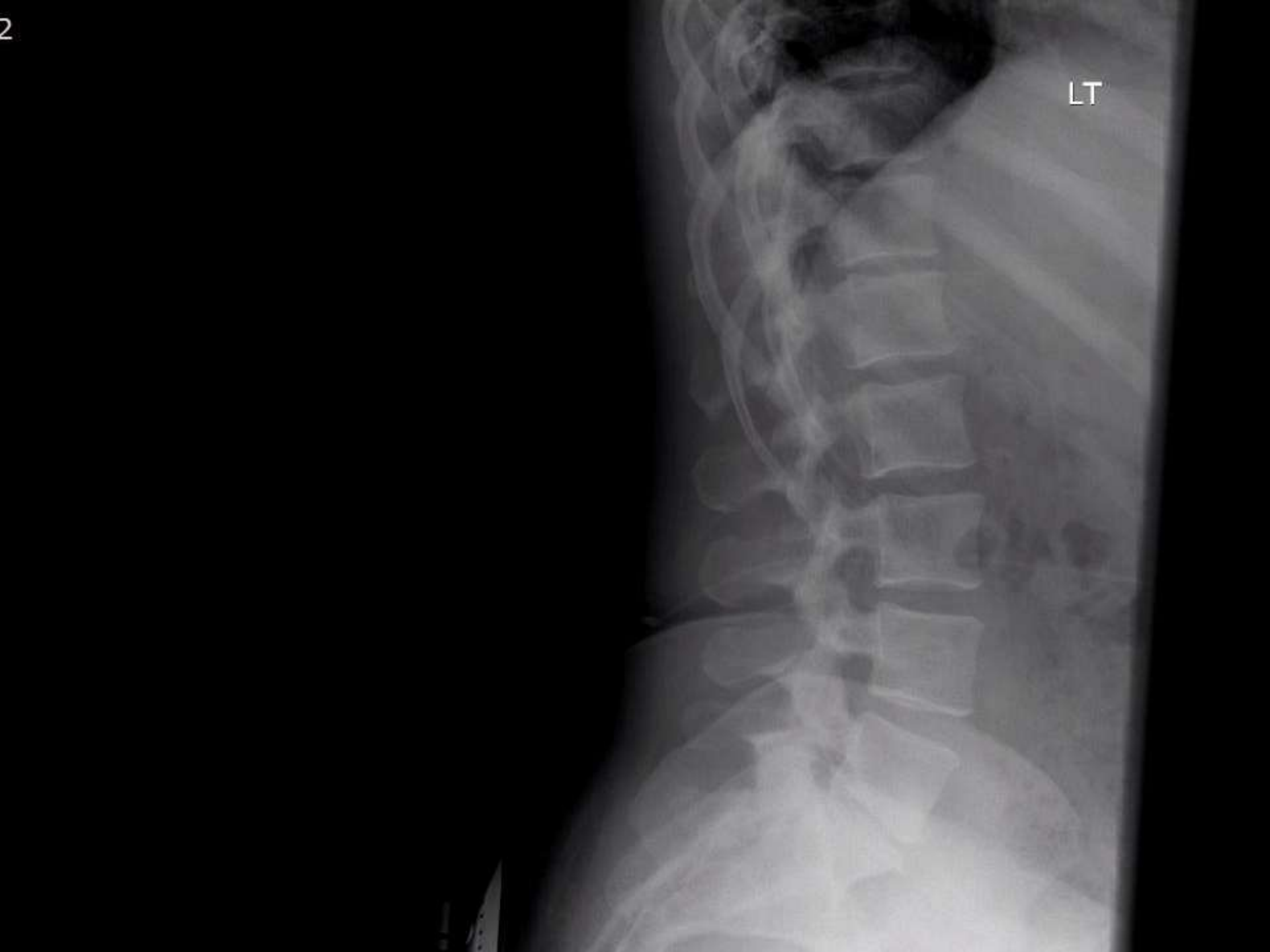
LT

1

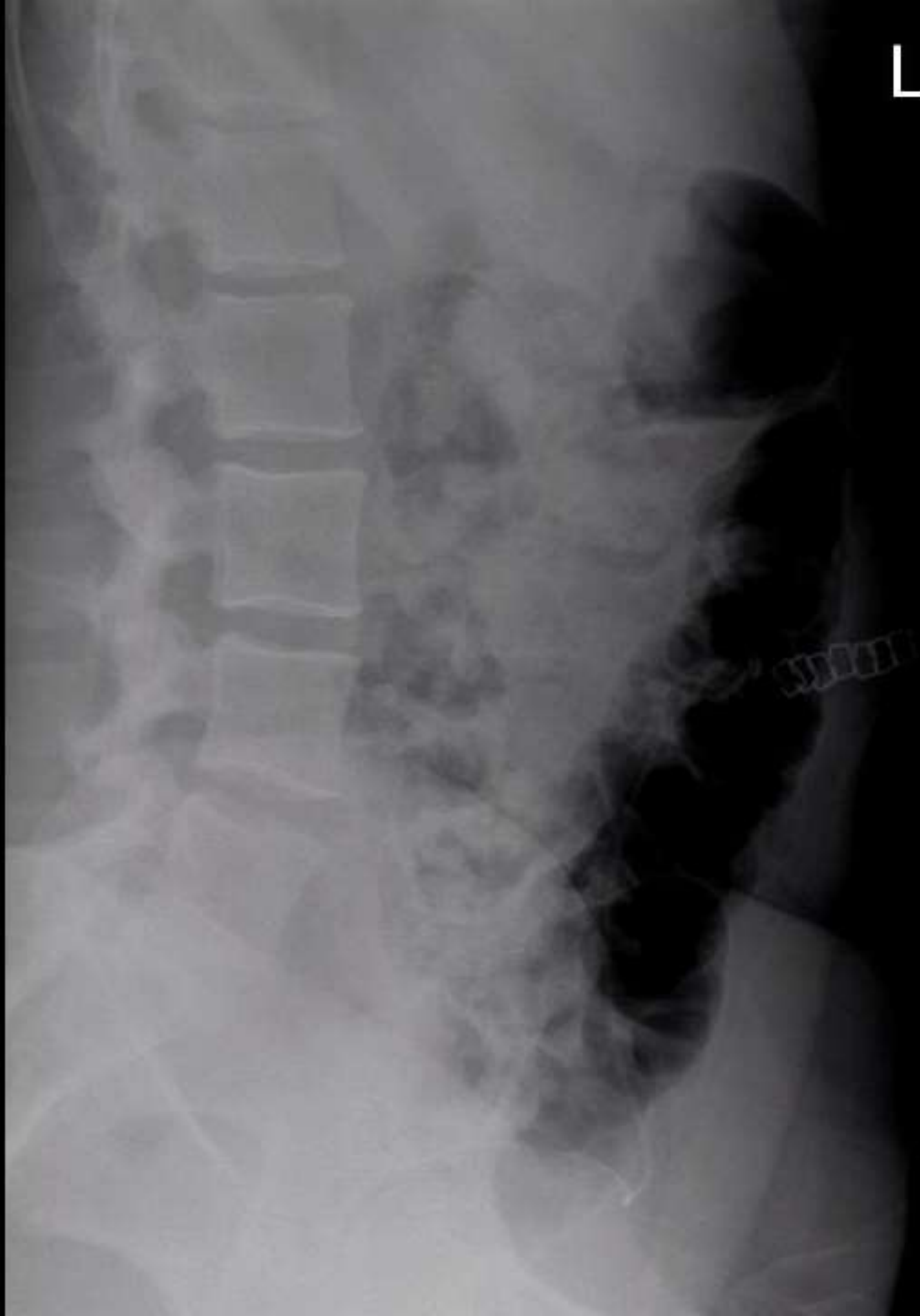

3

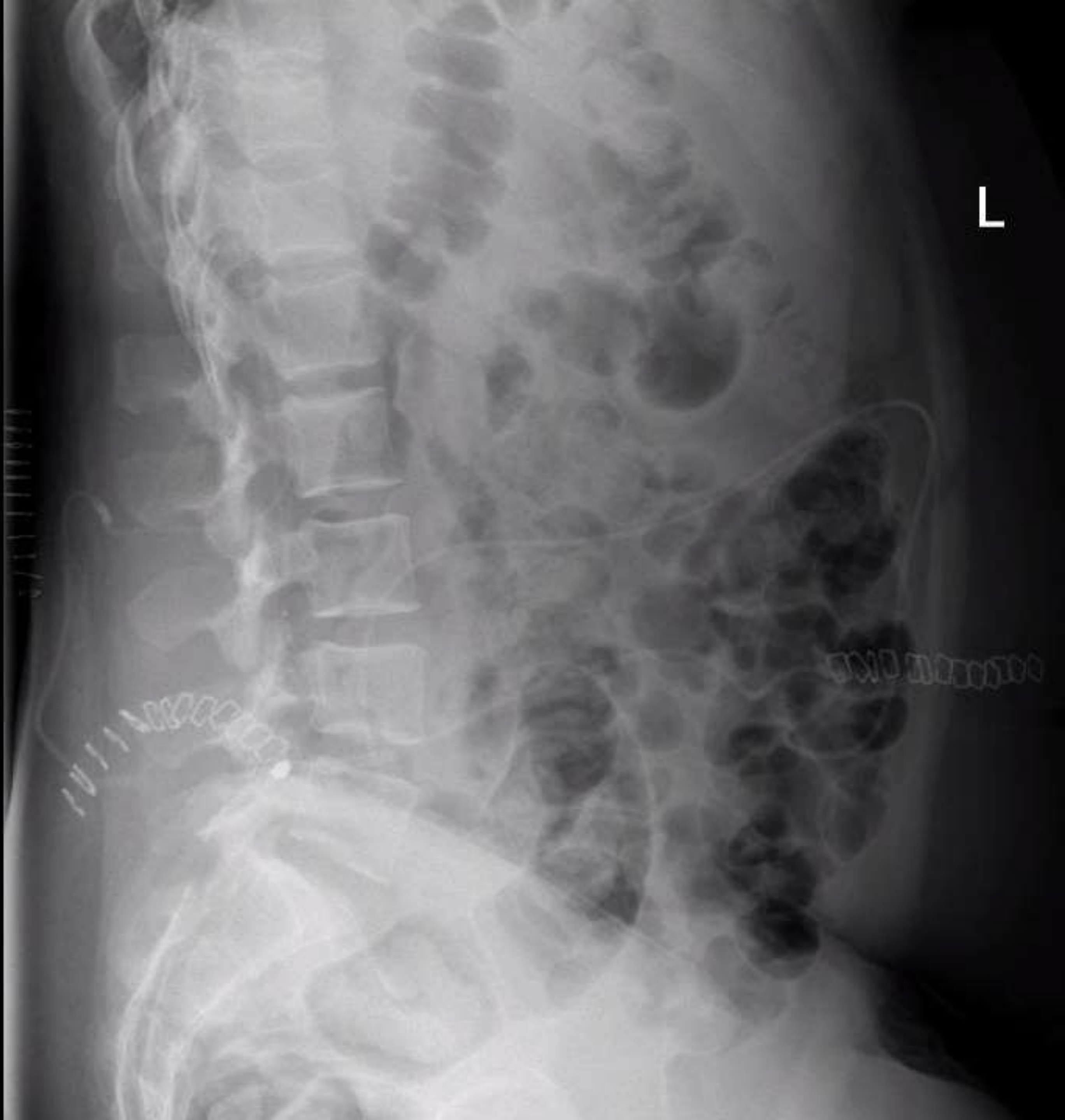

2

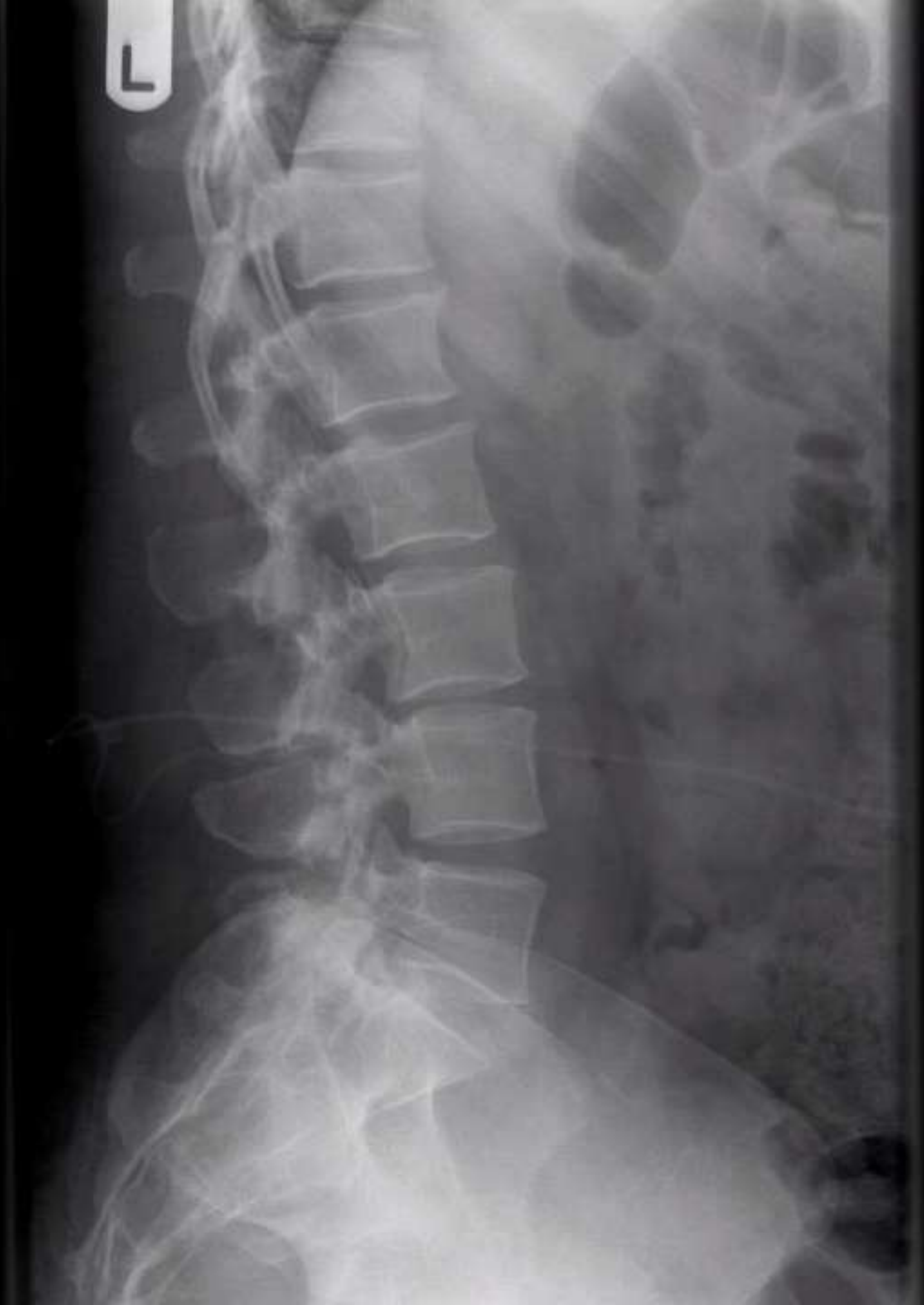

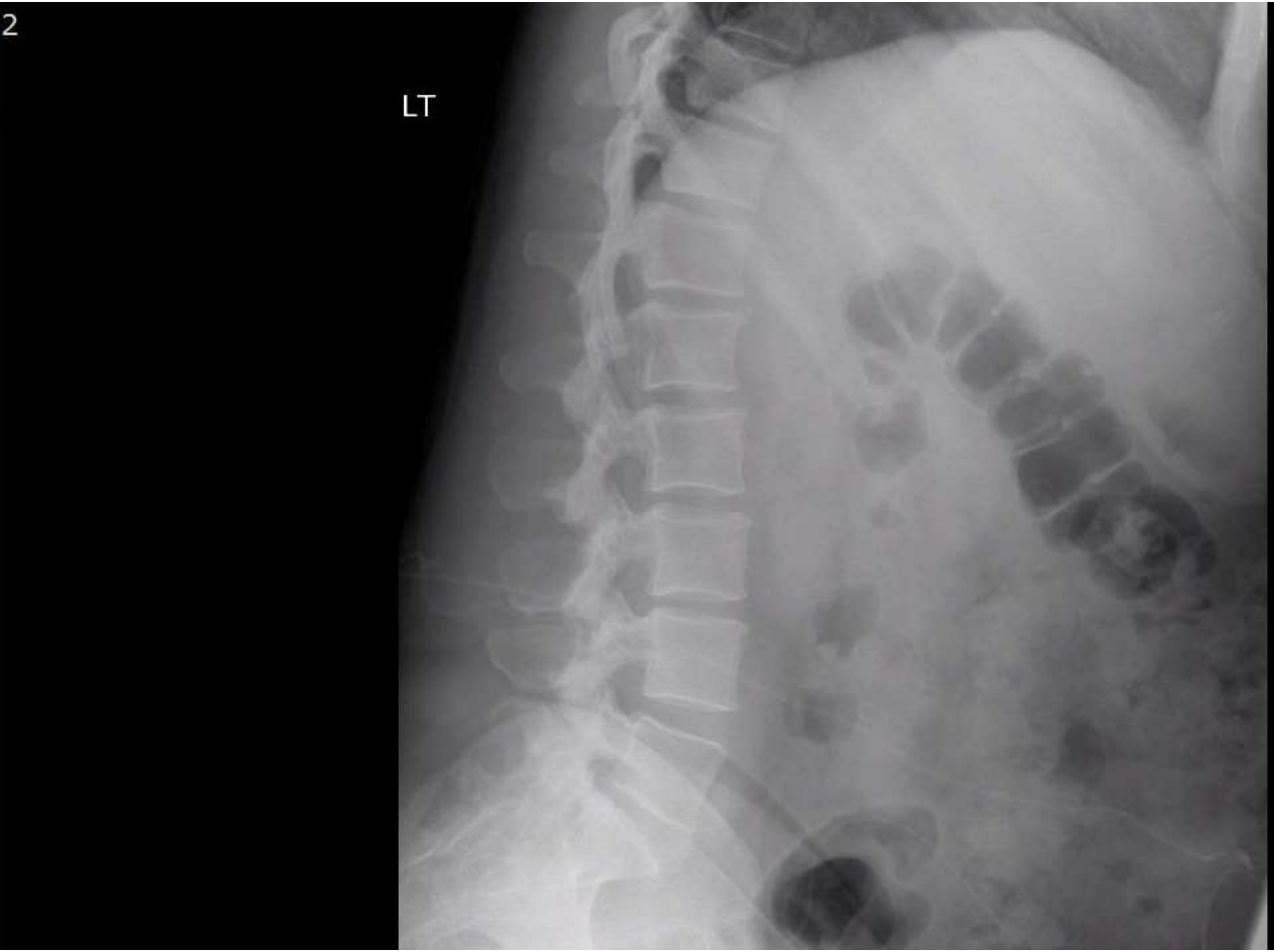
2
LT

3

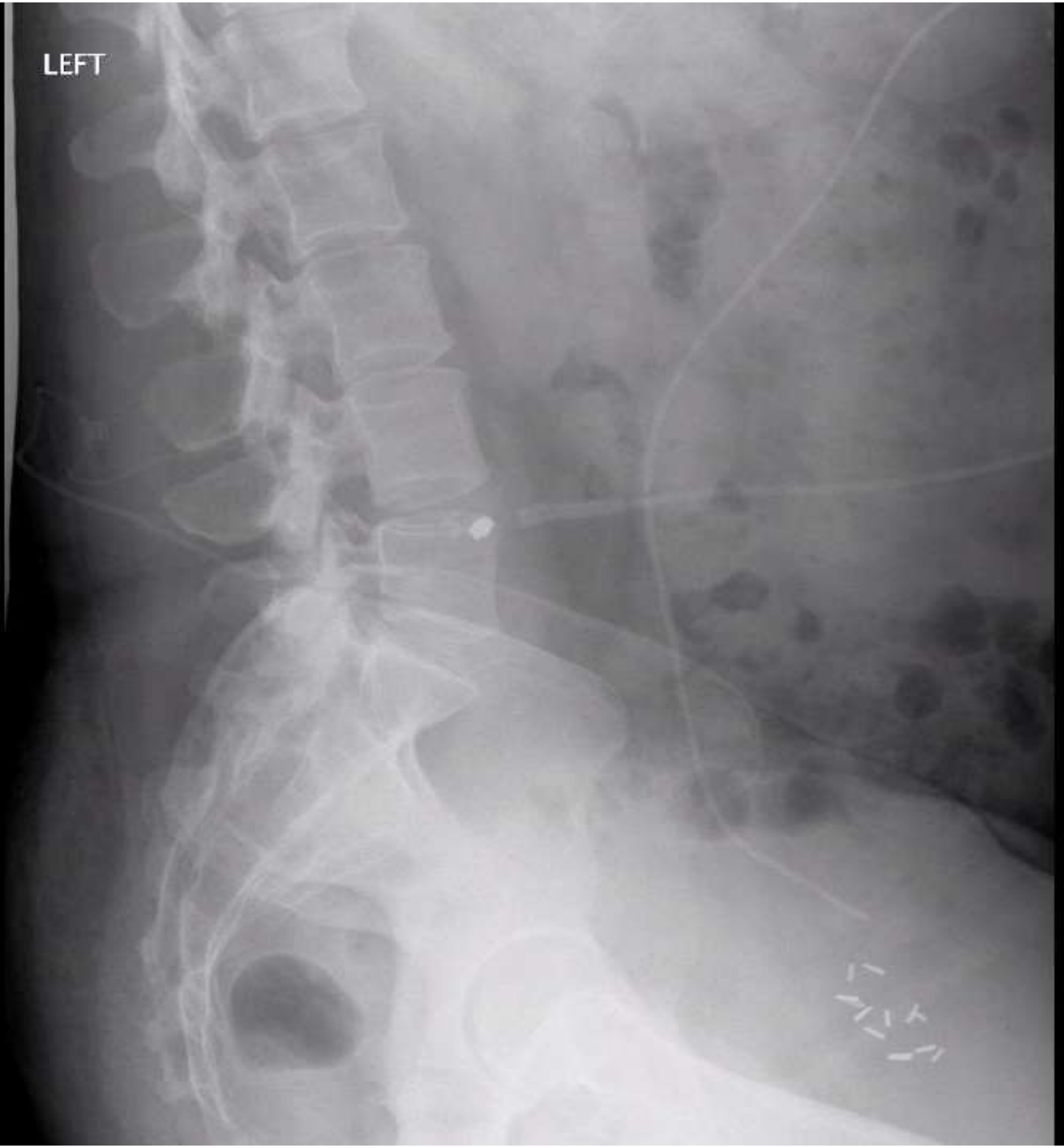

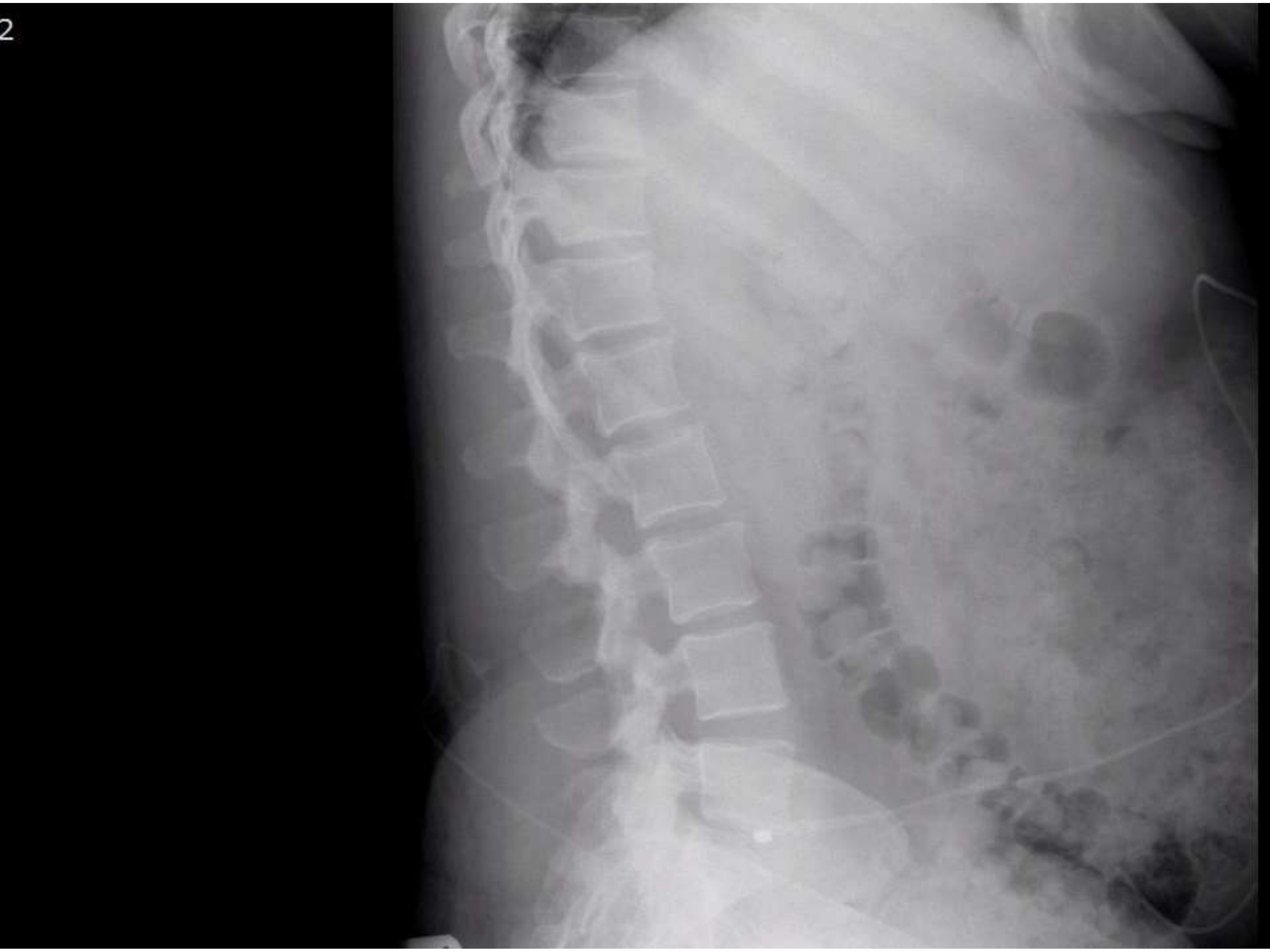
2

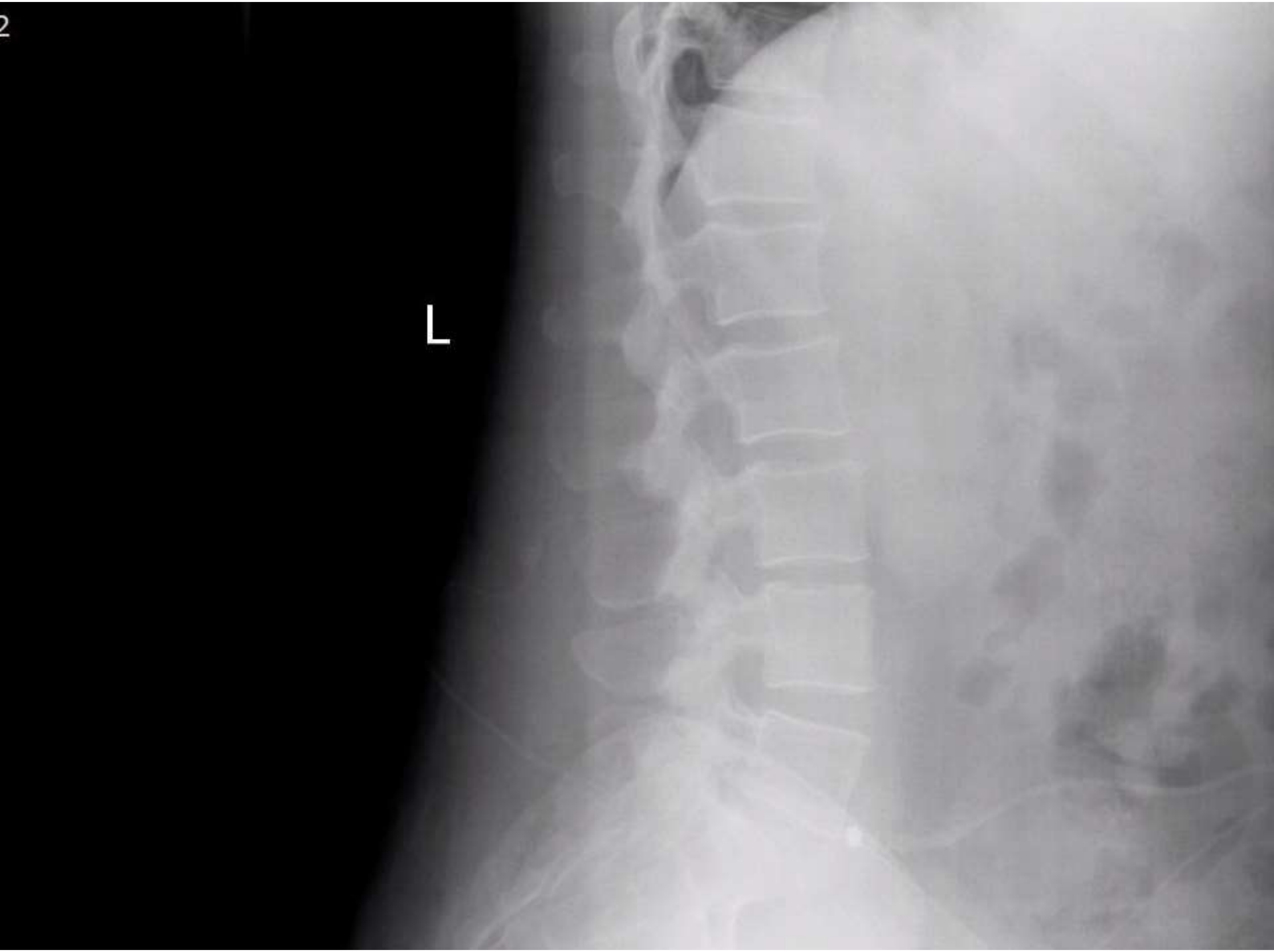
L

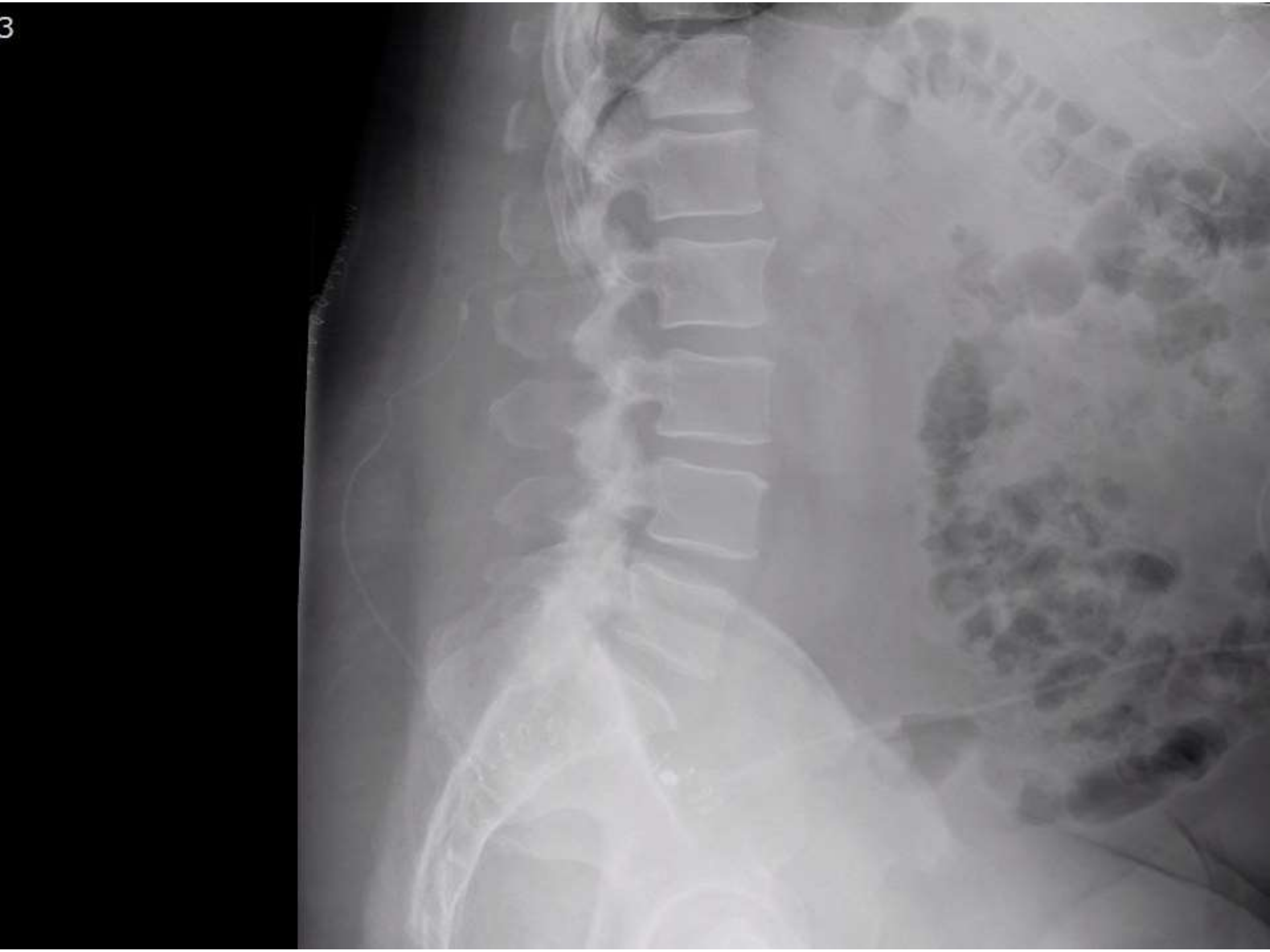
3

2

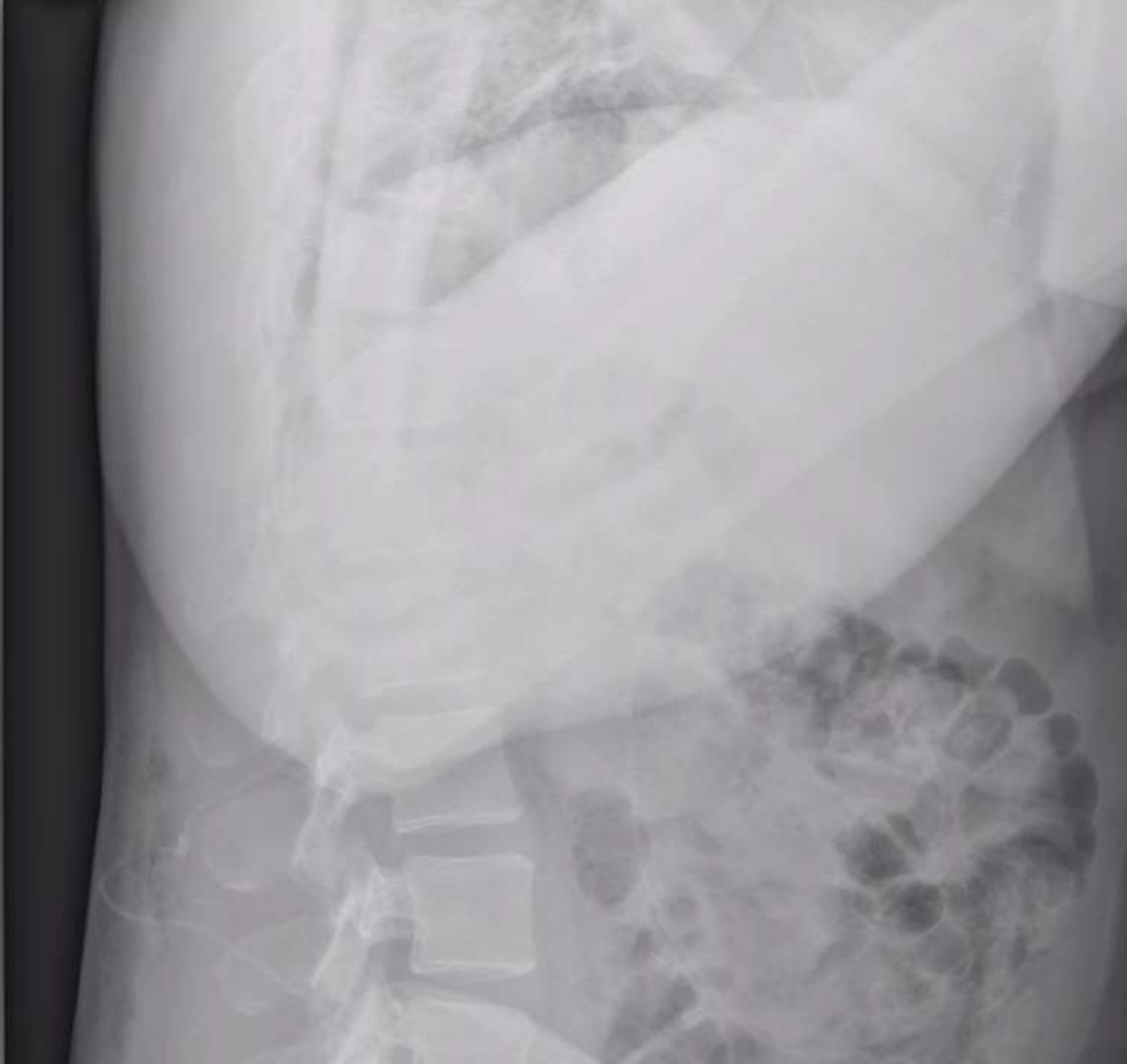

2

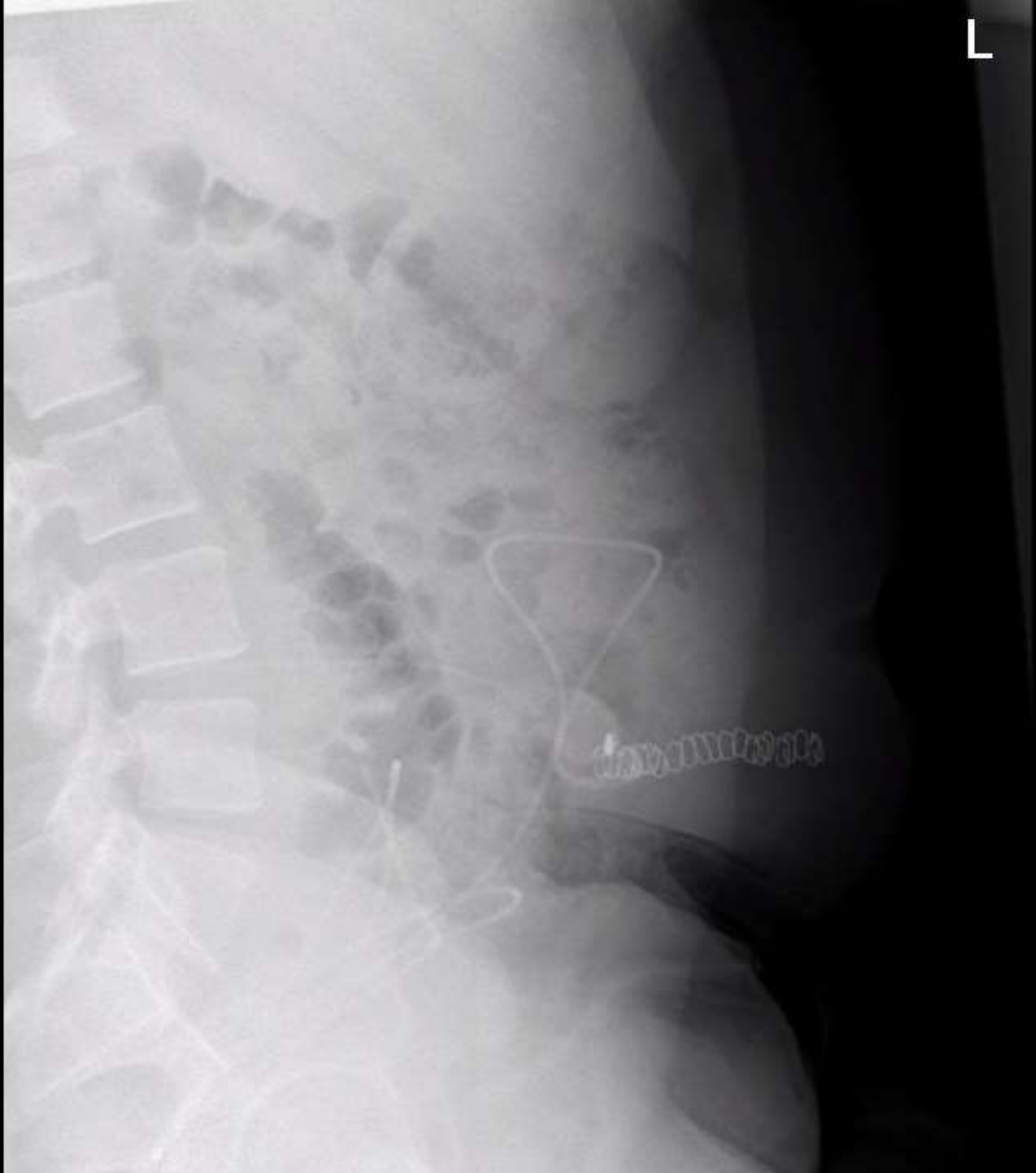

1

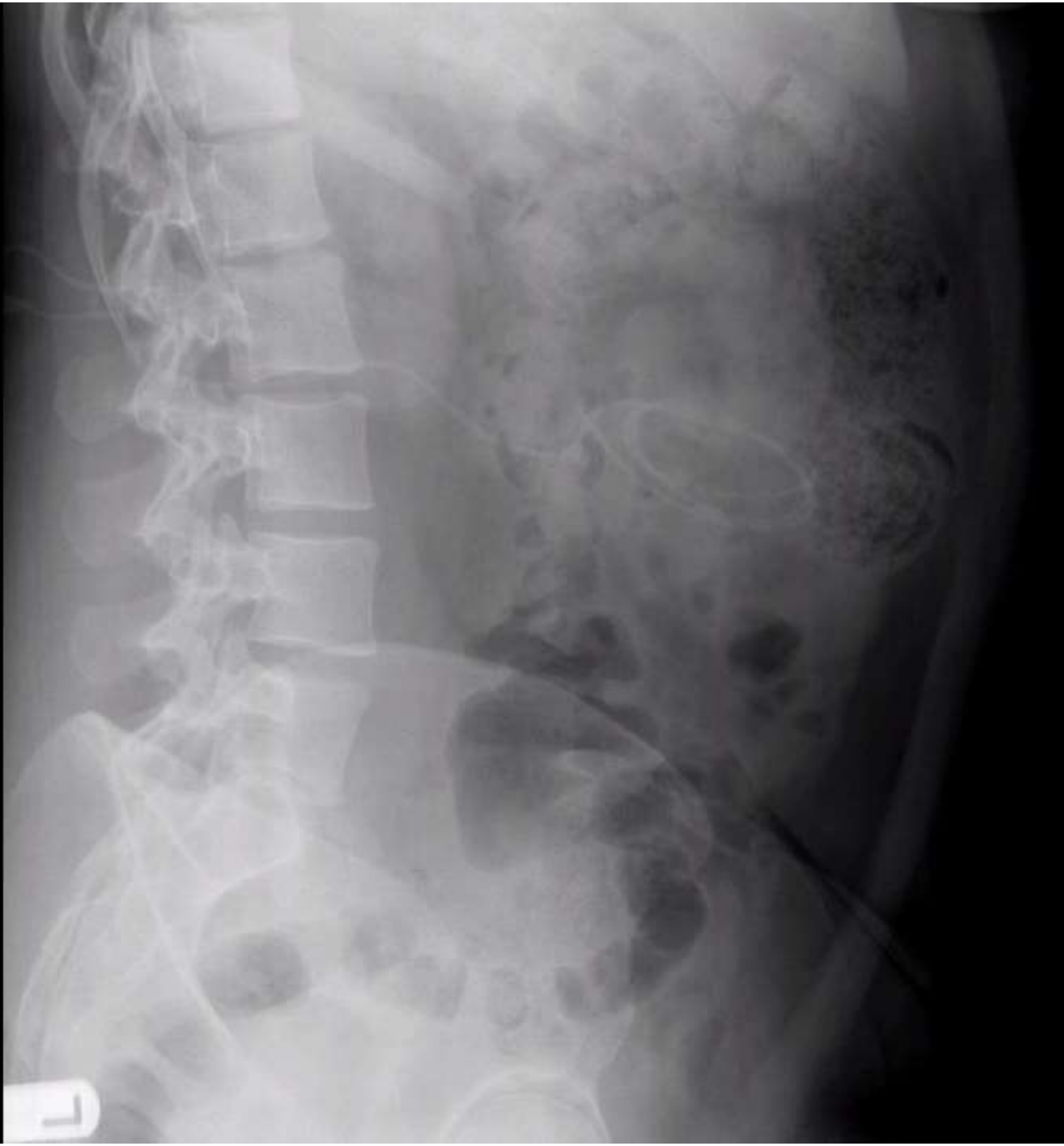

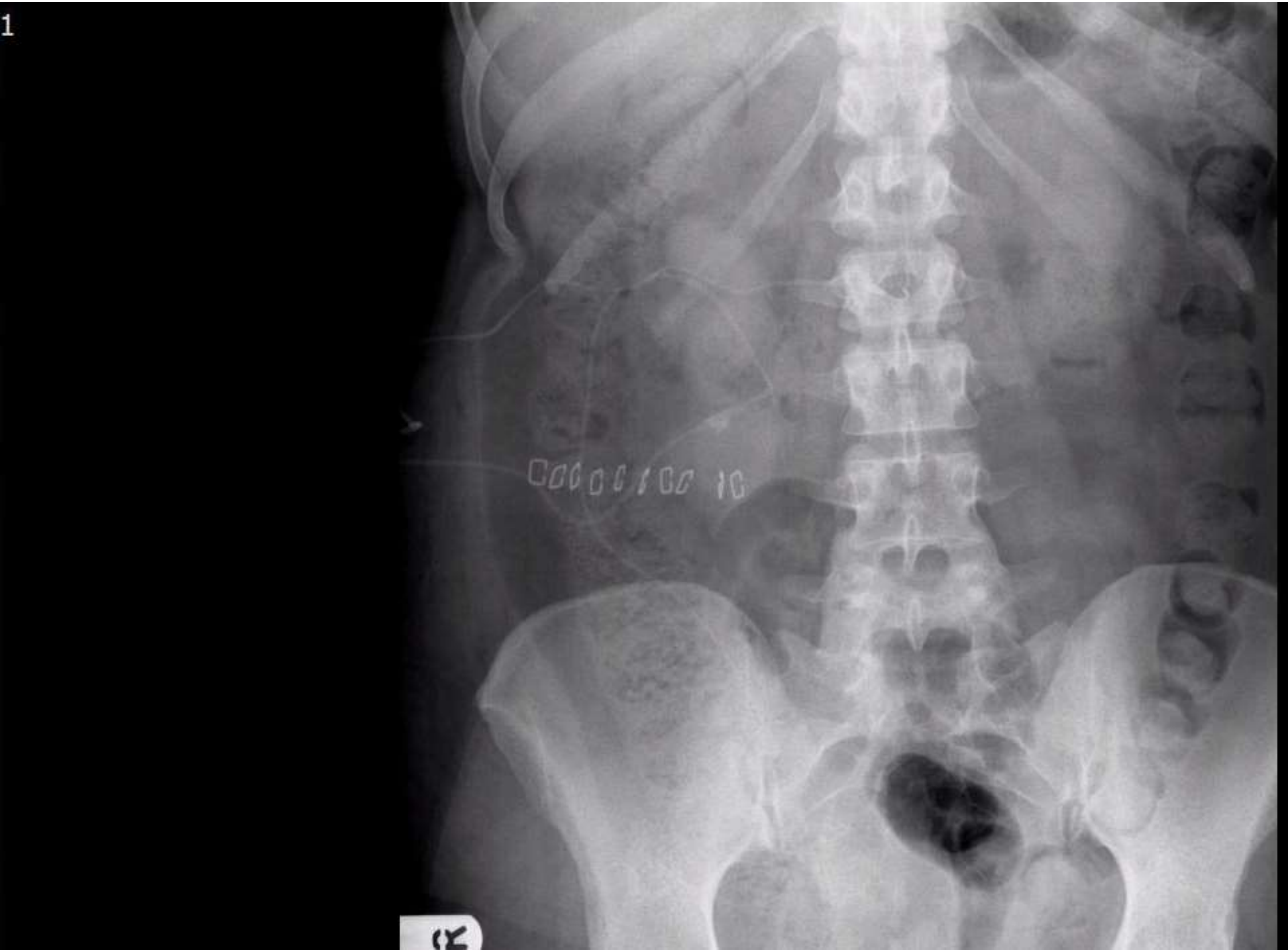
1

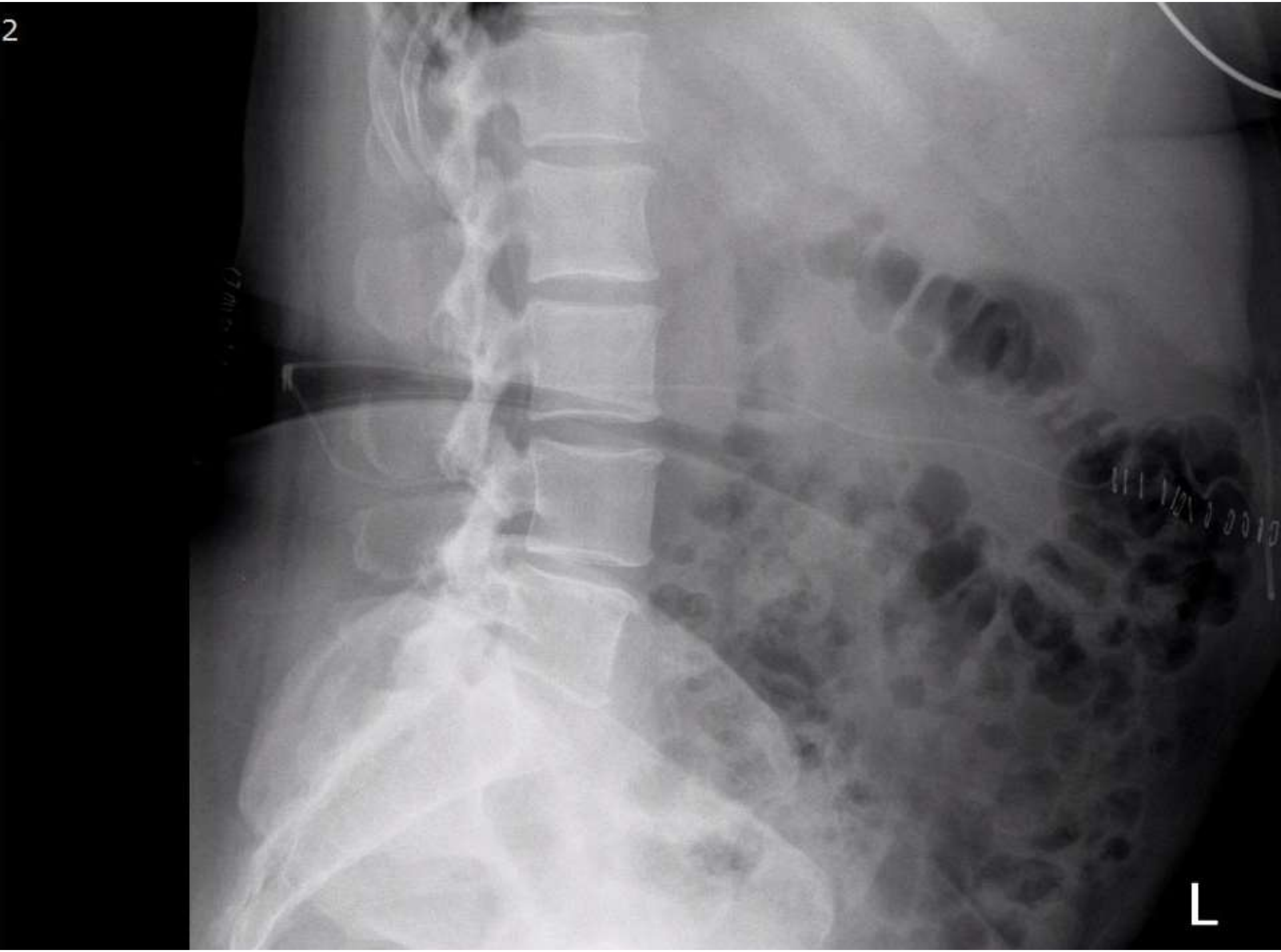
2
L

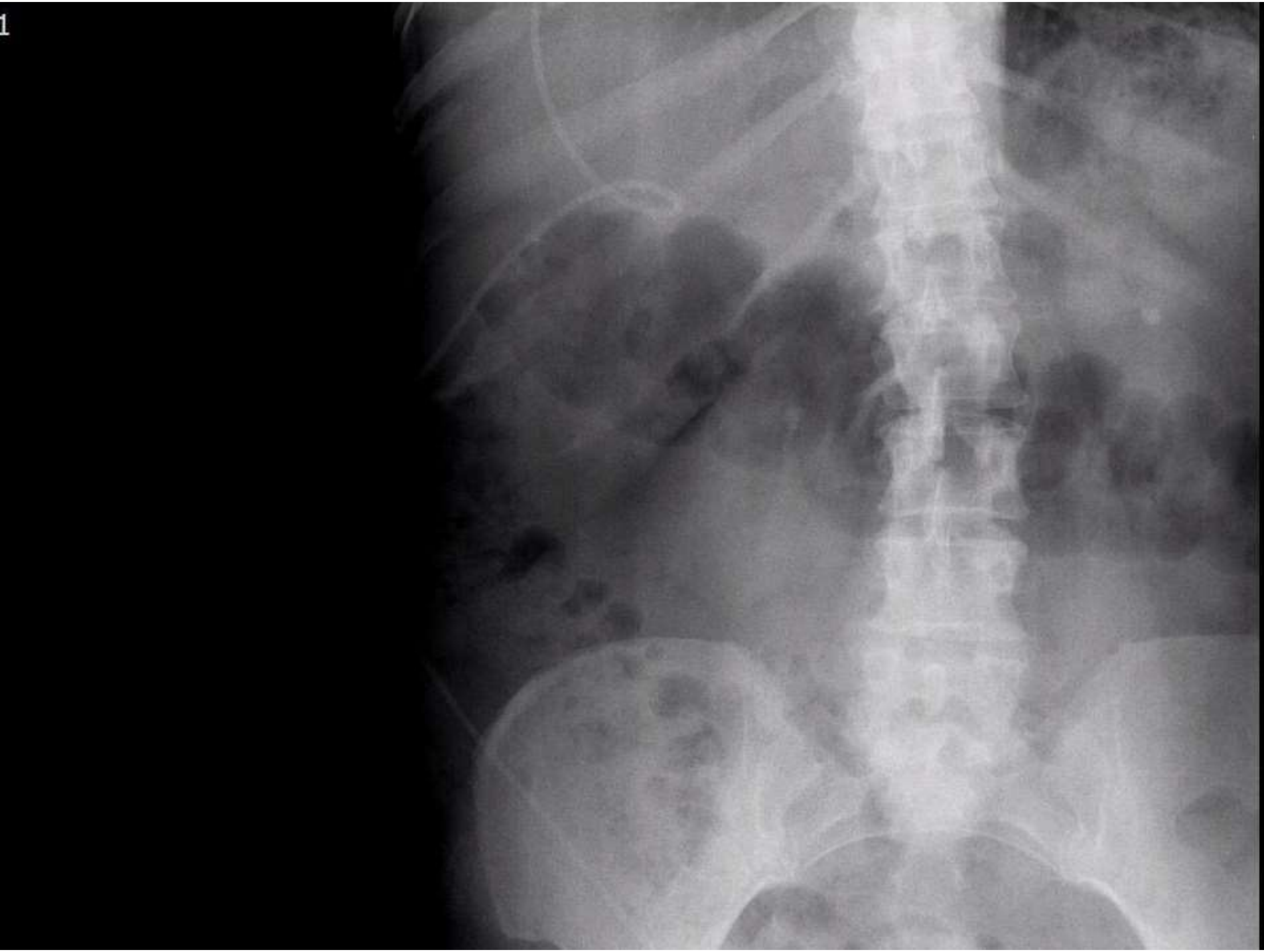
1

1

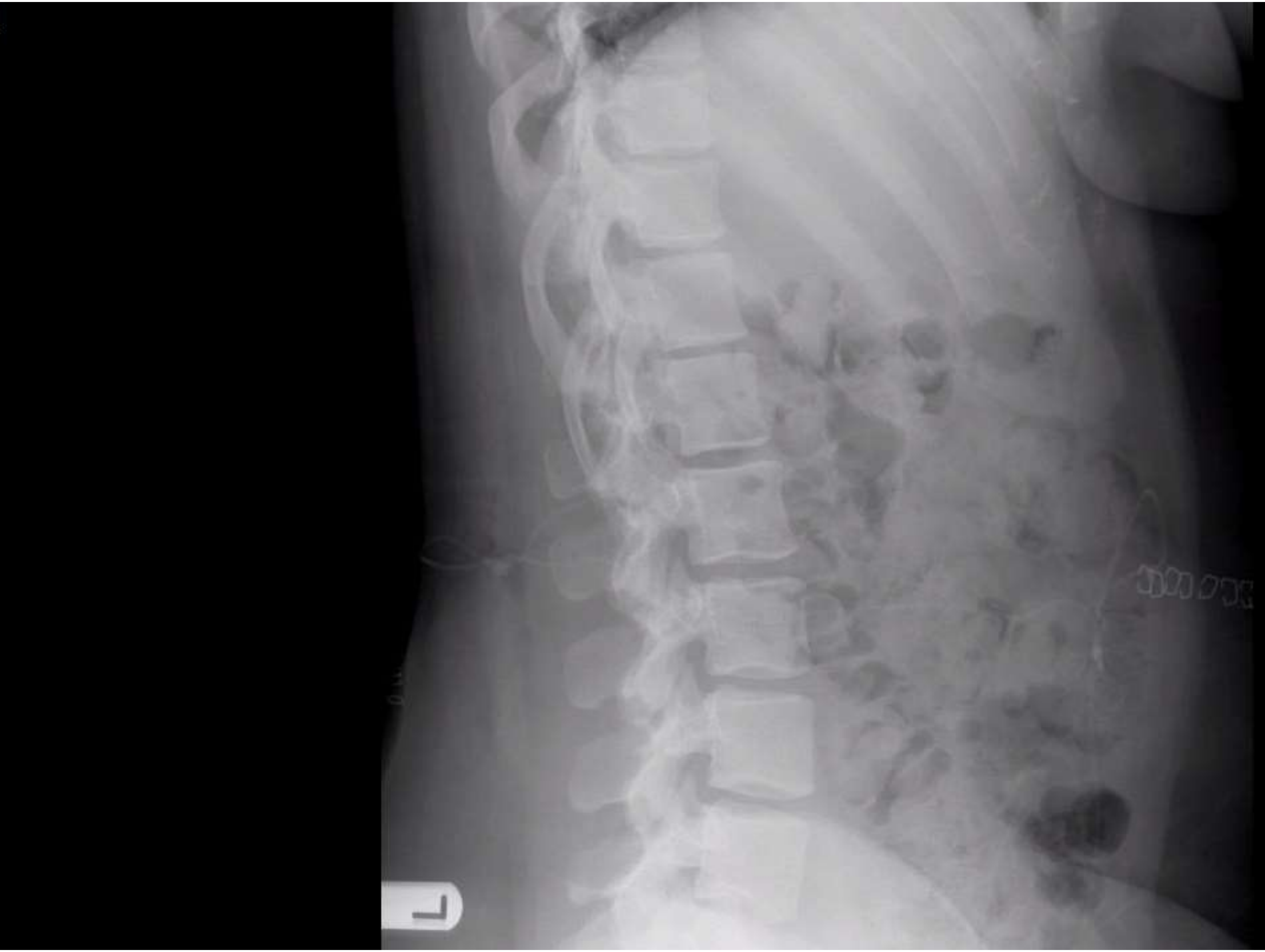

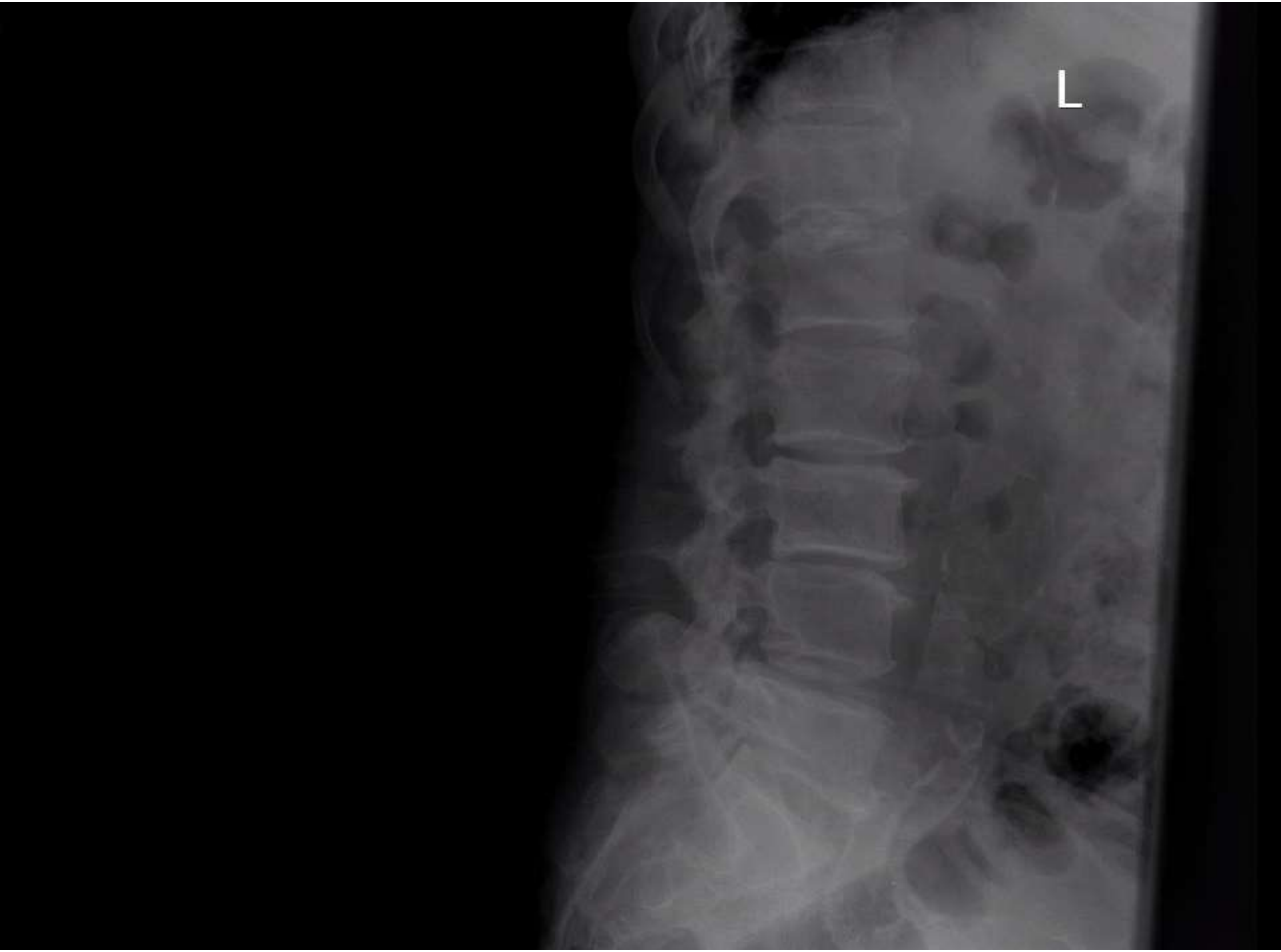
L

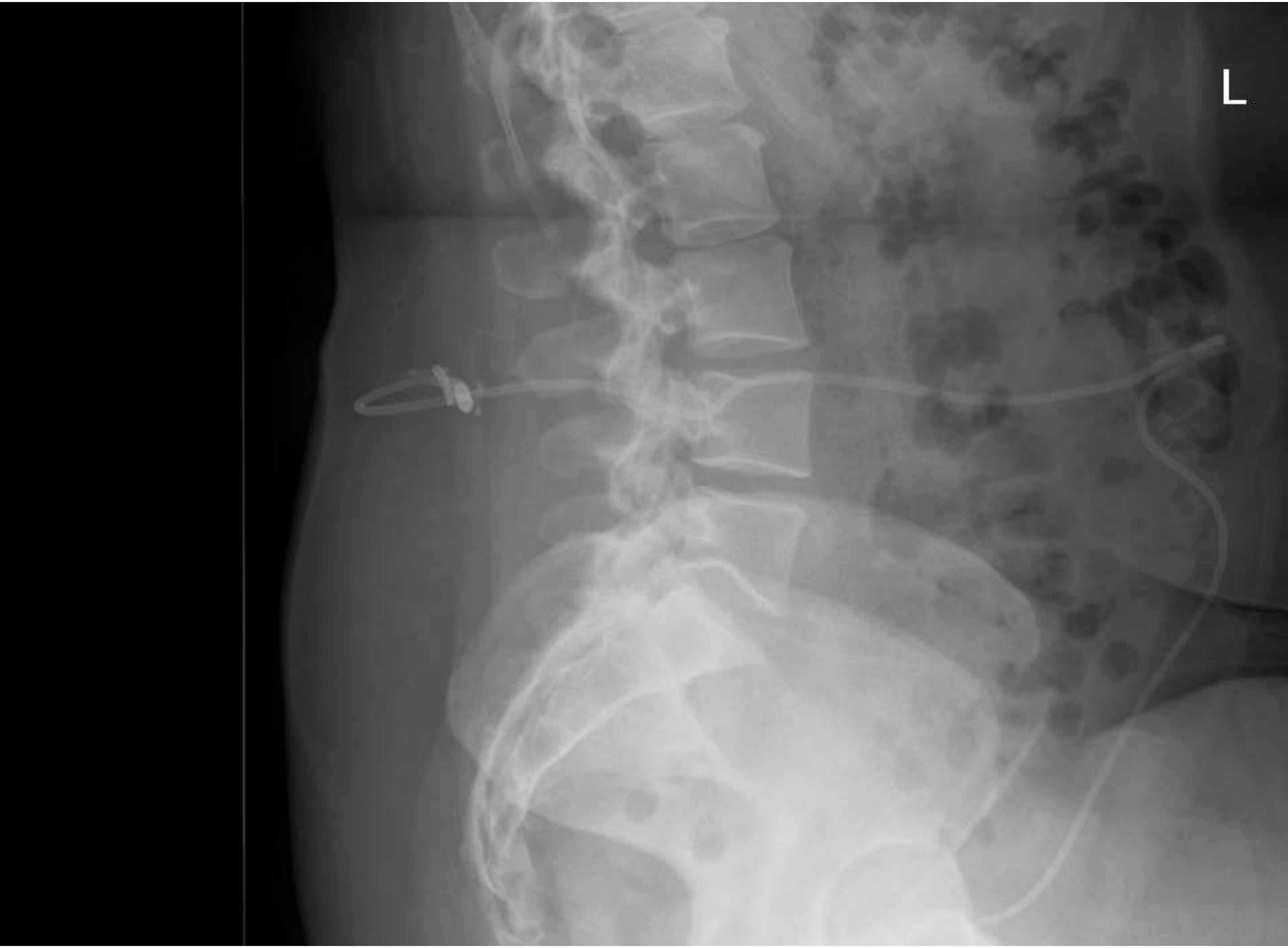
L

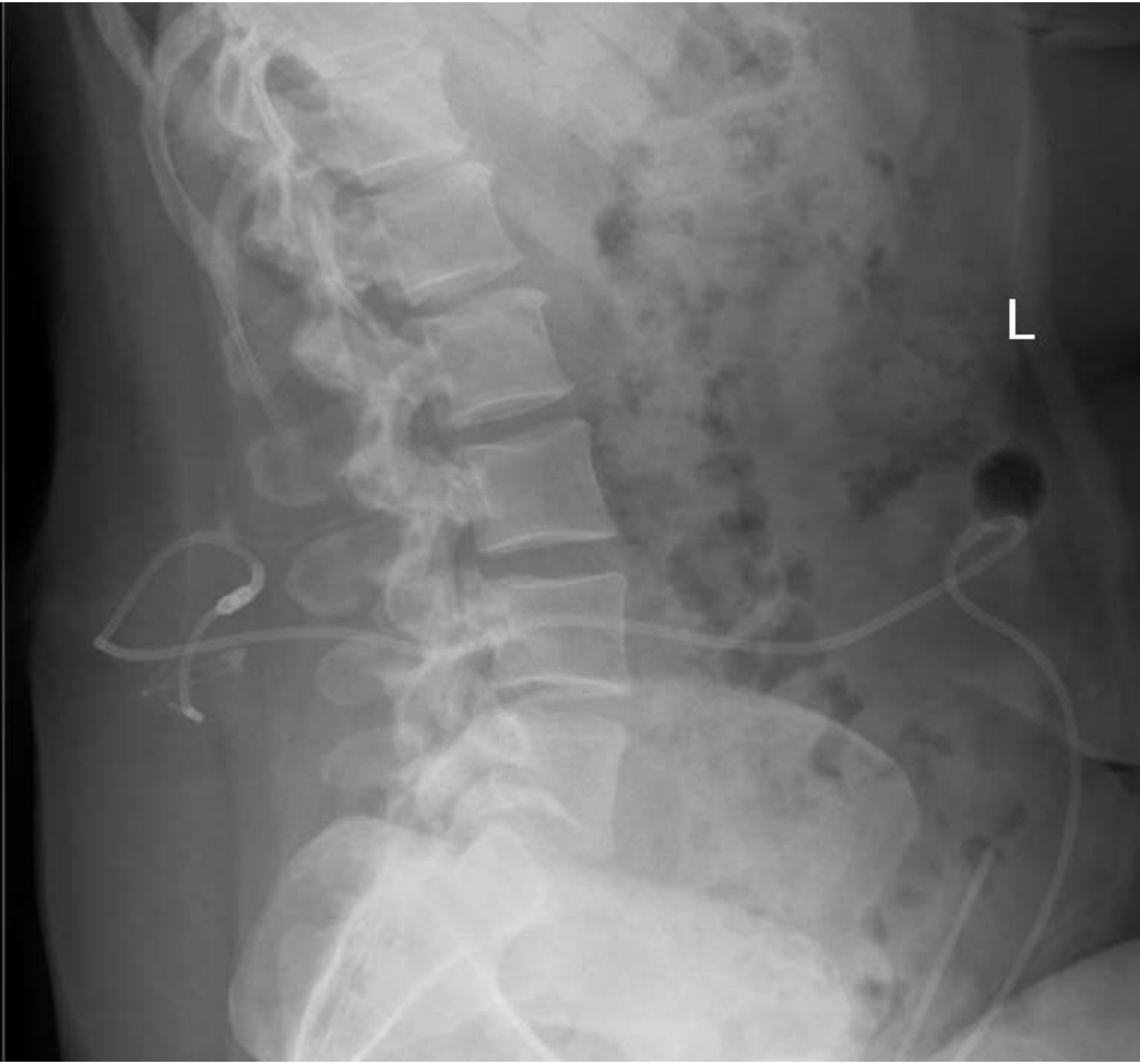
L

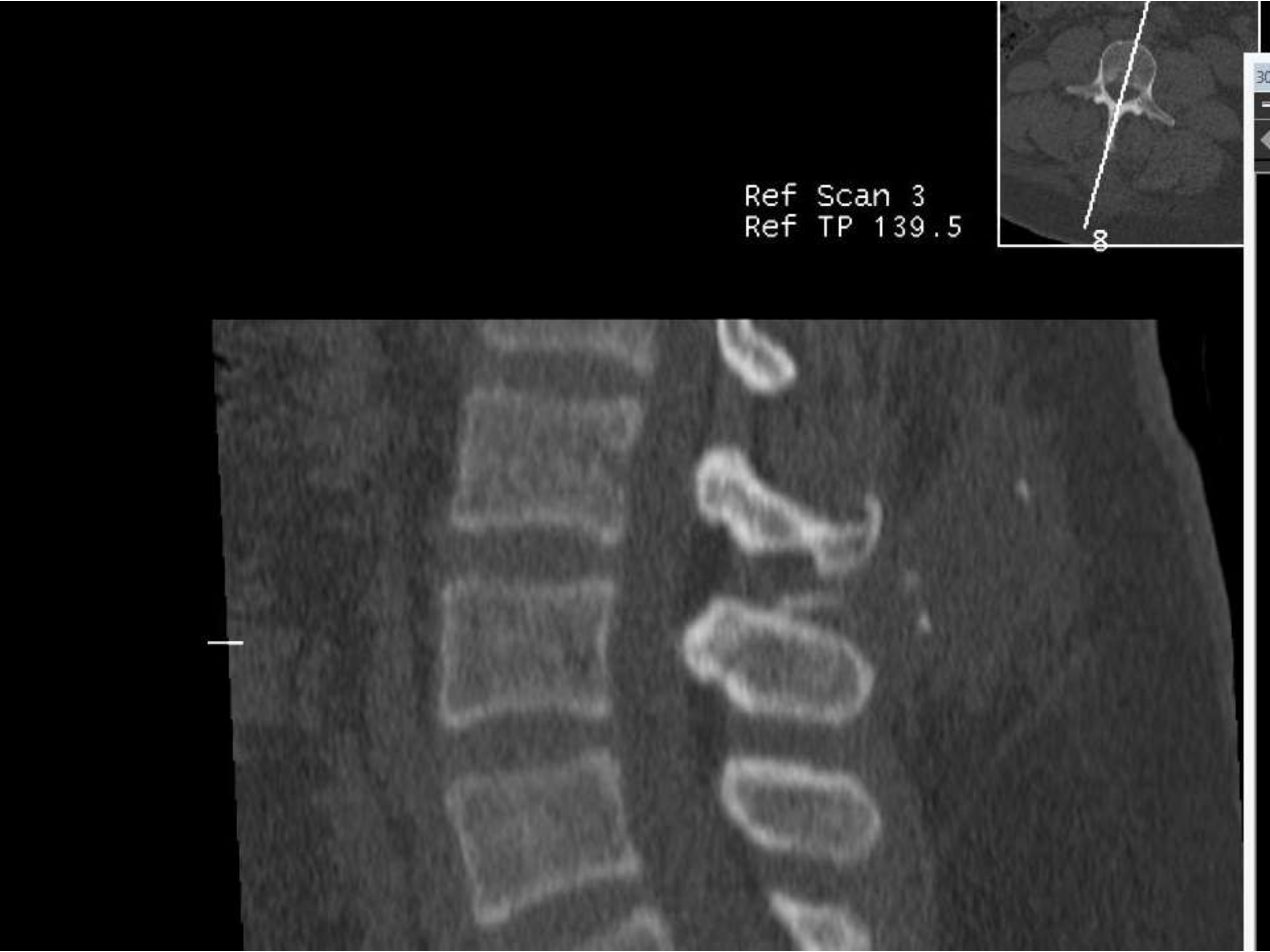
Ref Scan 3
Ref TP 139.5
8

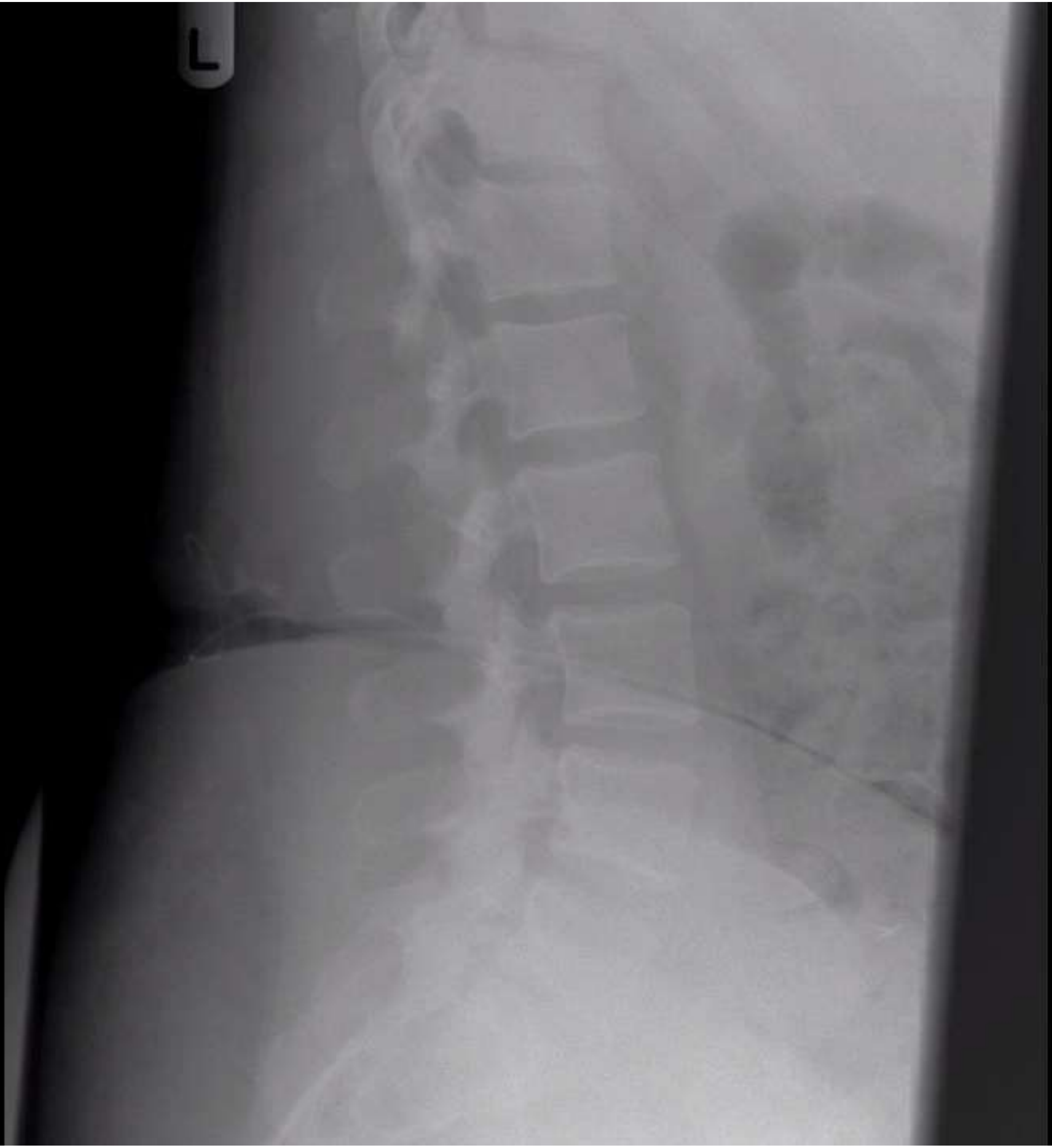
L

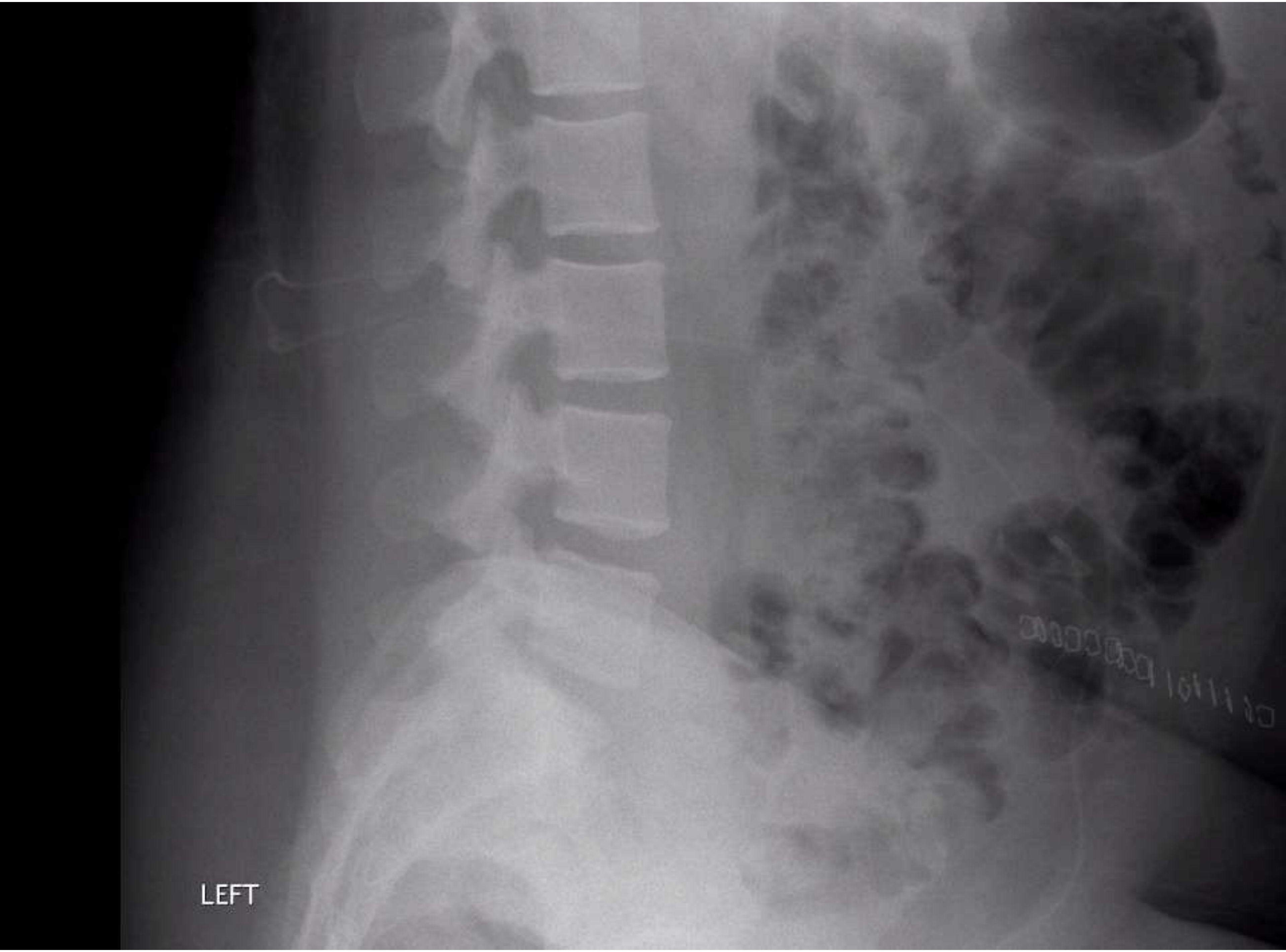
LEFT

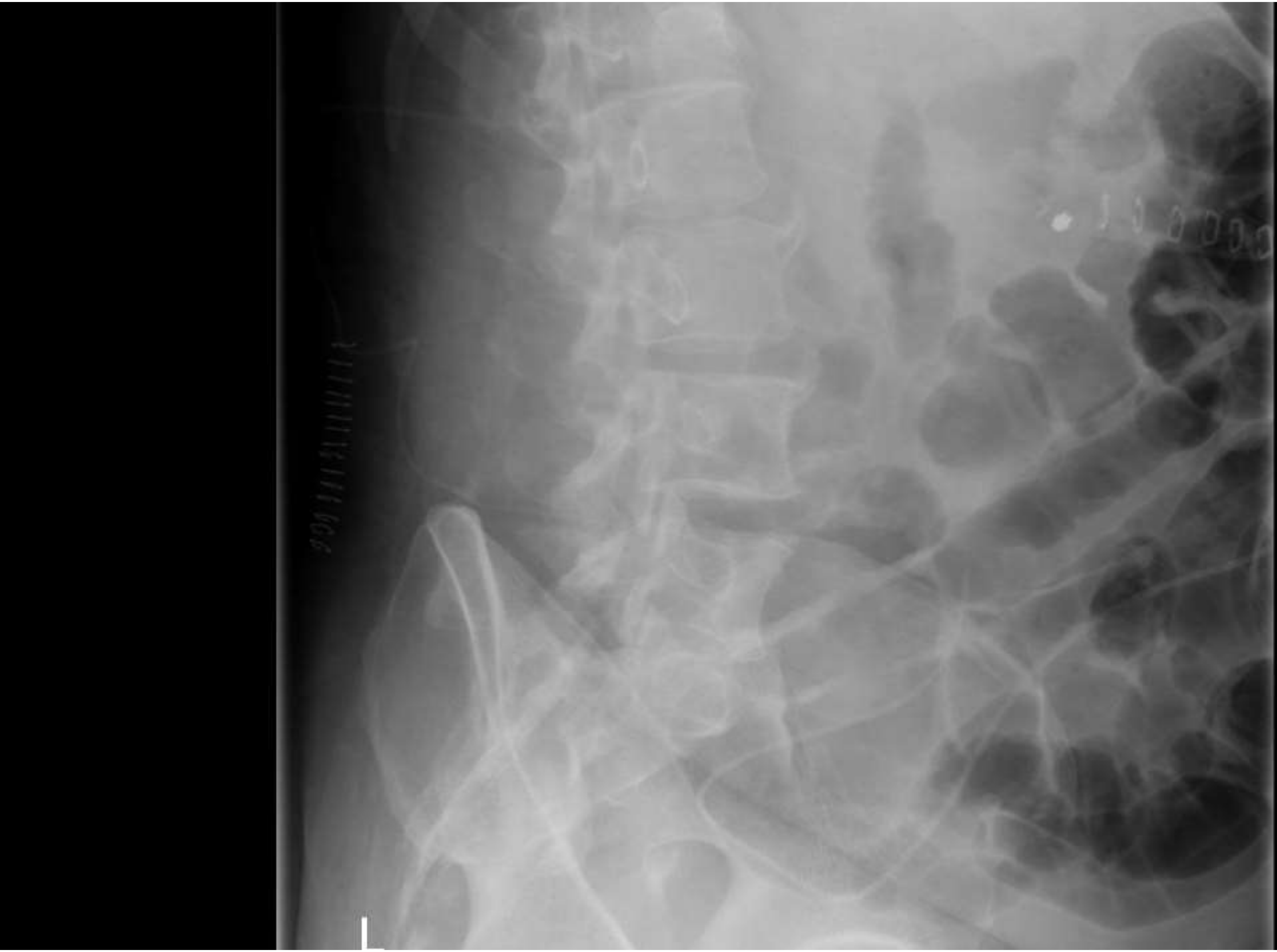

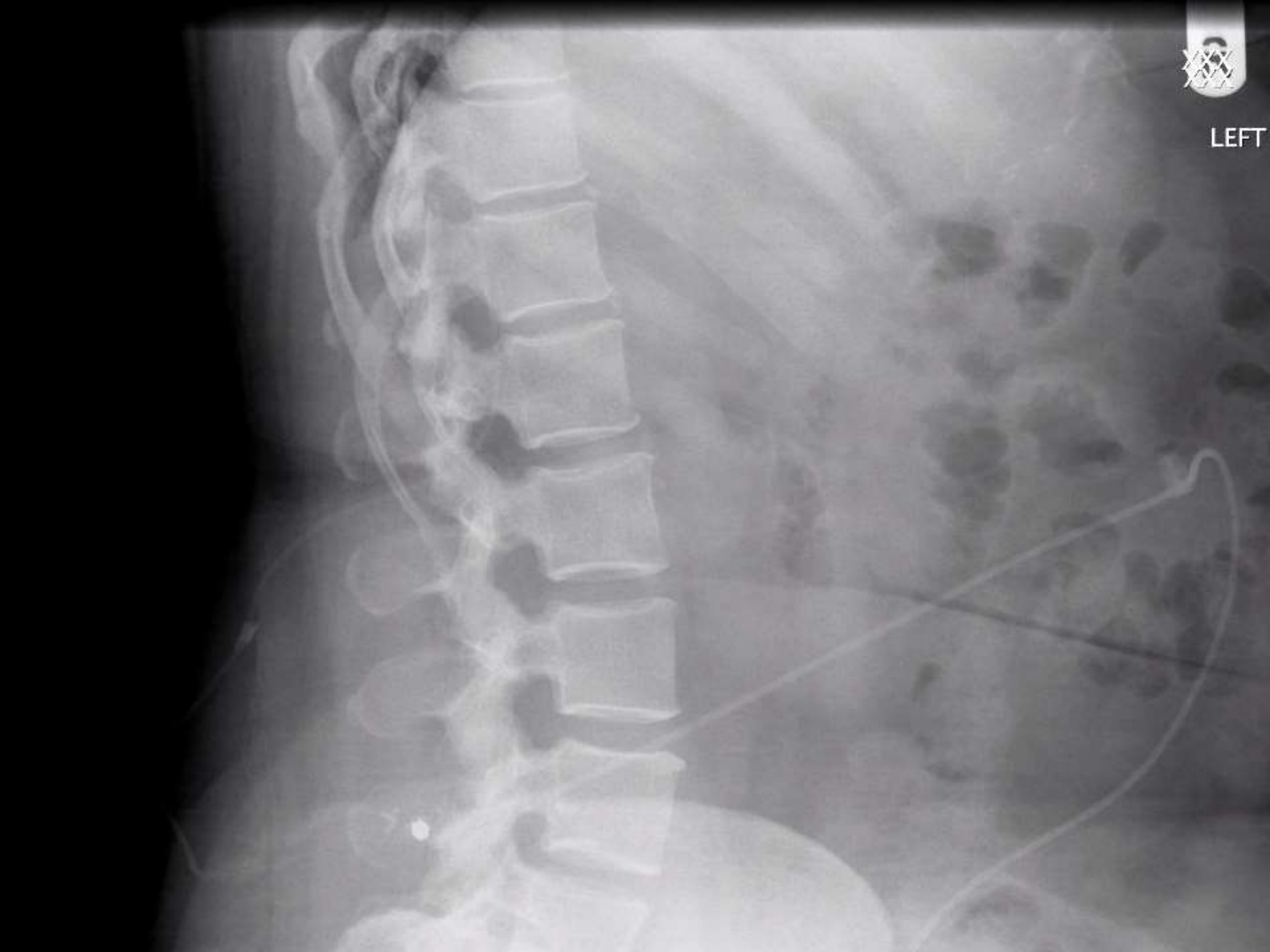
LEFT

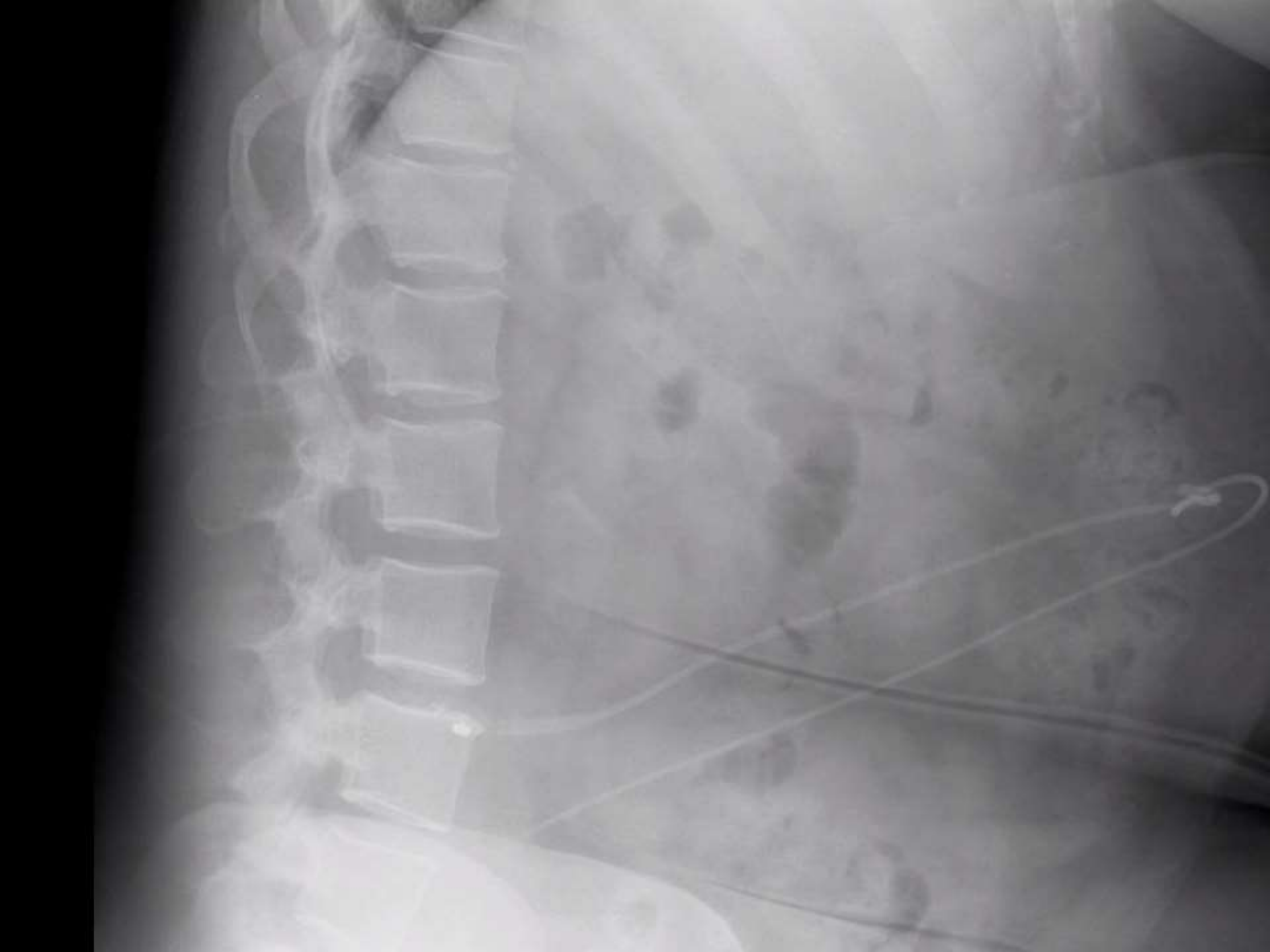

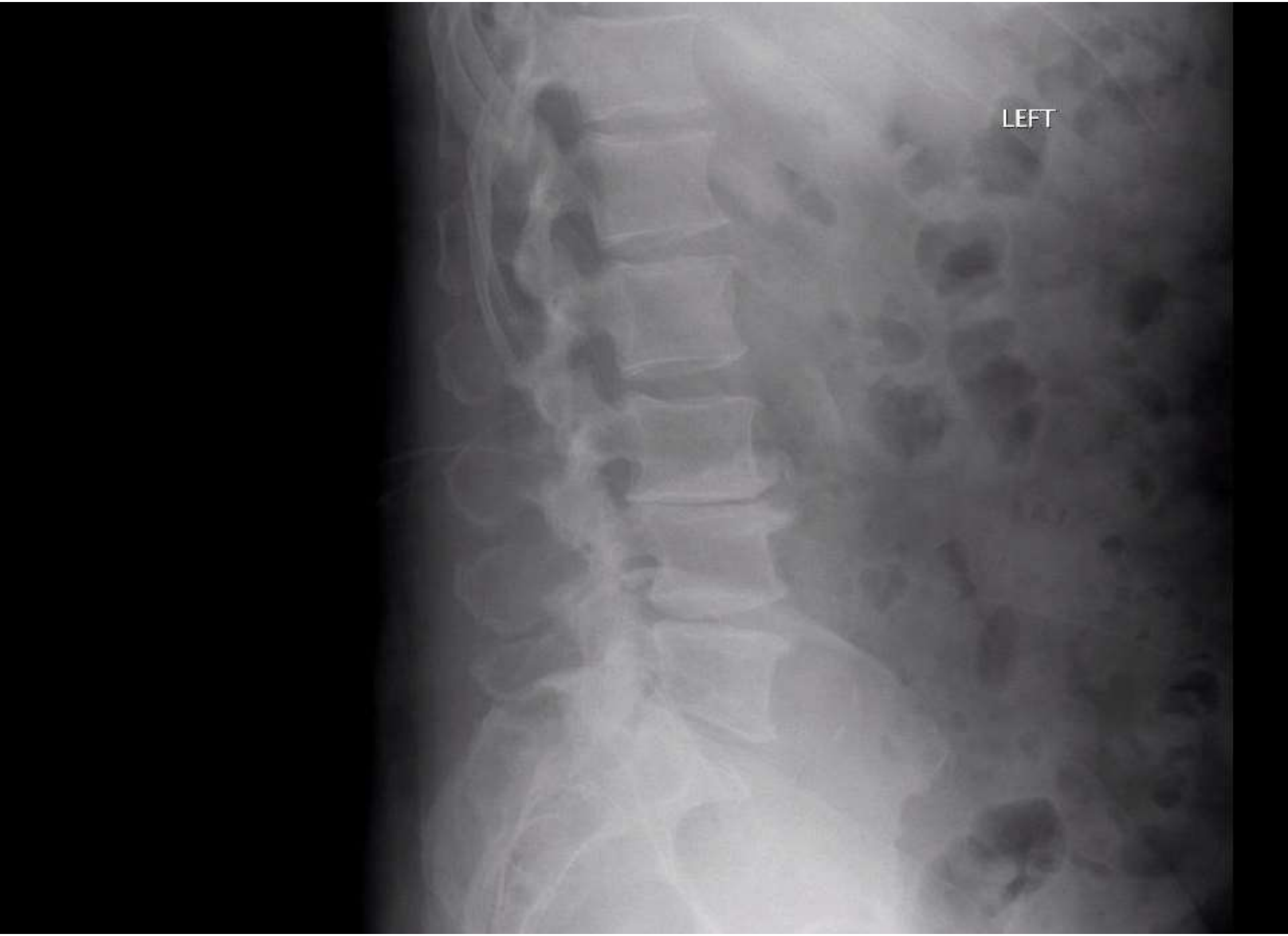
LEFT

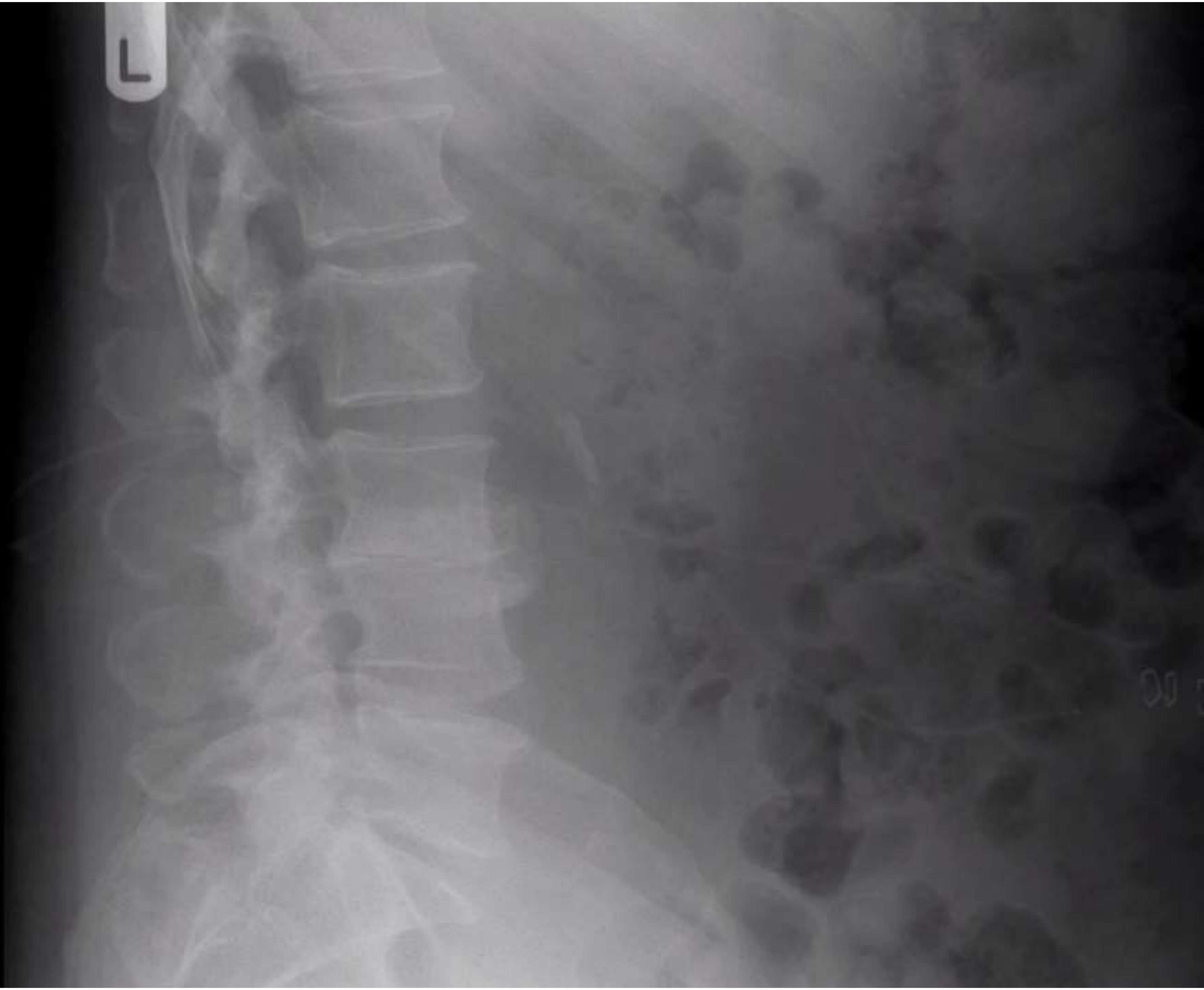
L

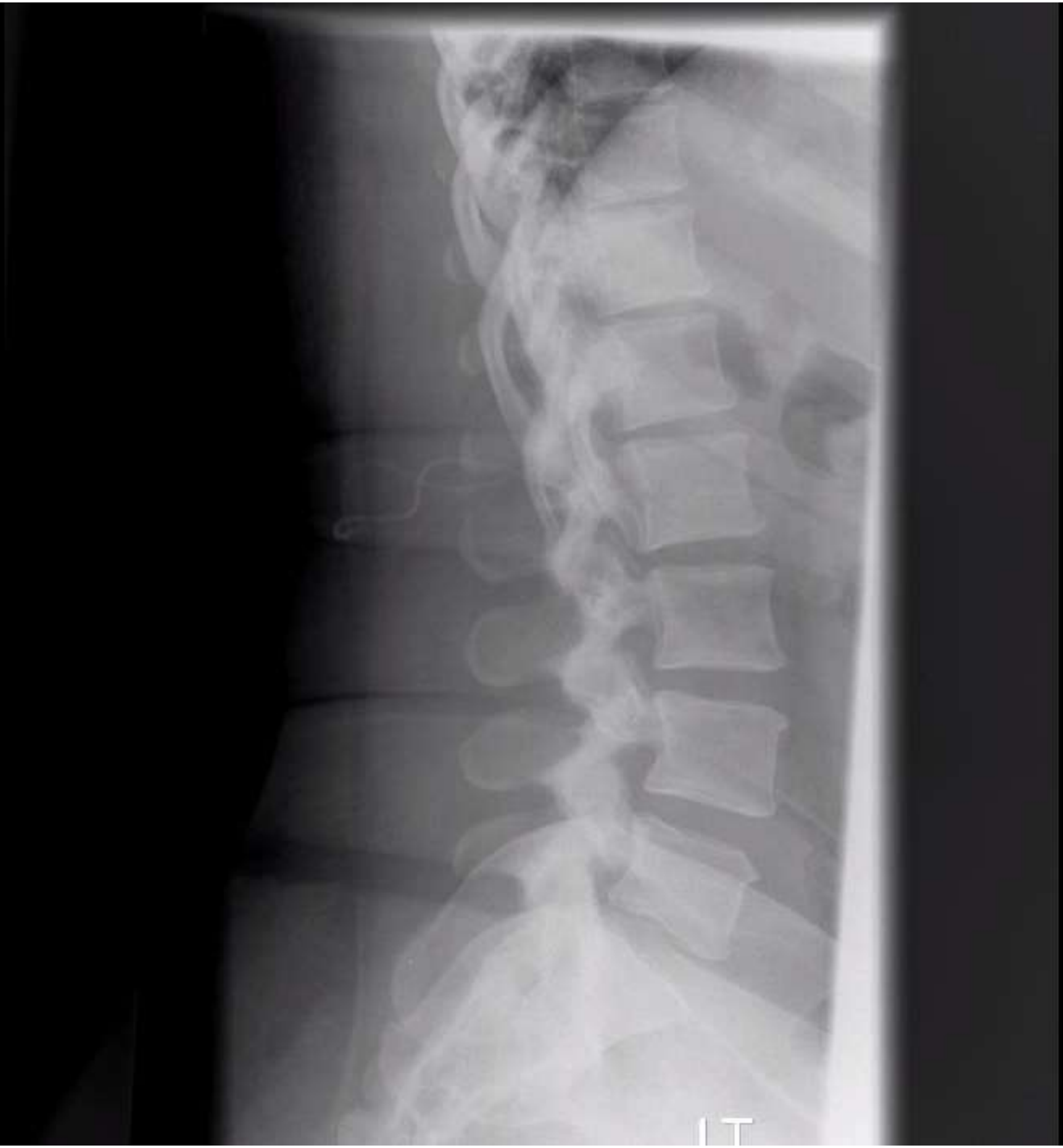

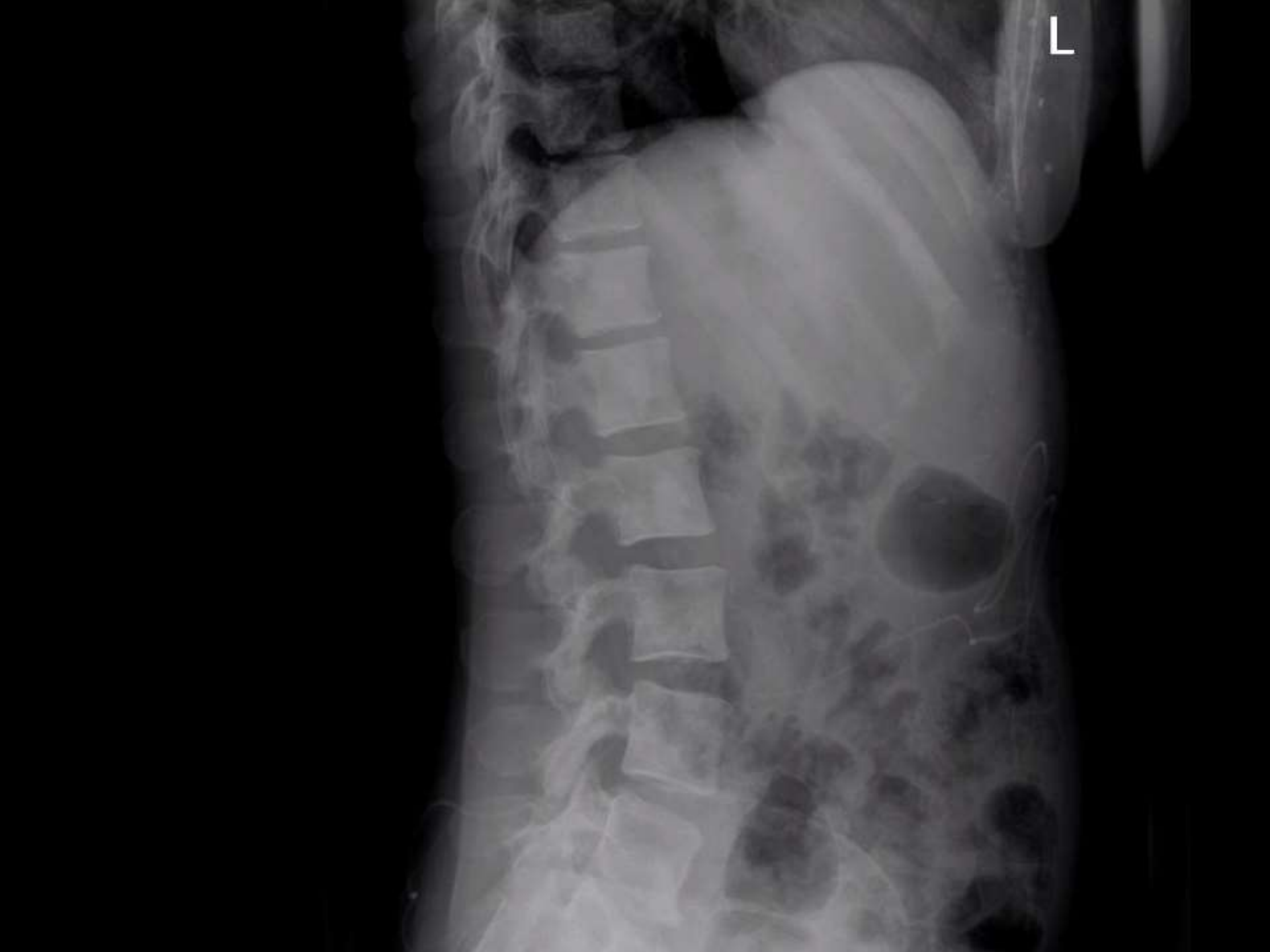
L

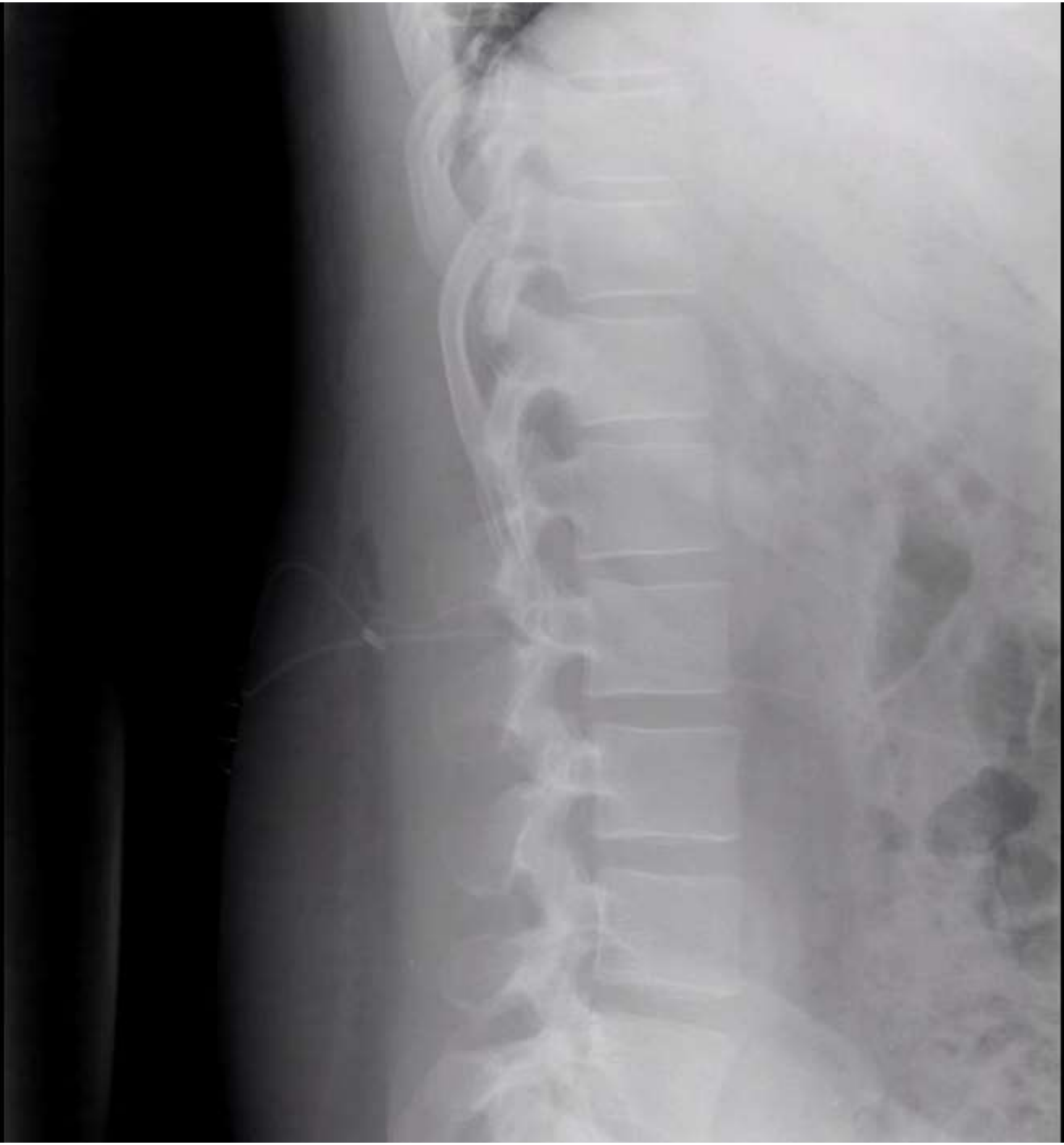

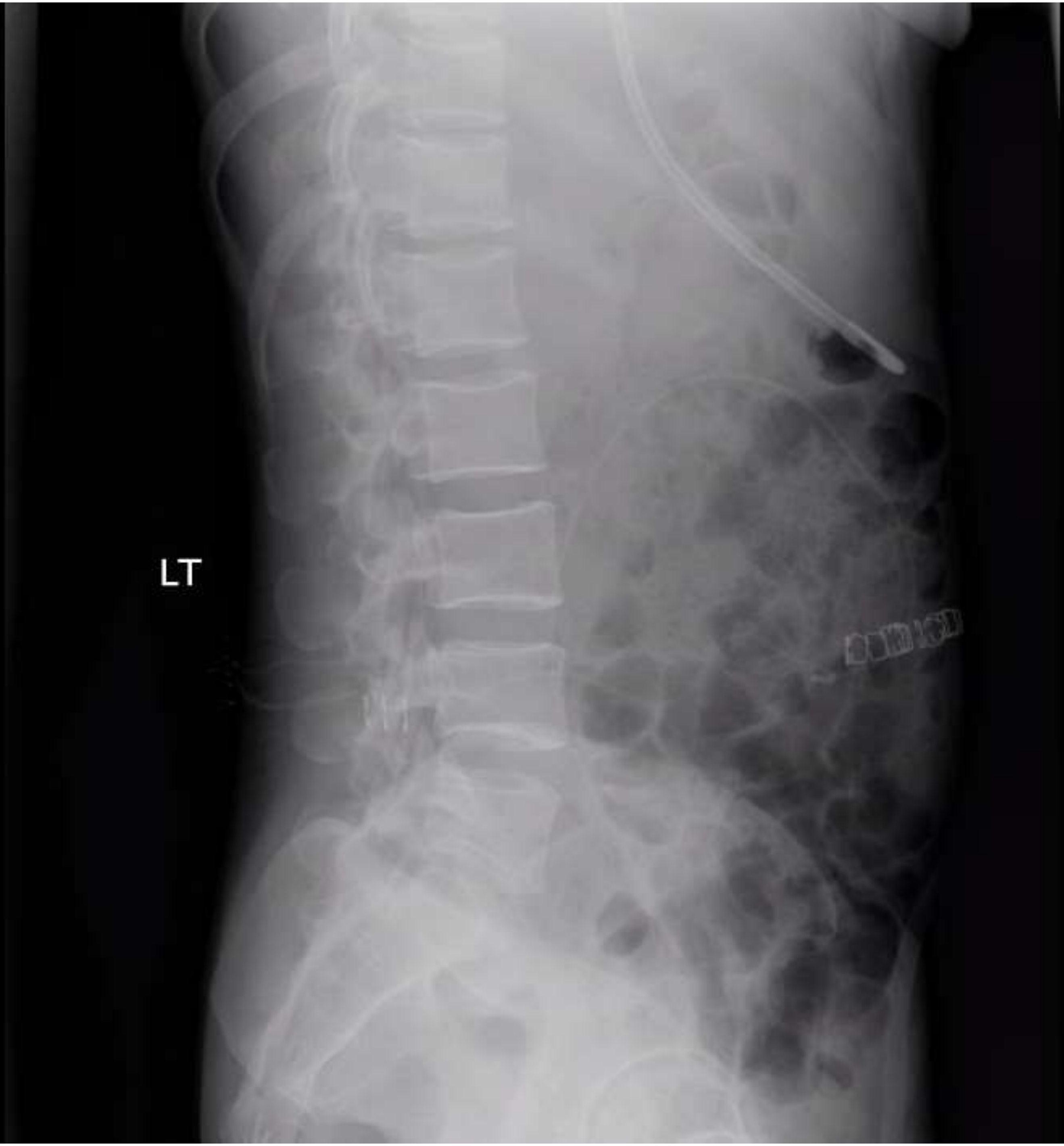
LT

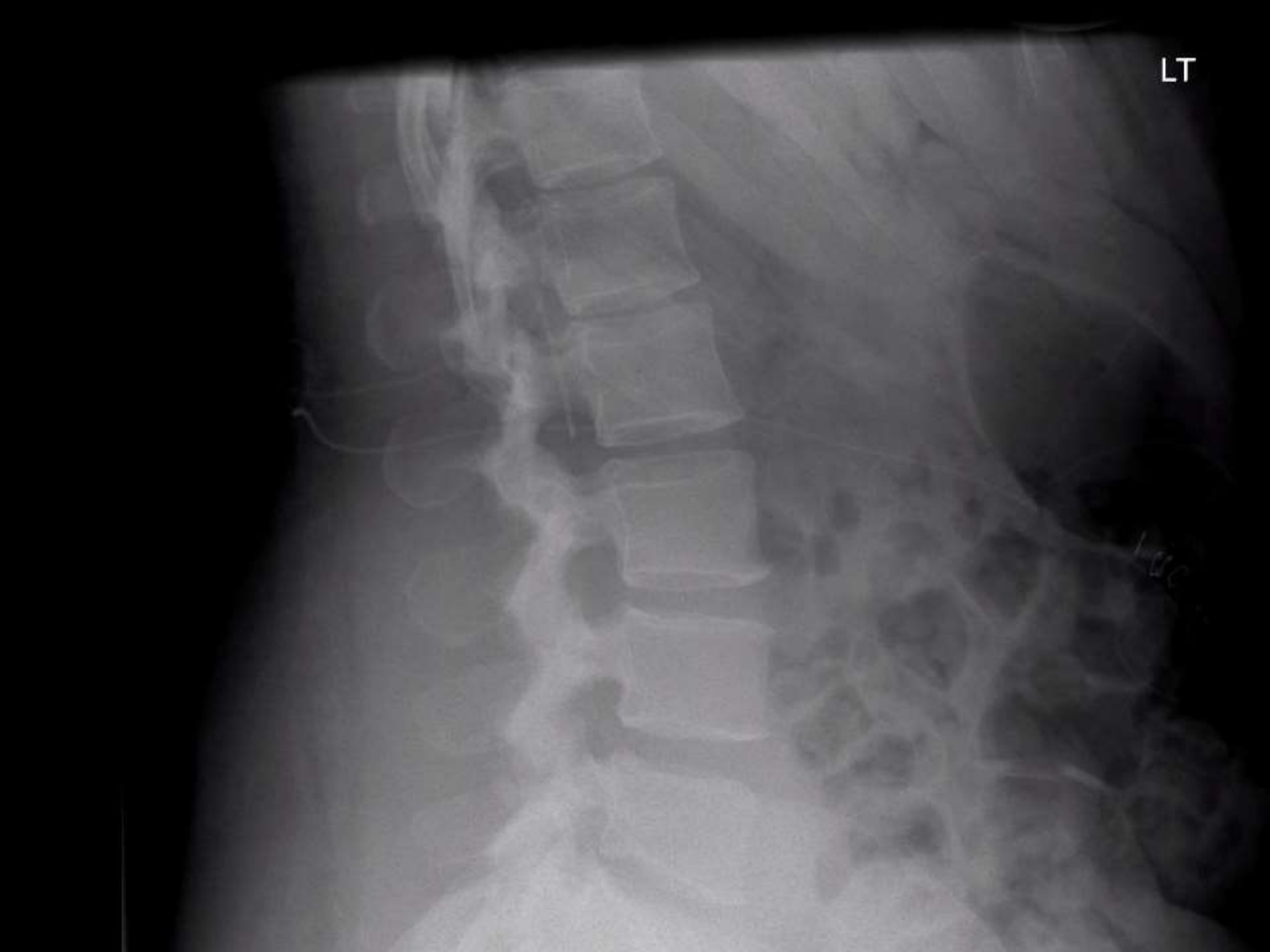
LT

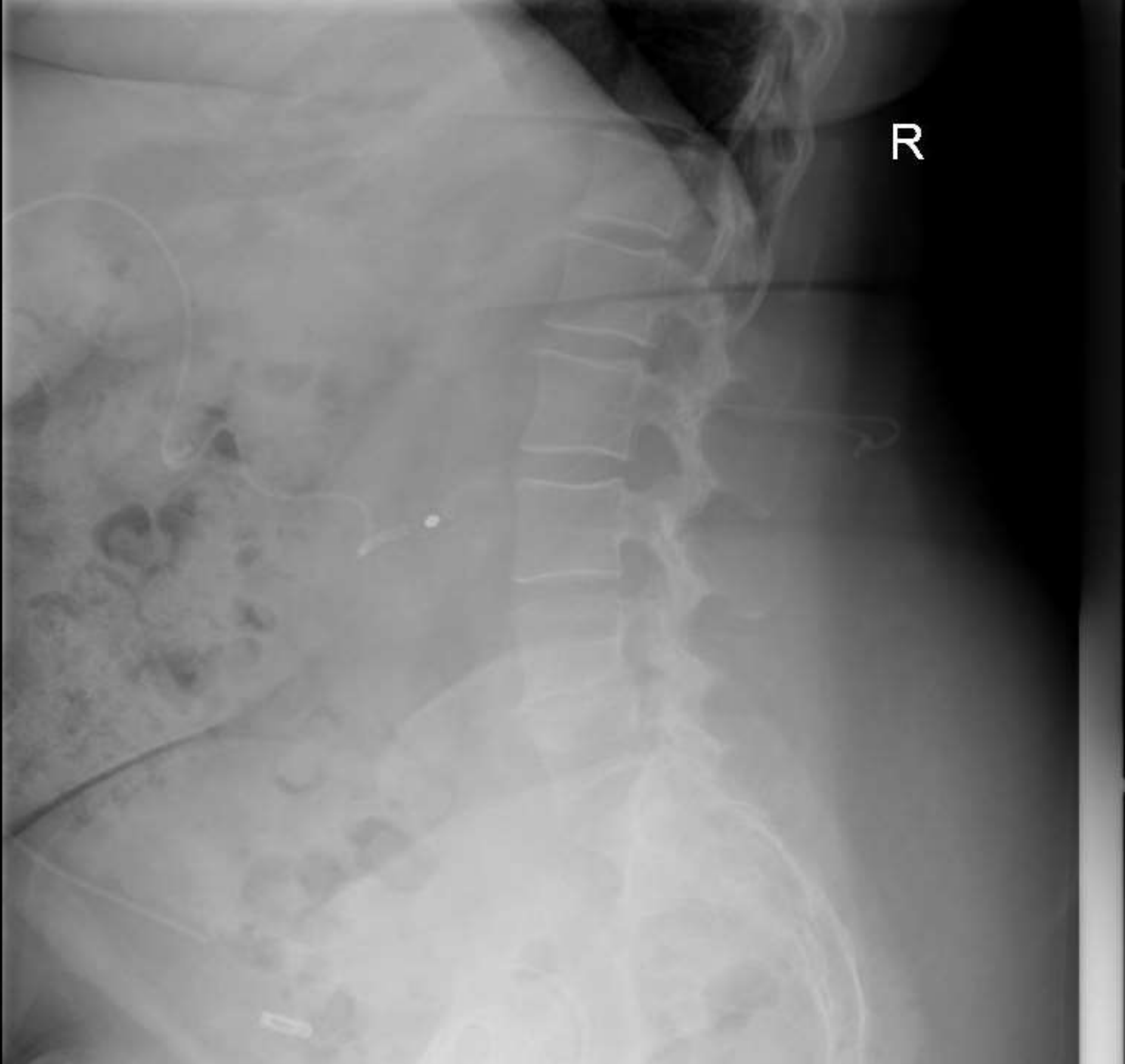
R

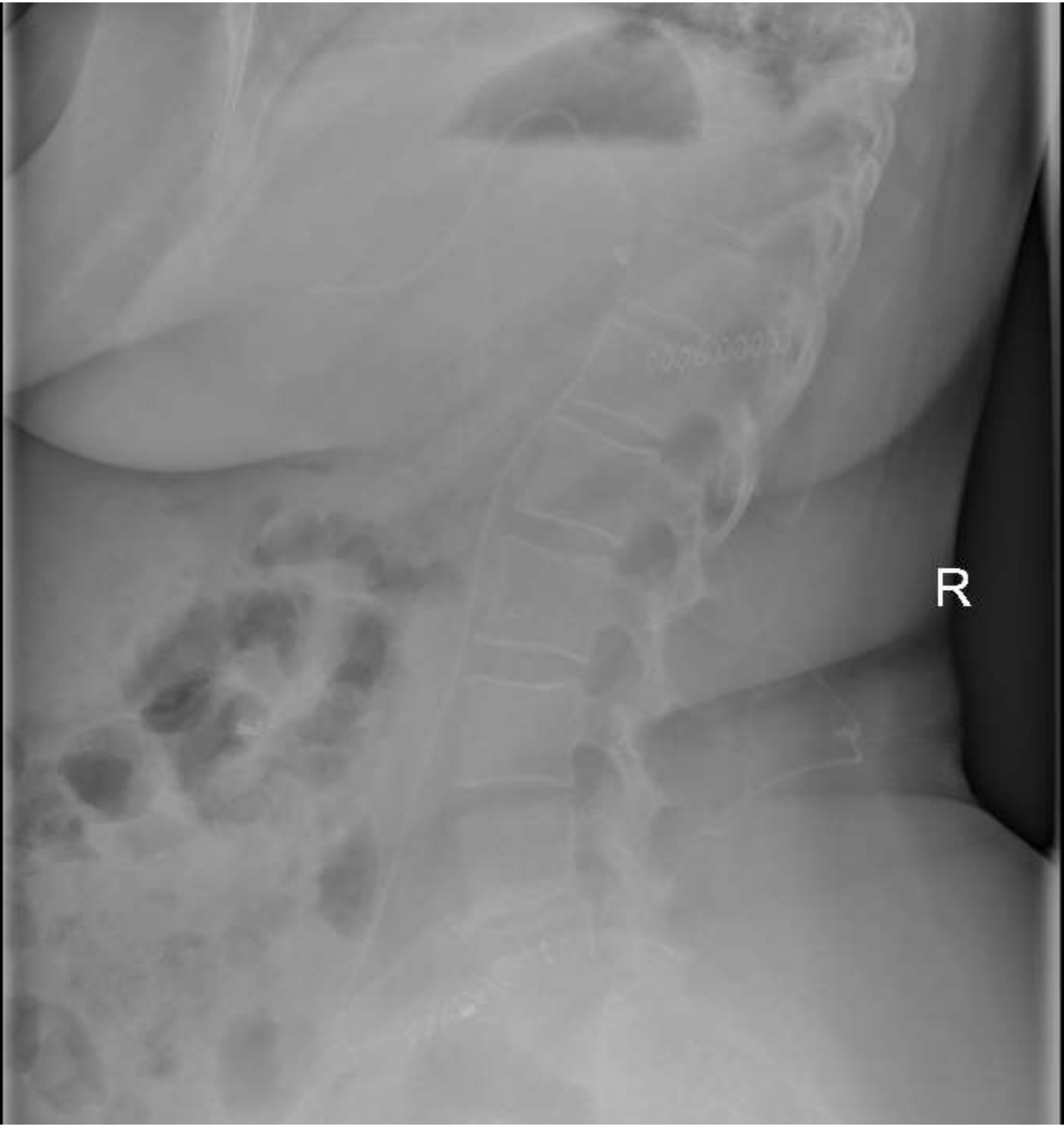
R

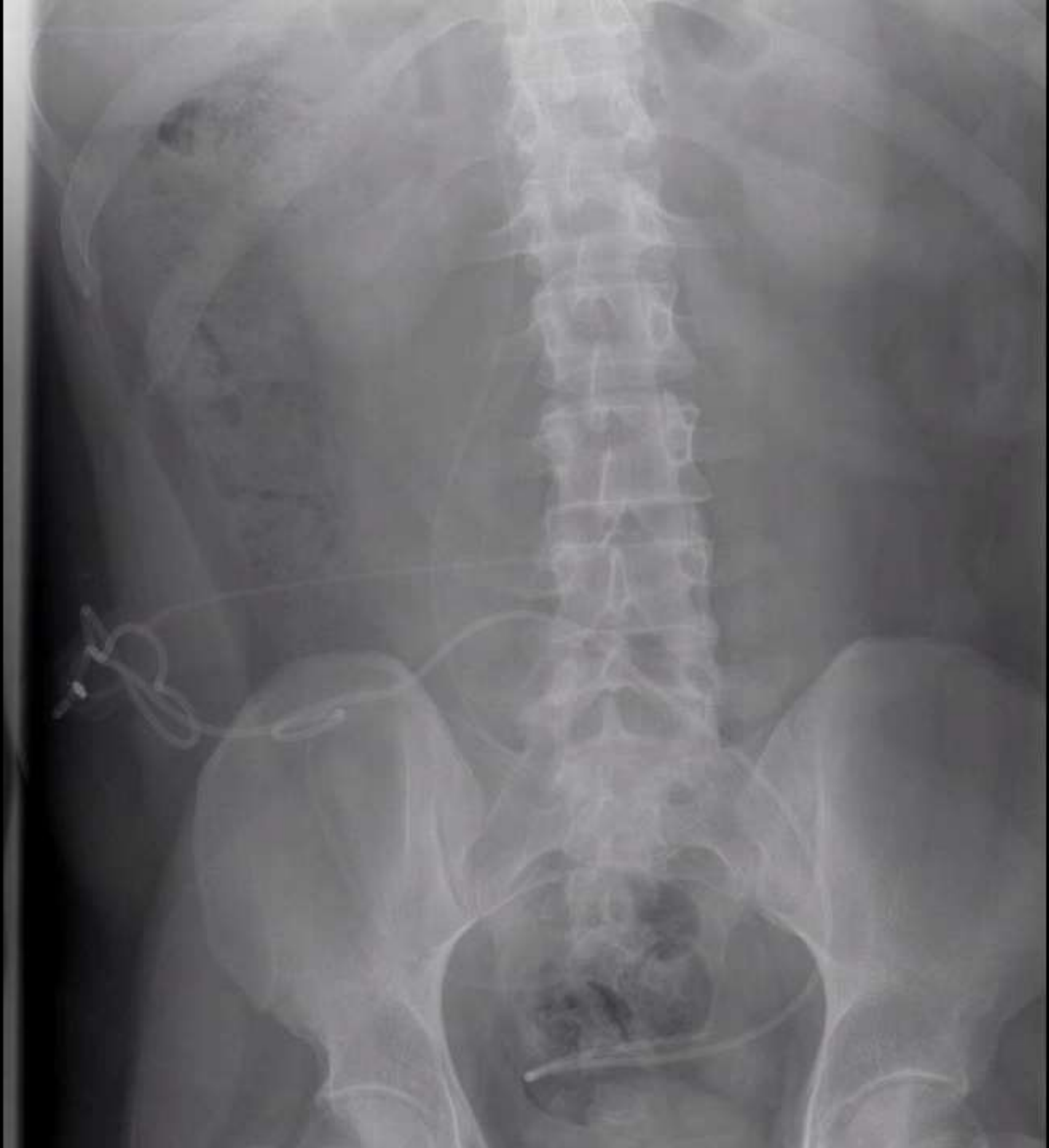

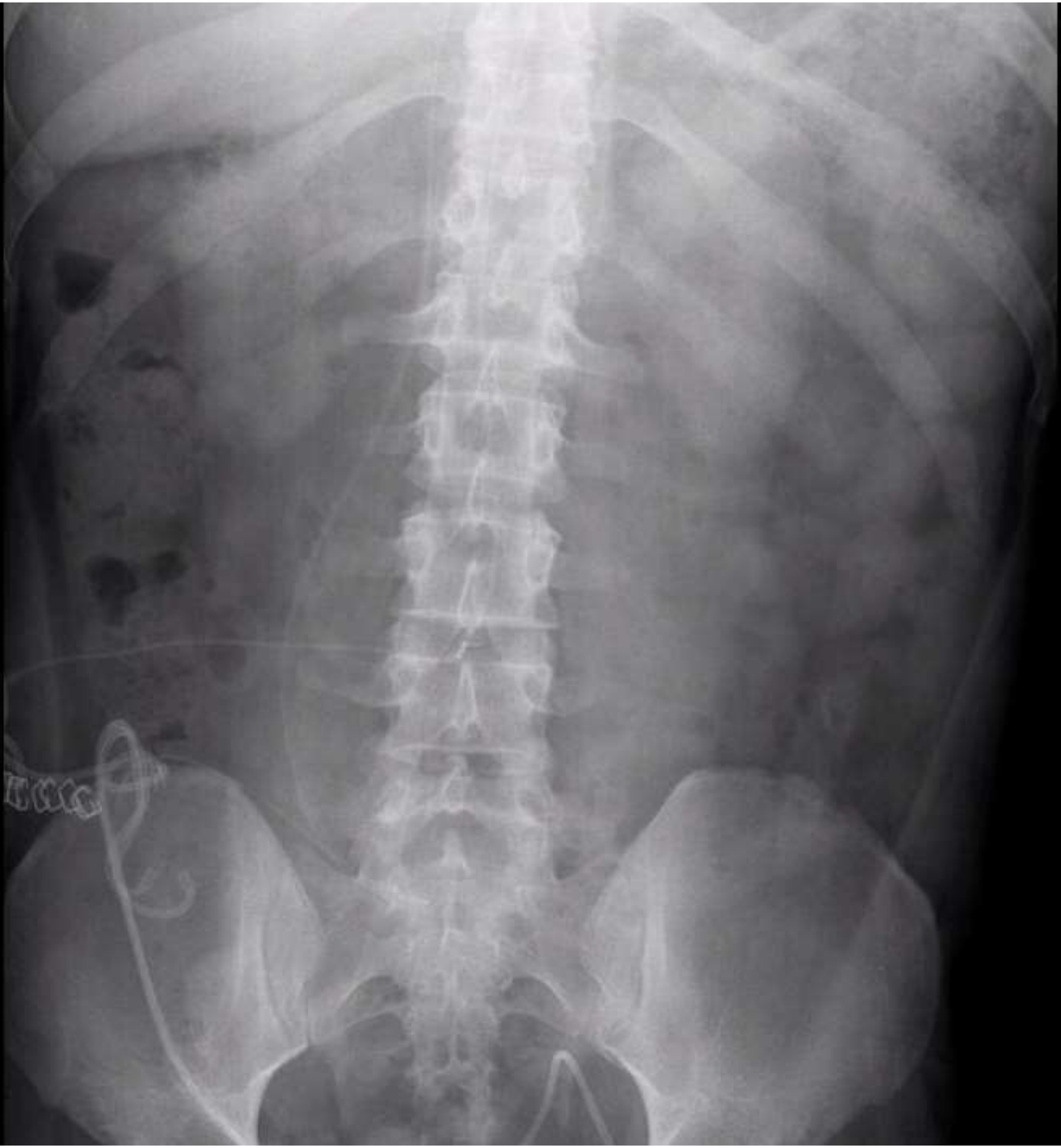

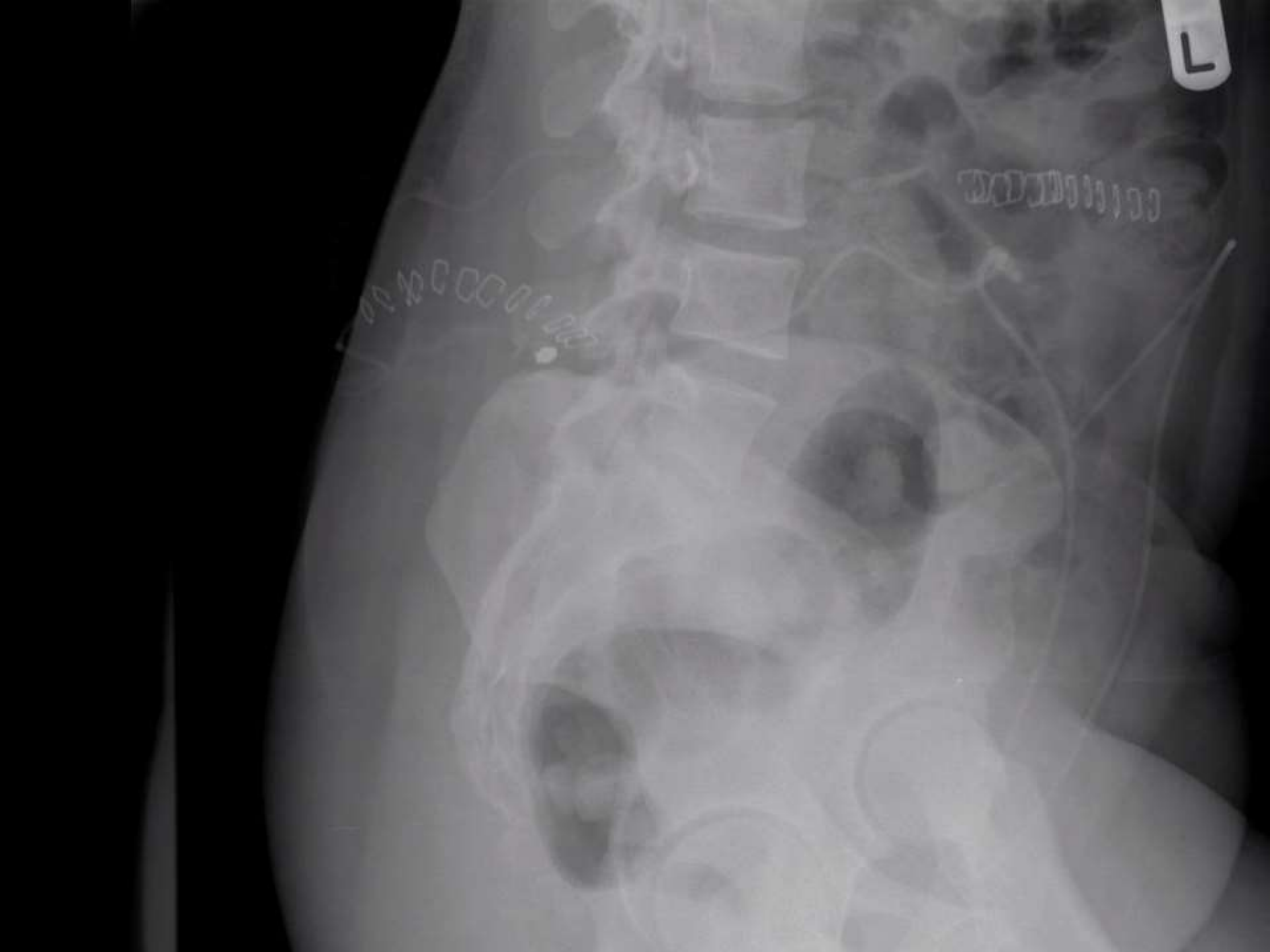
L

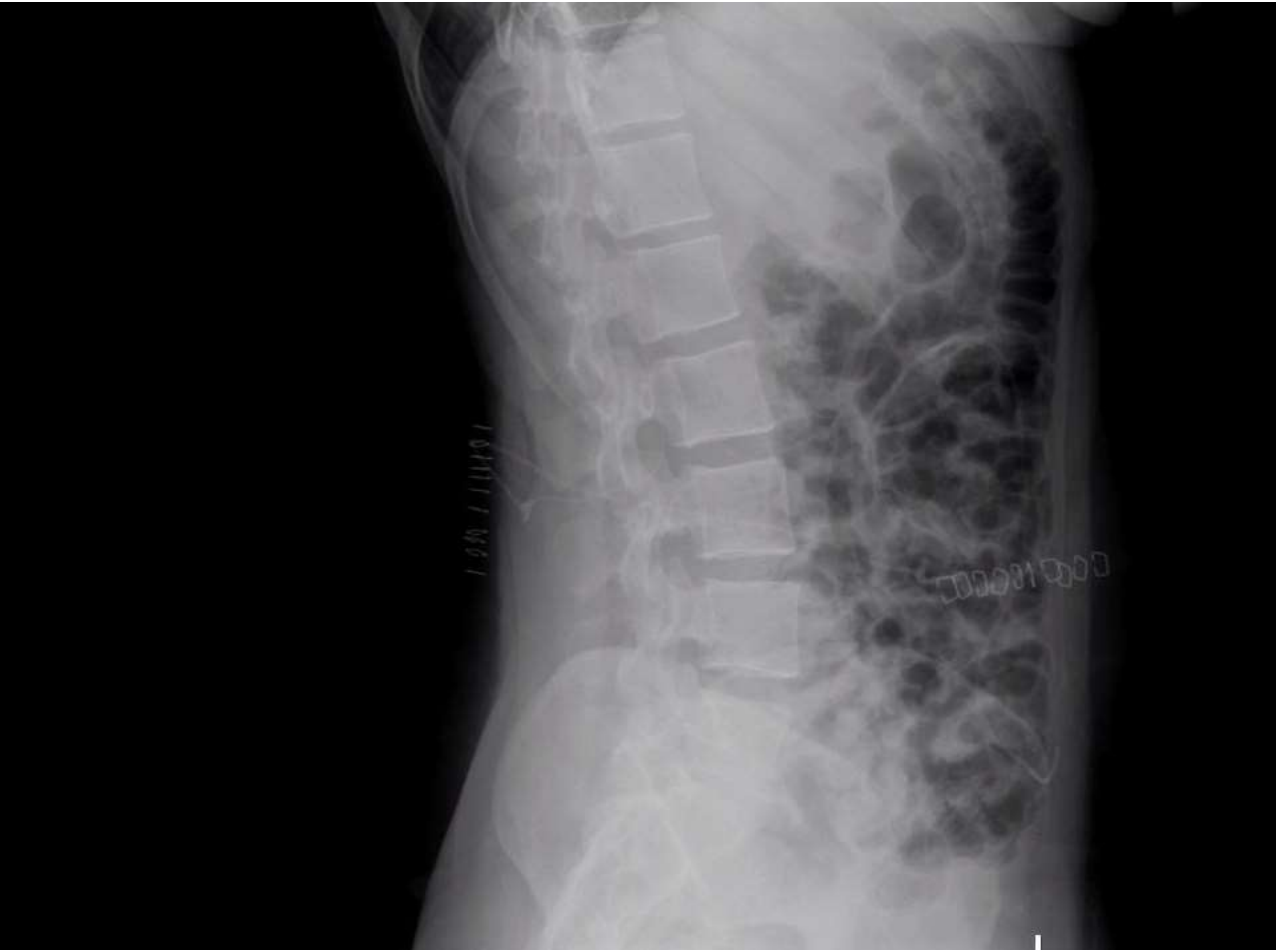

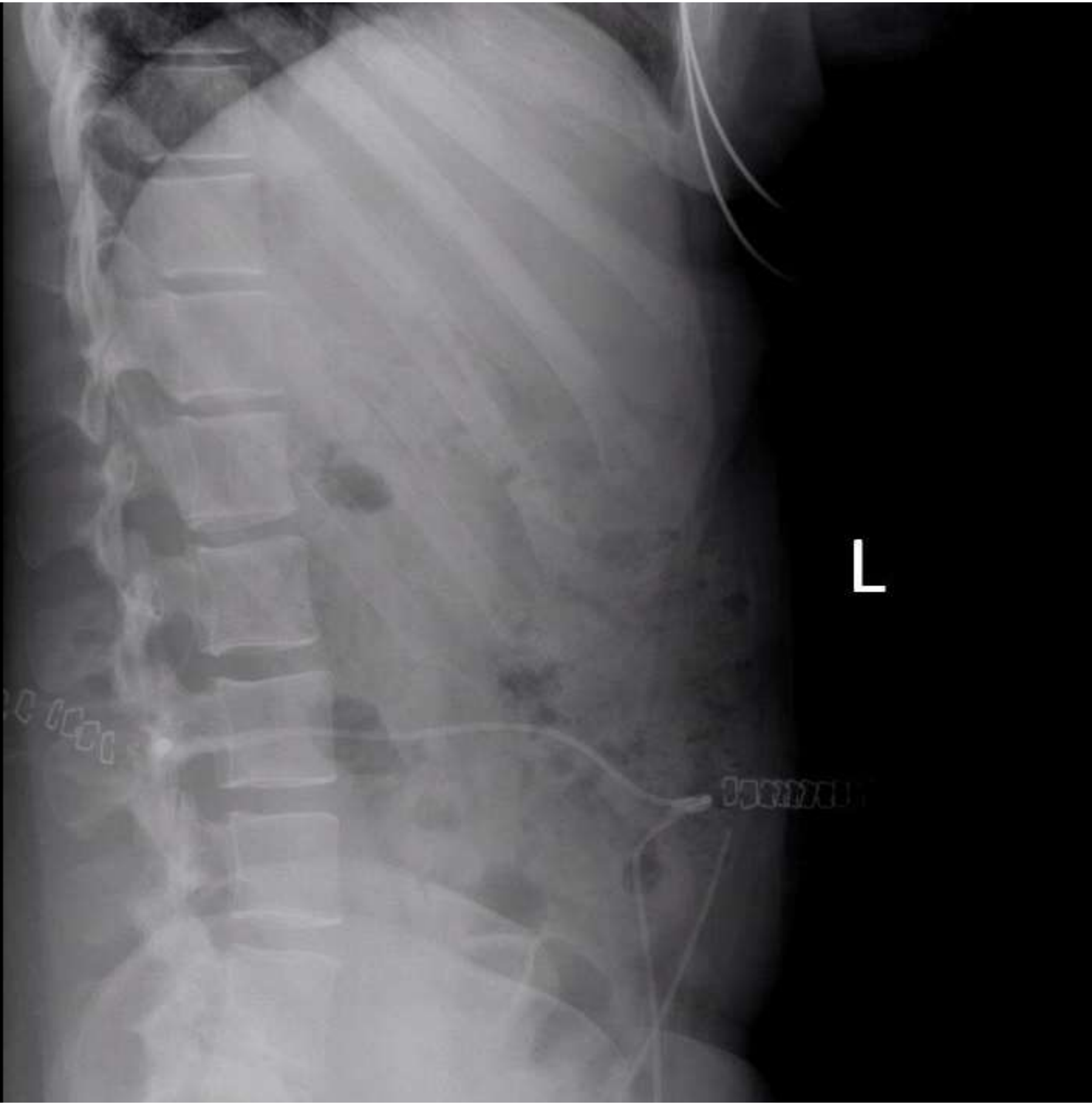
L

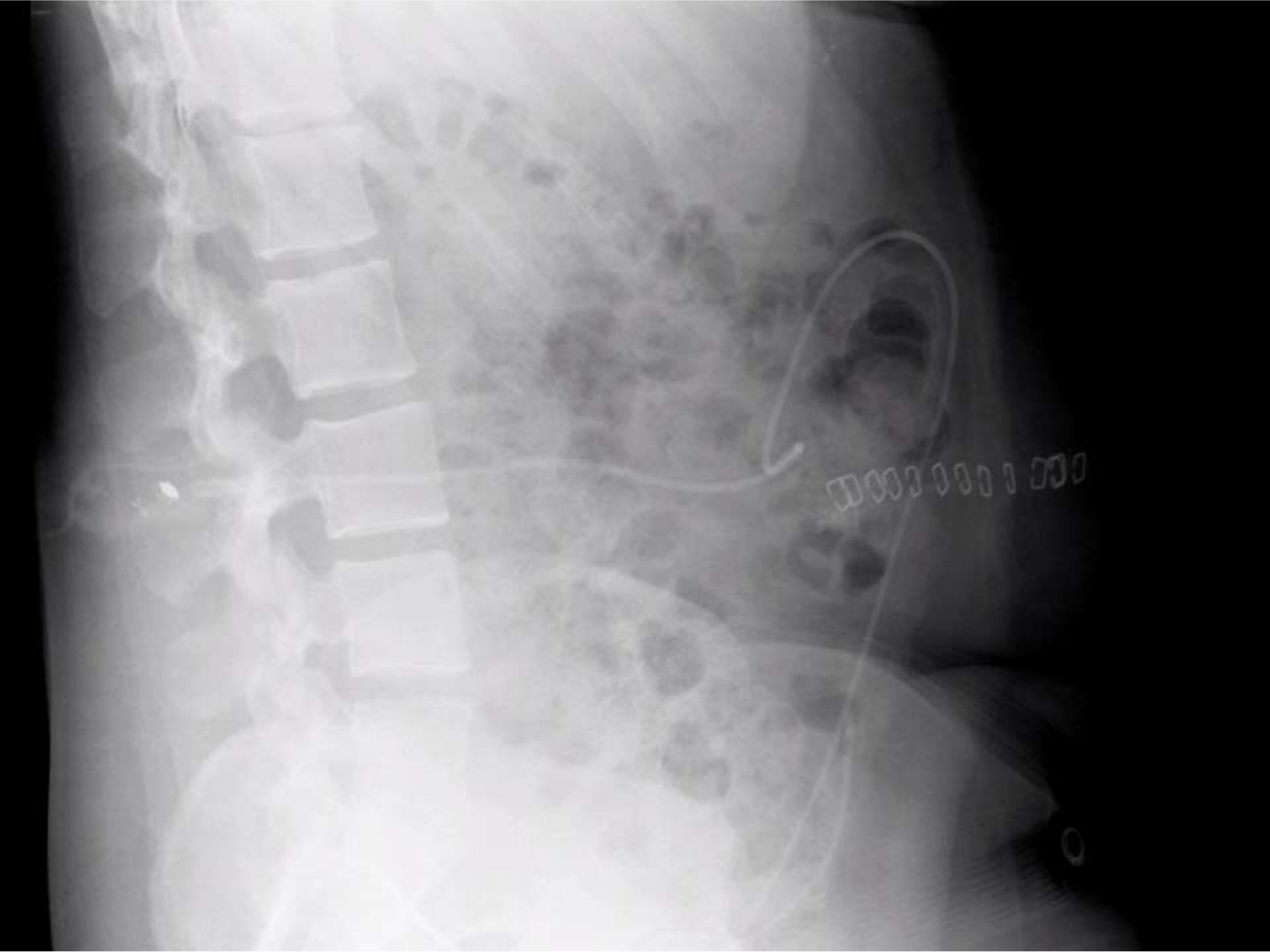

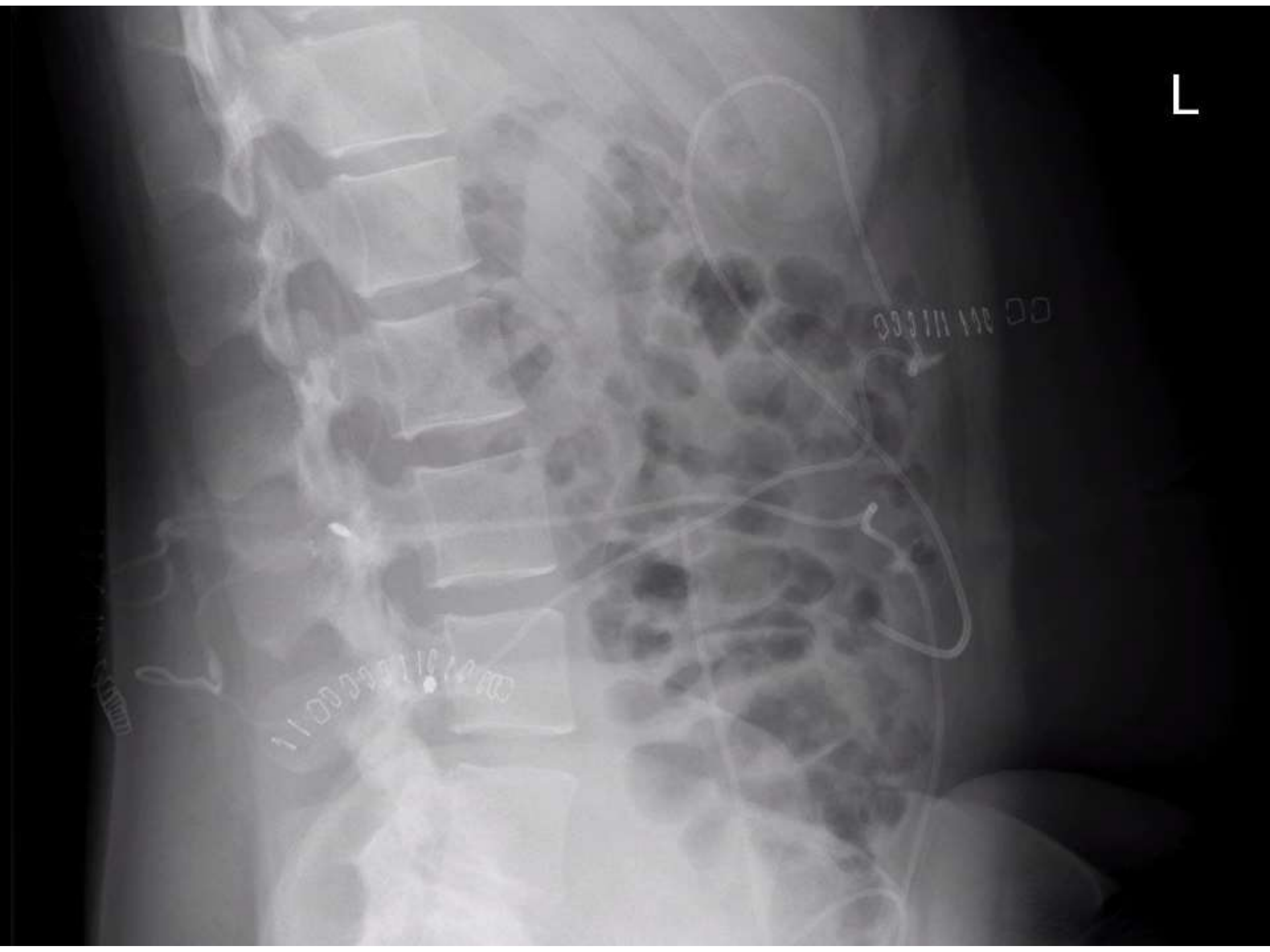
L

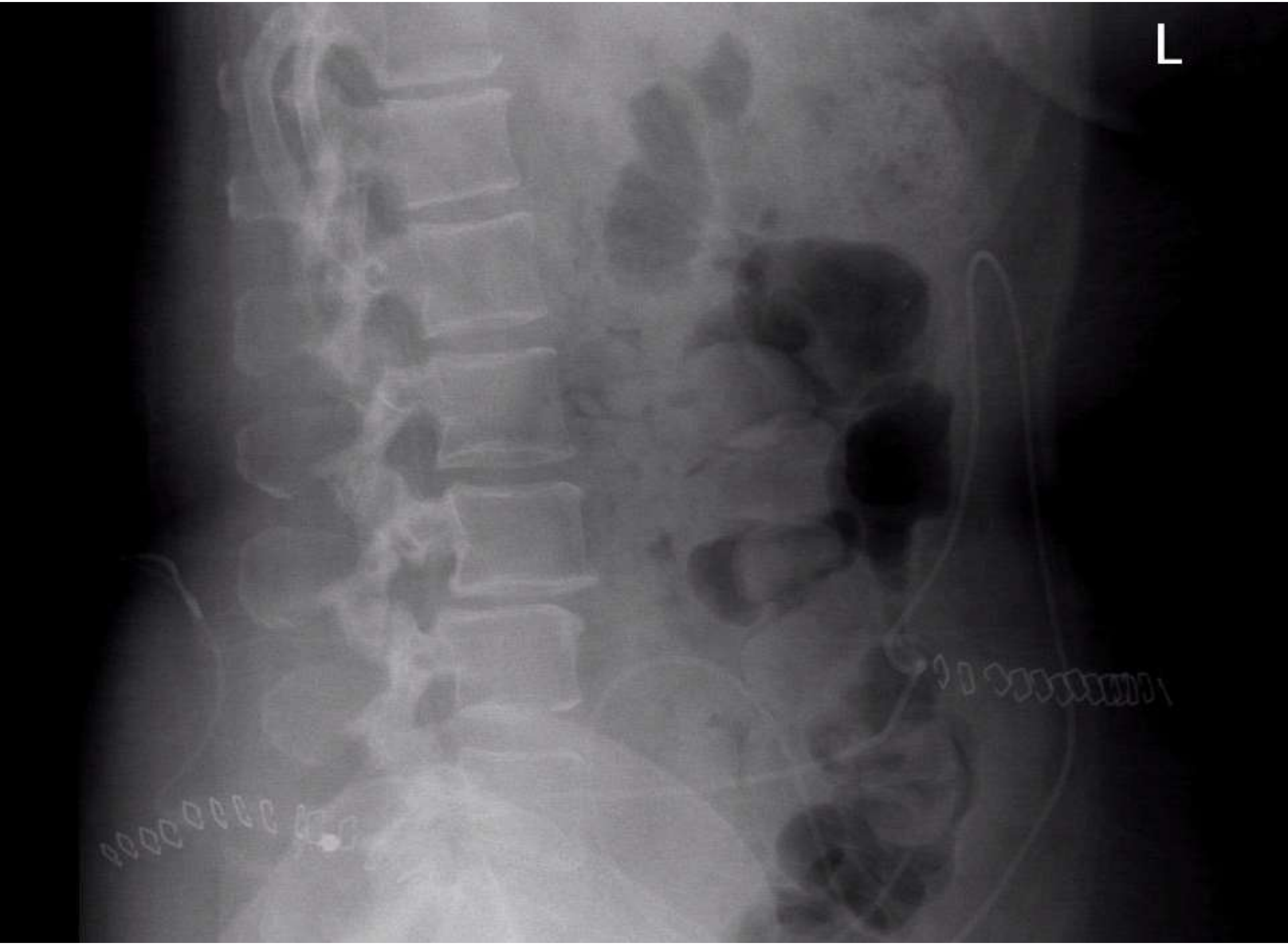
L

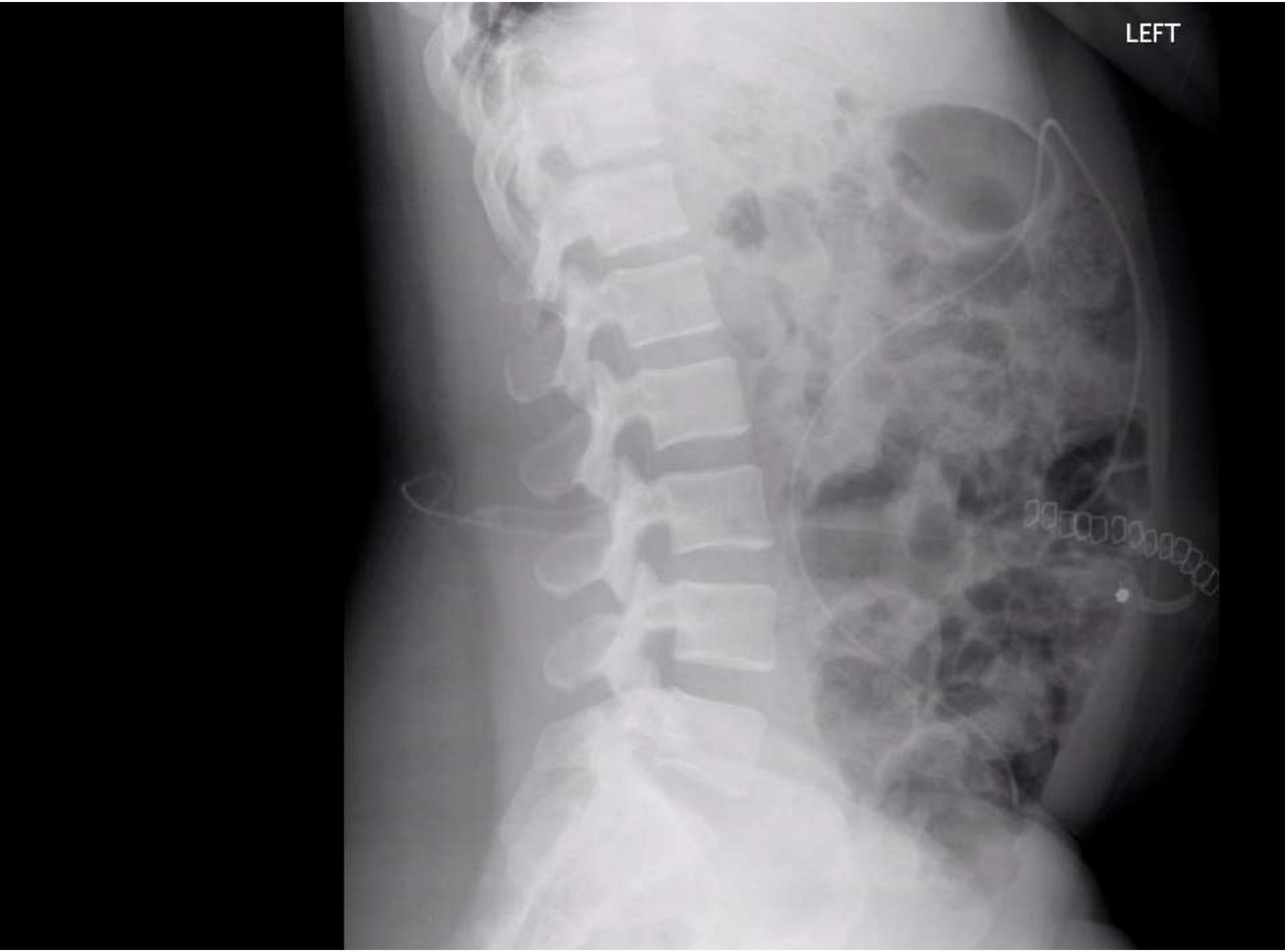
LEFT

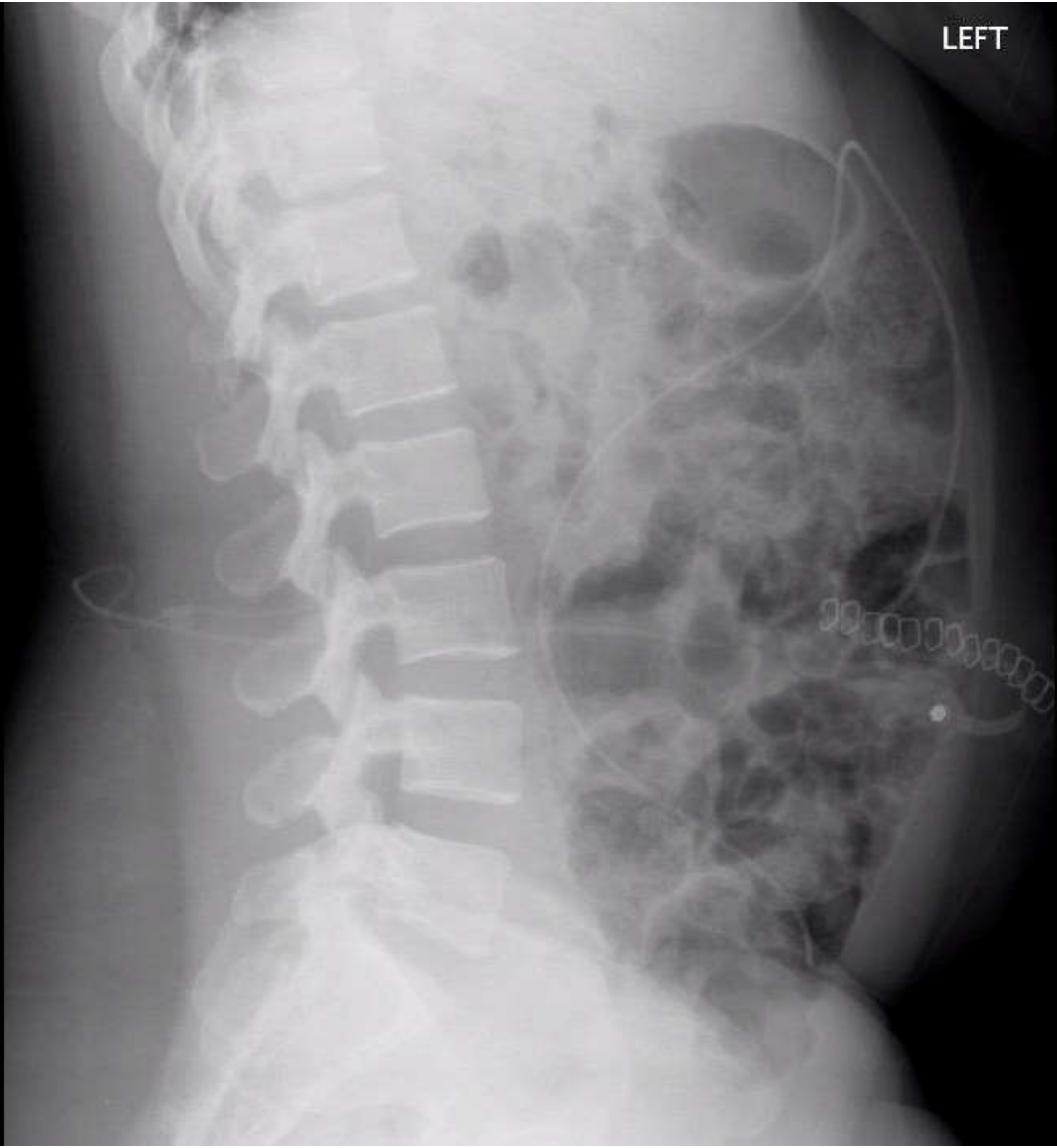
LEFT

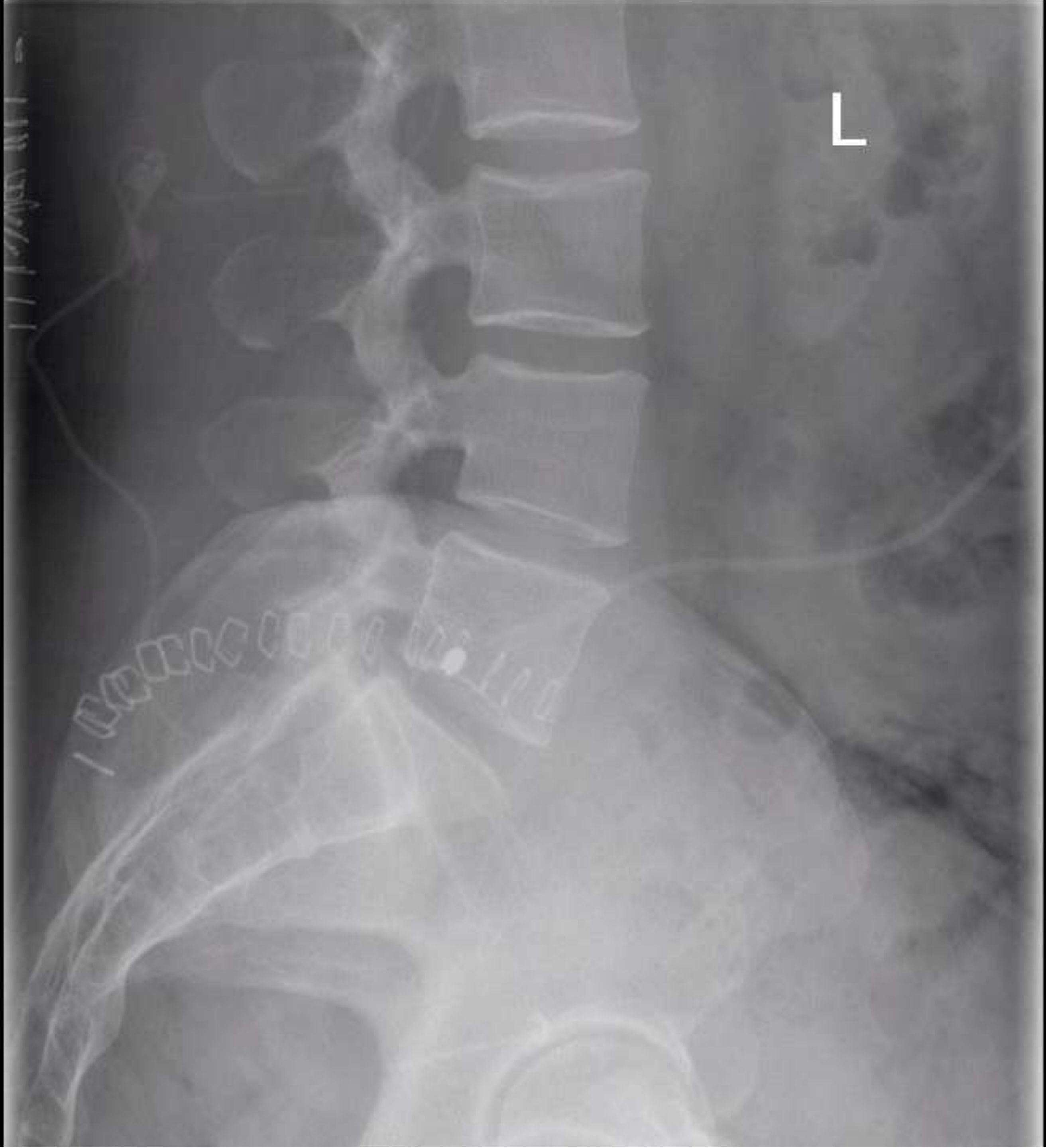
L

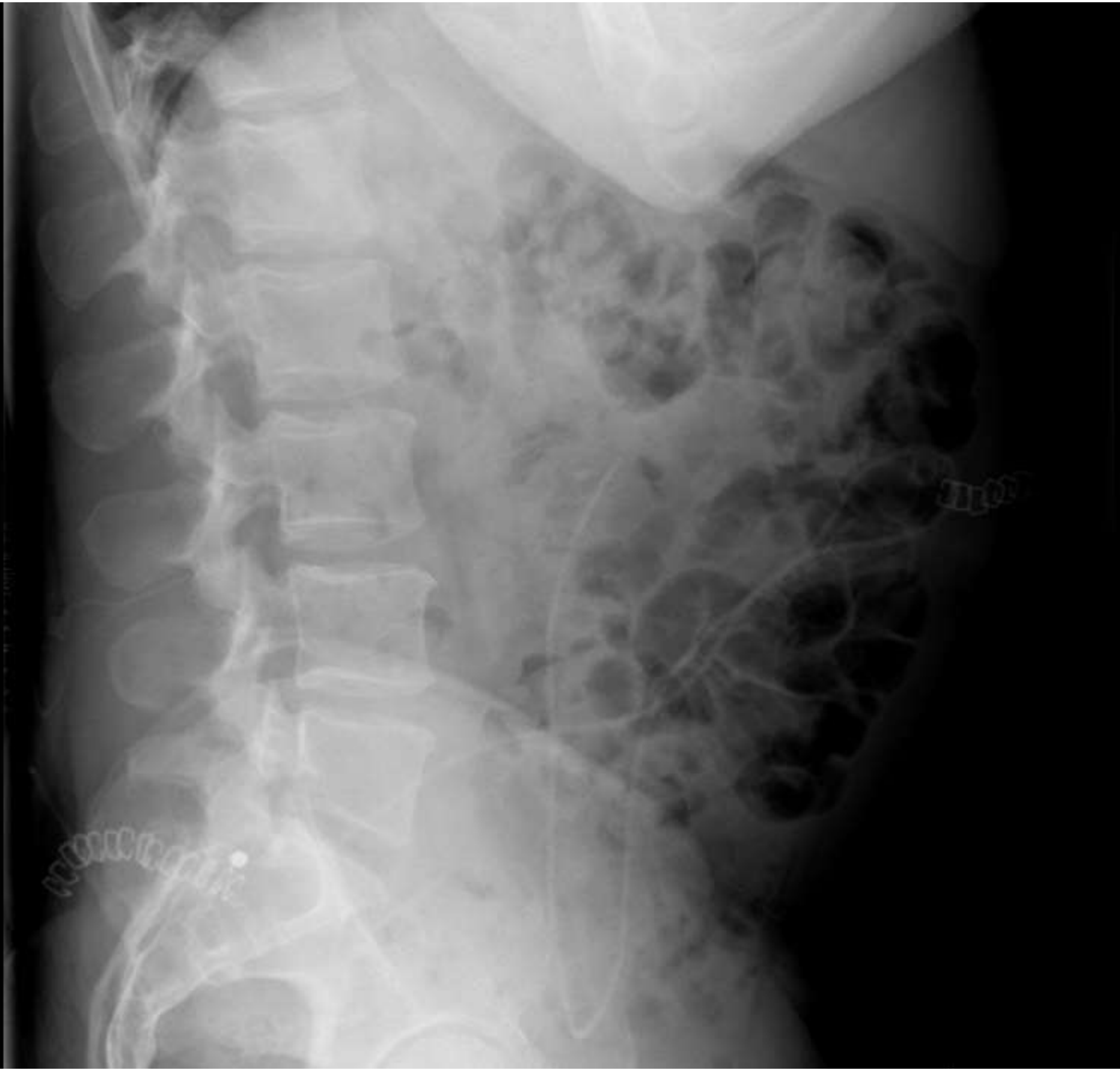

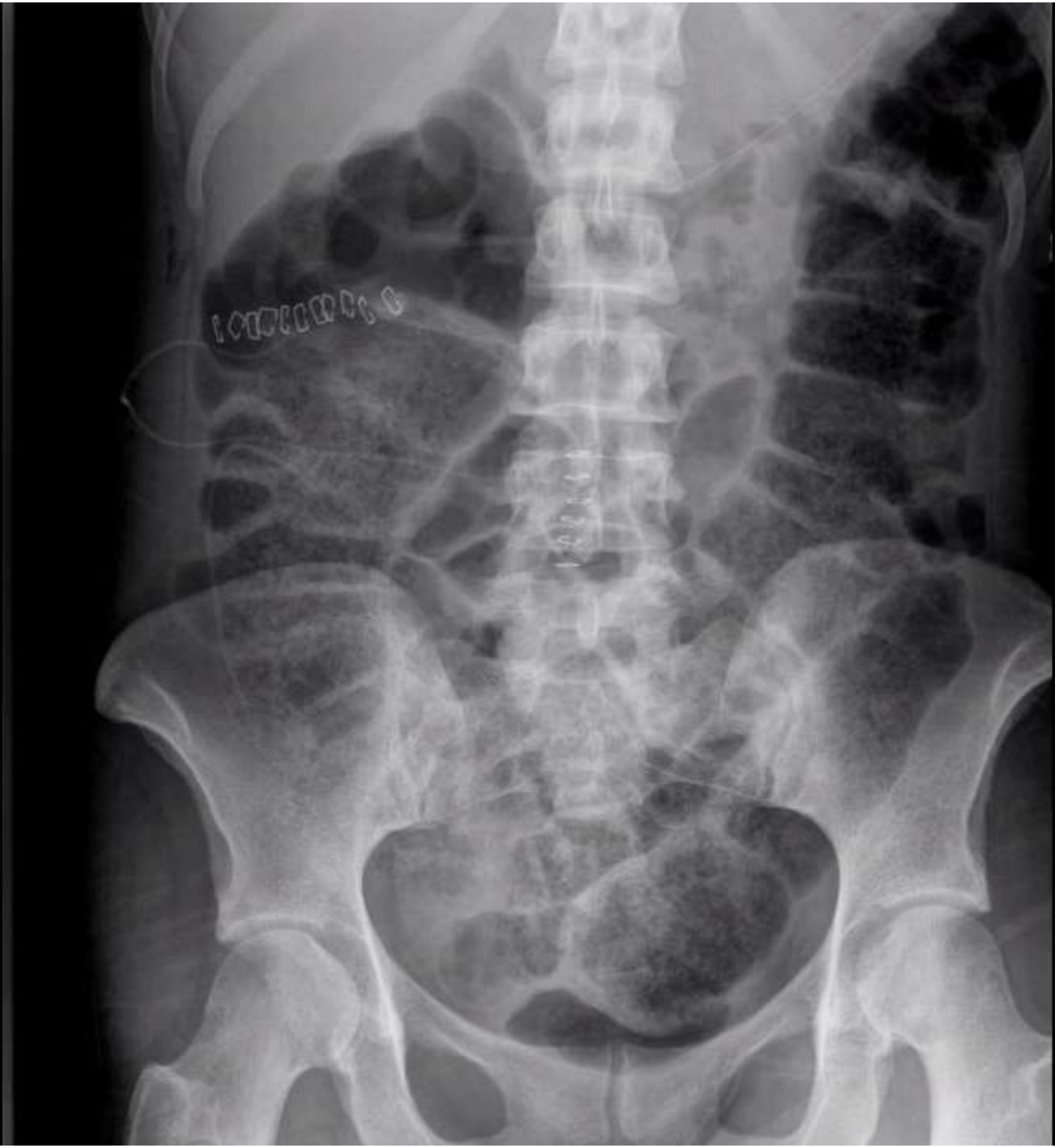

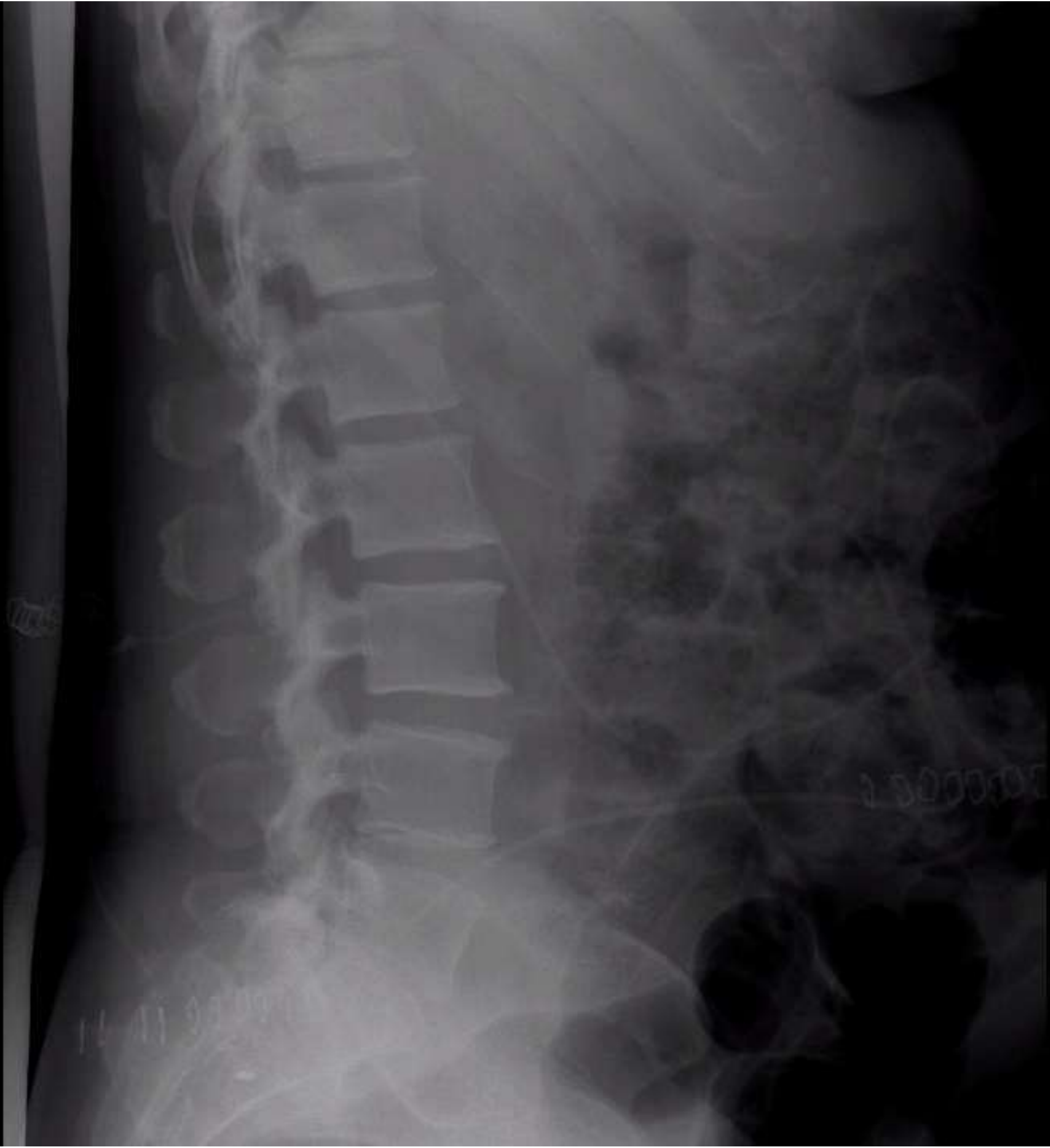

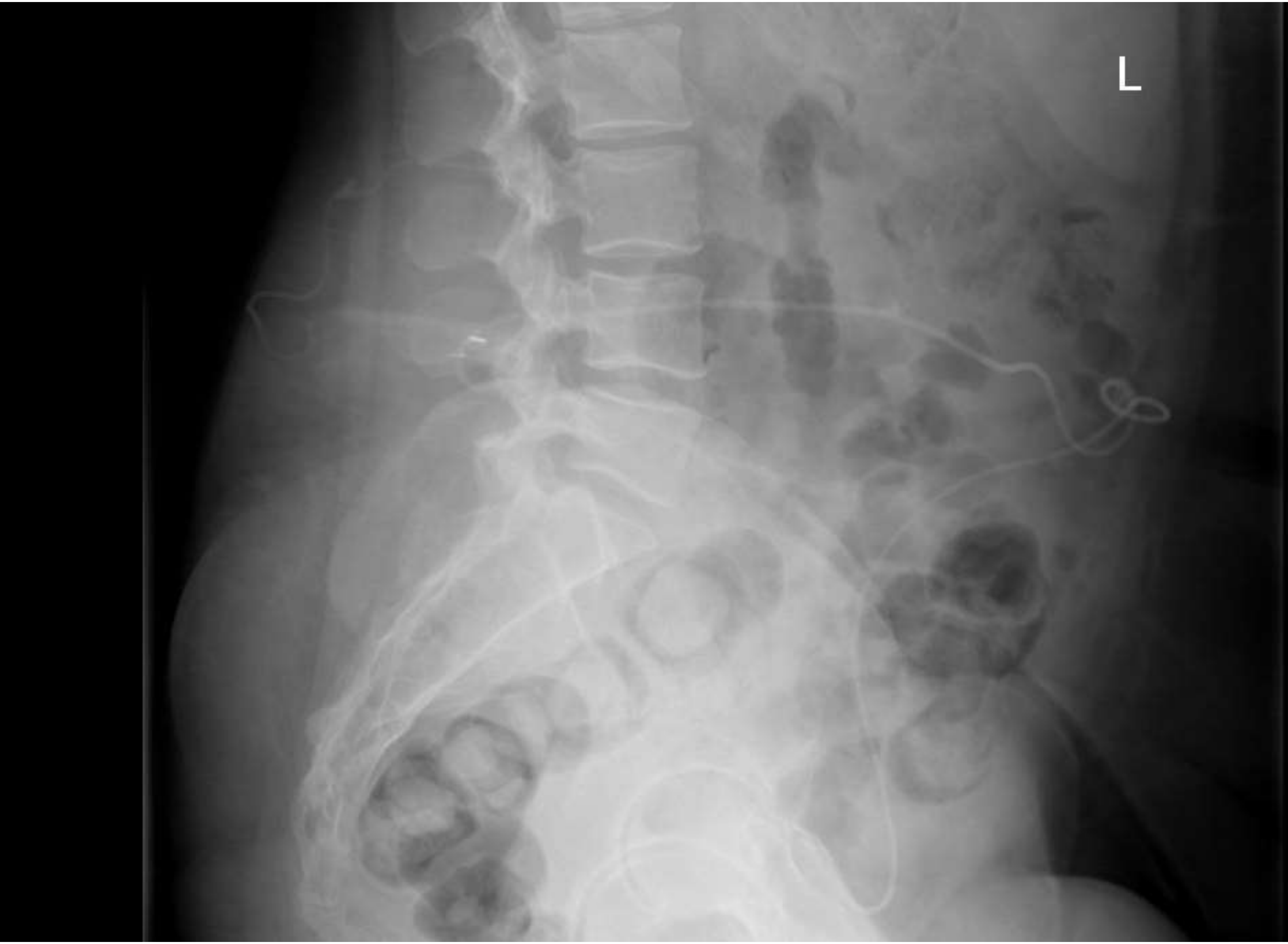
L

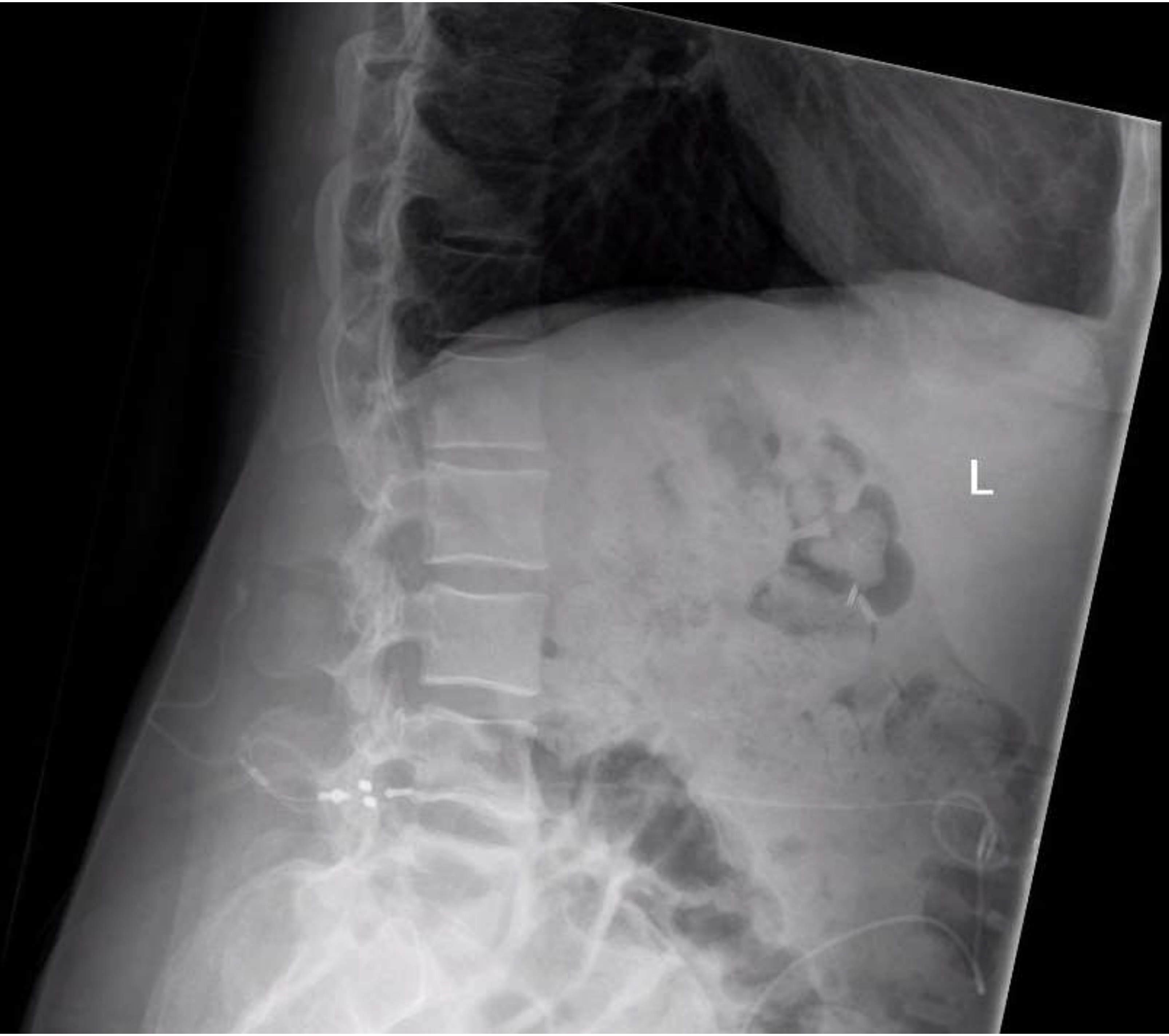
L

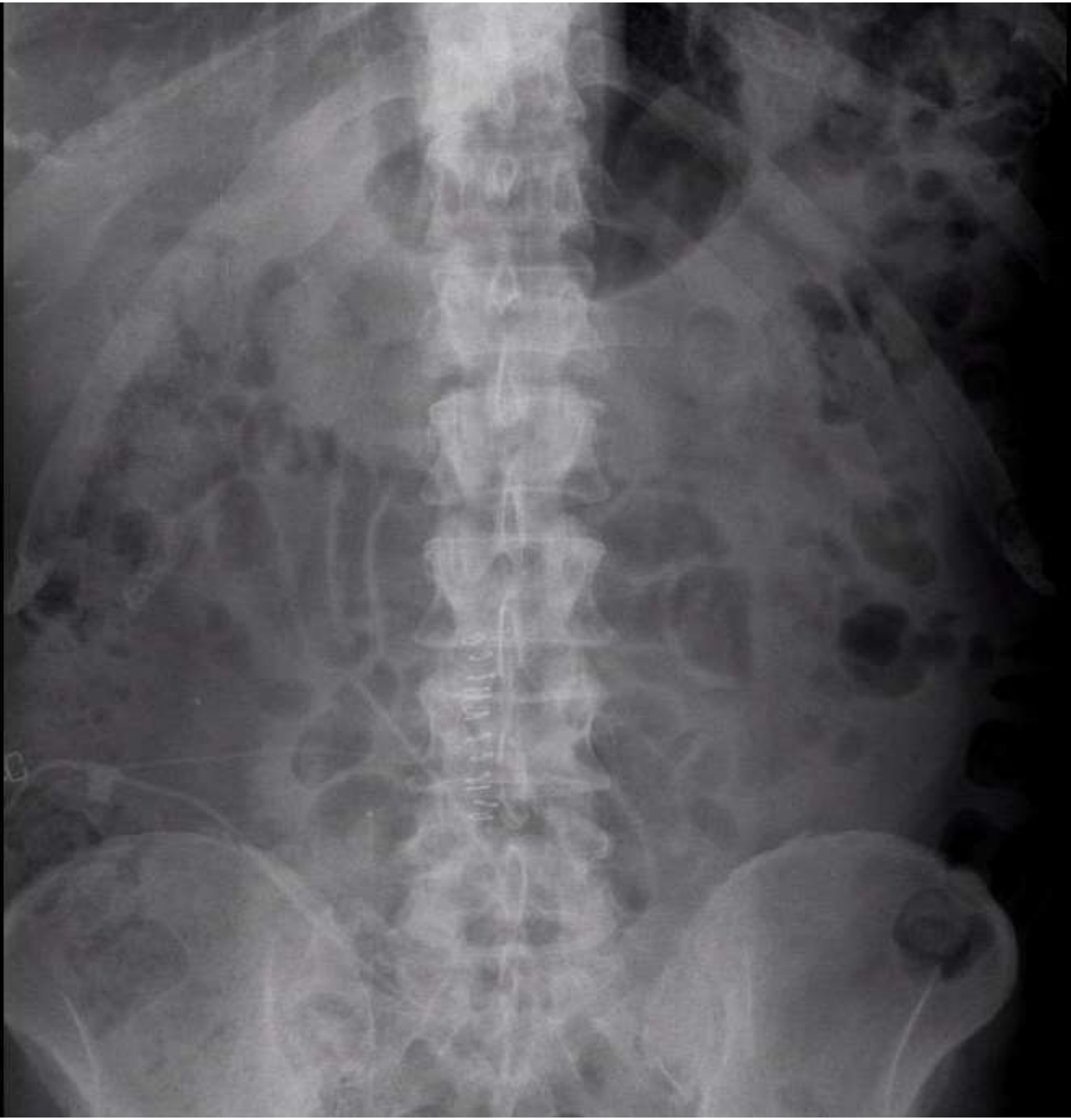

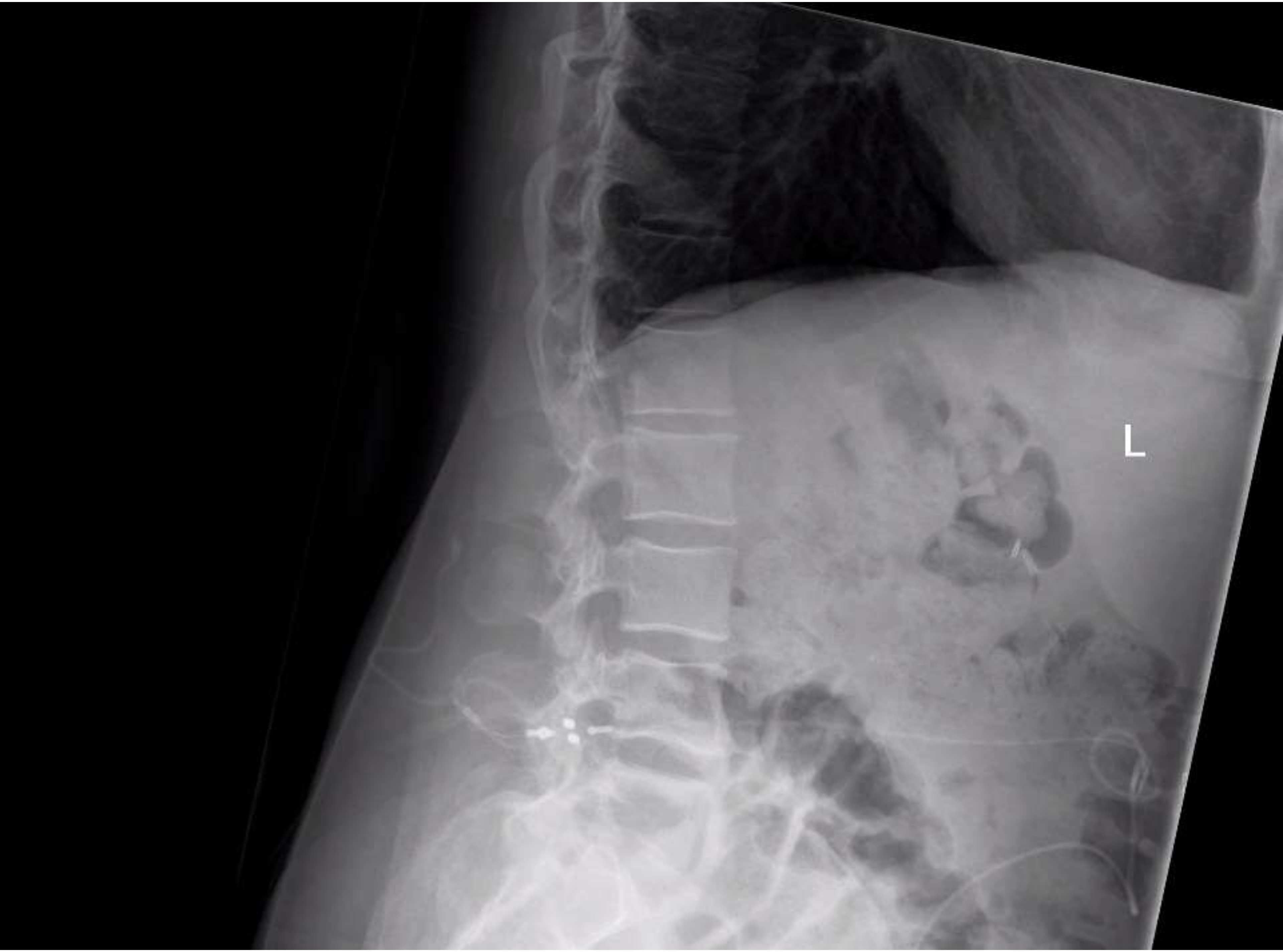
L

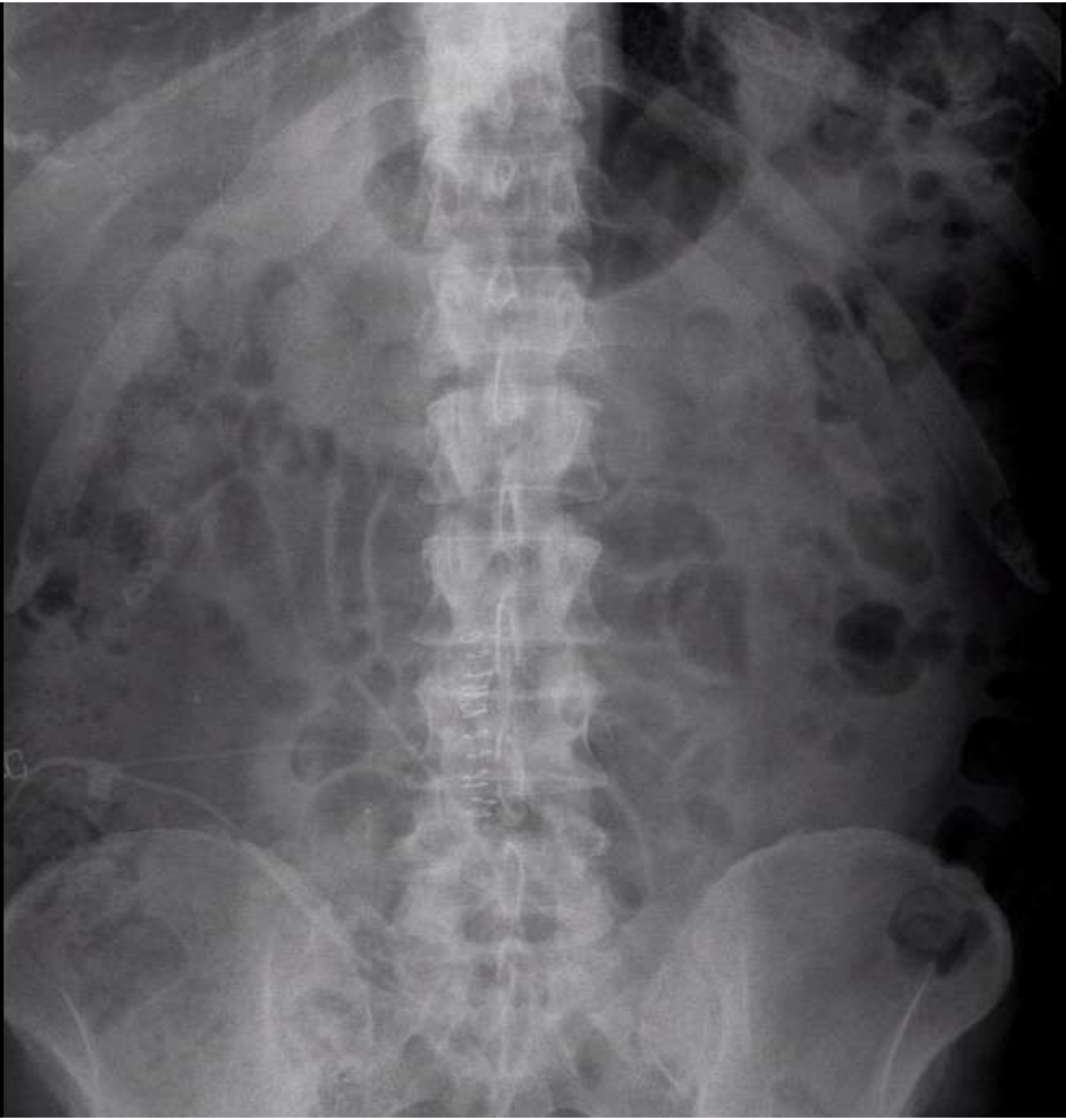

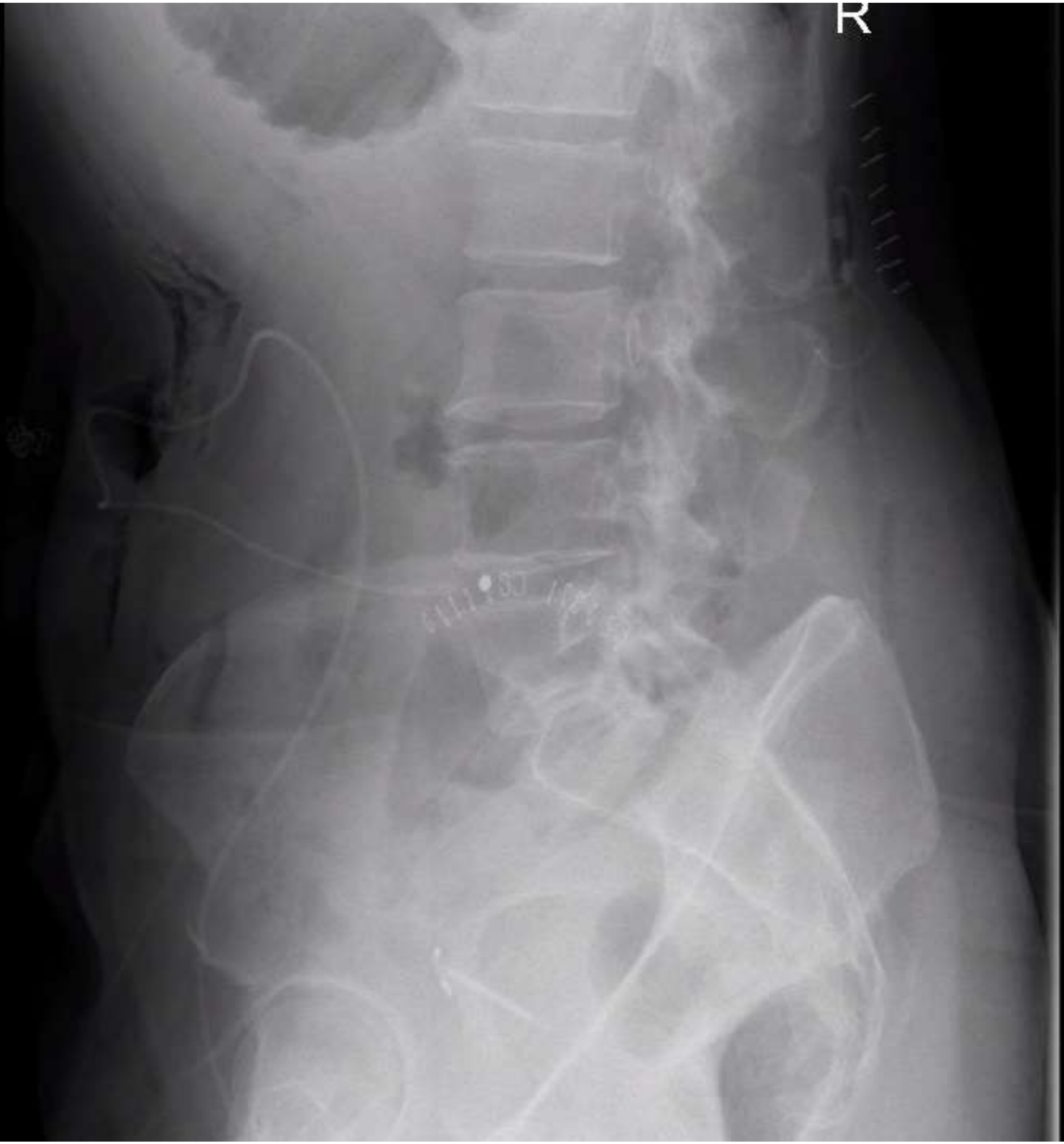
R

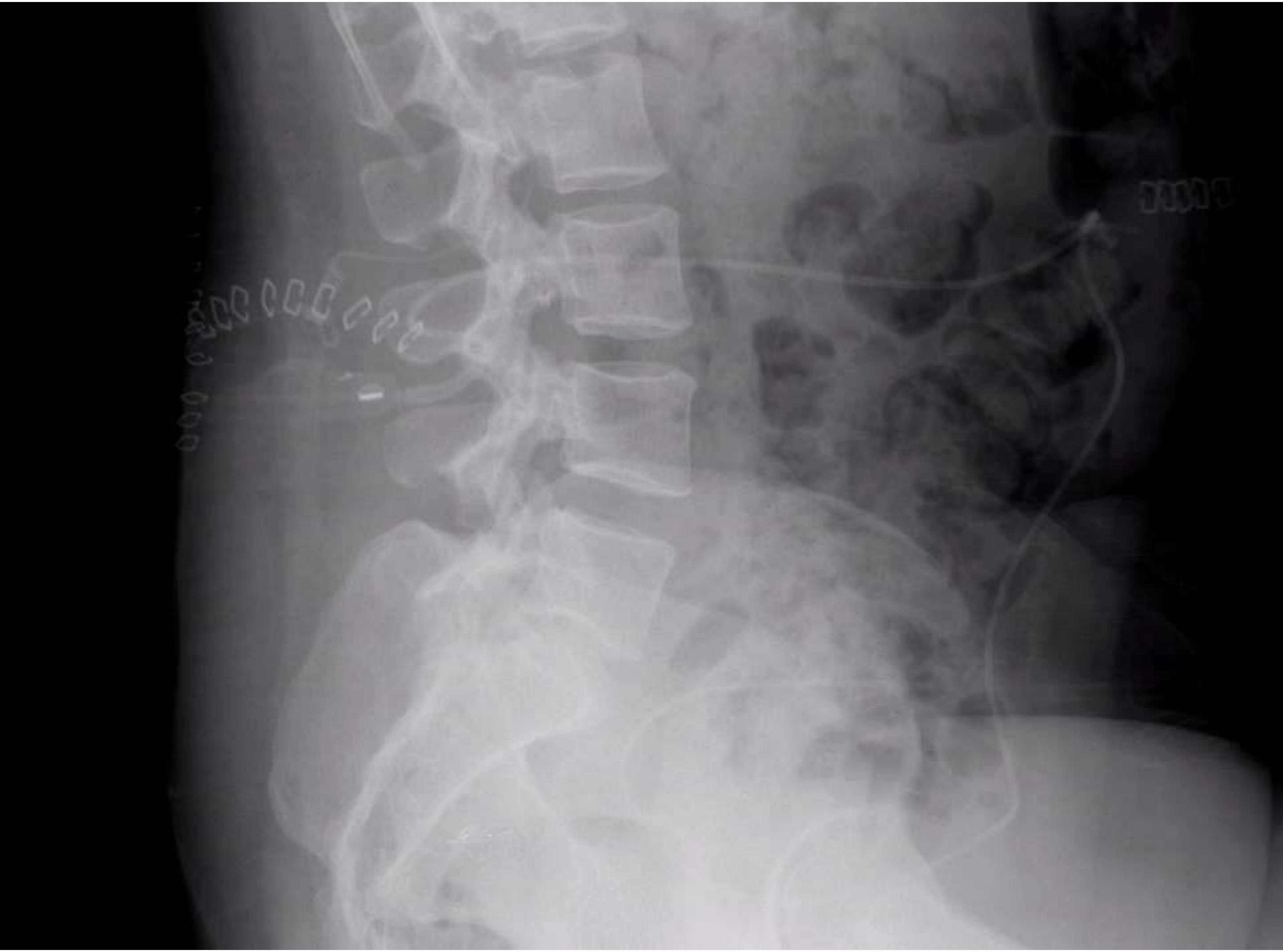

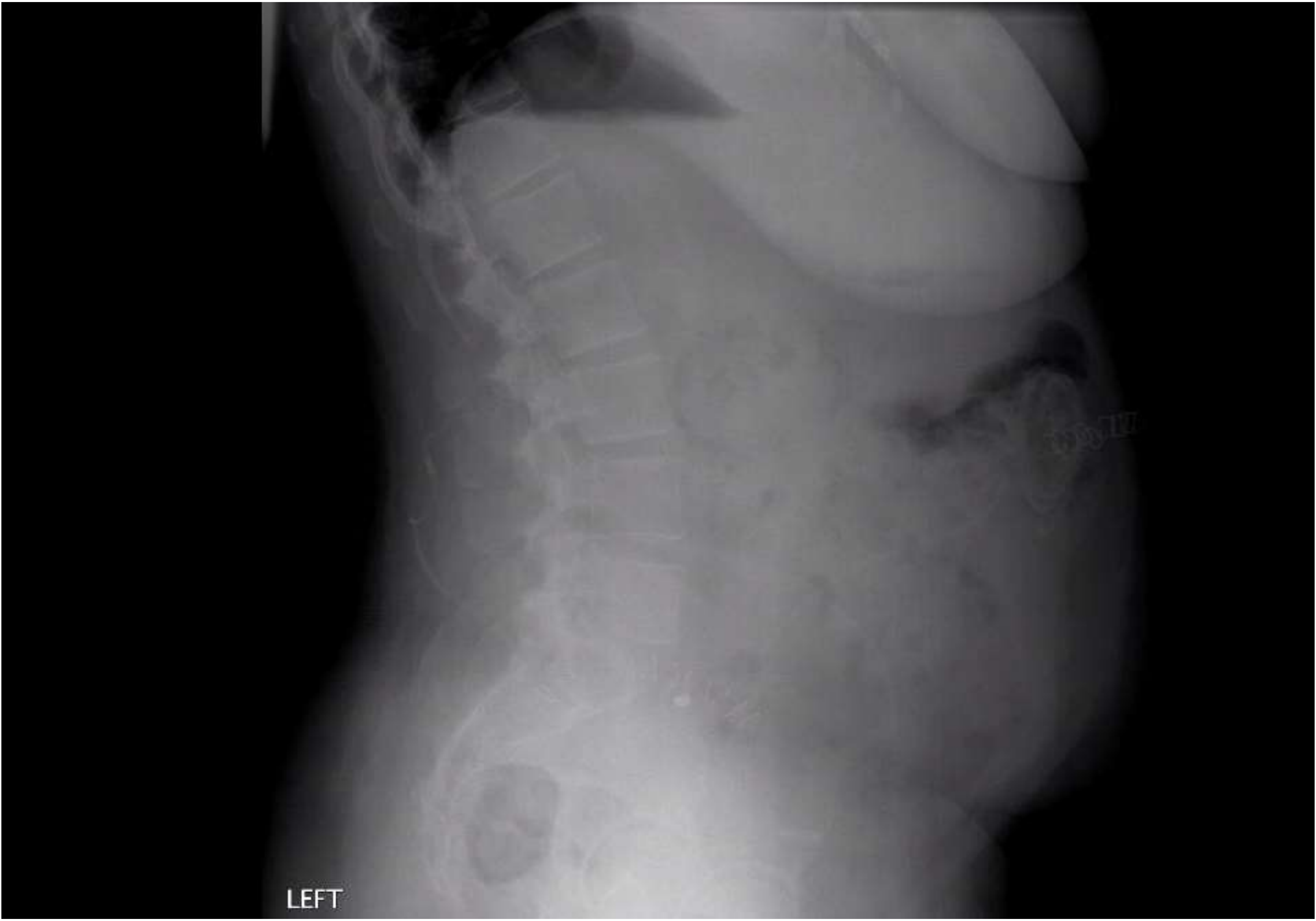
LEFT

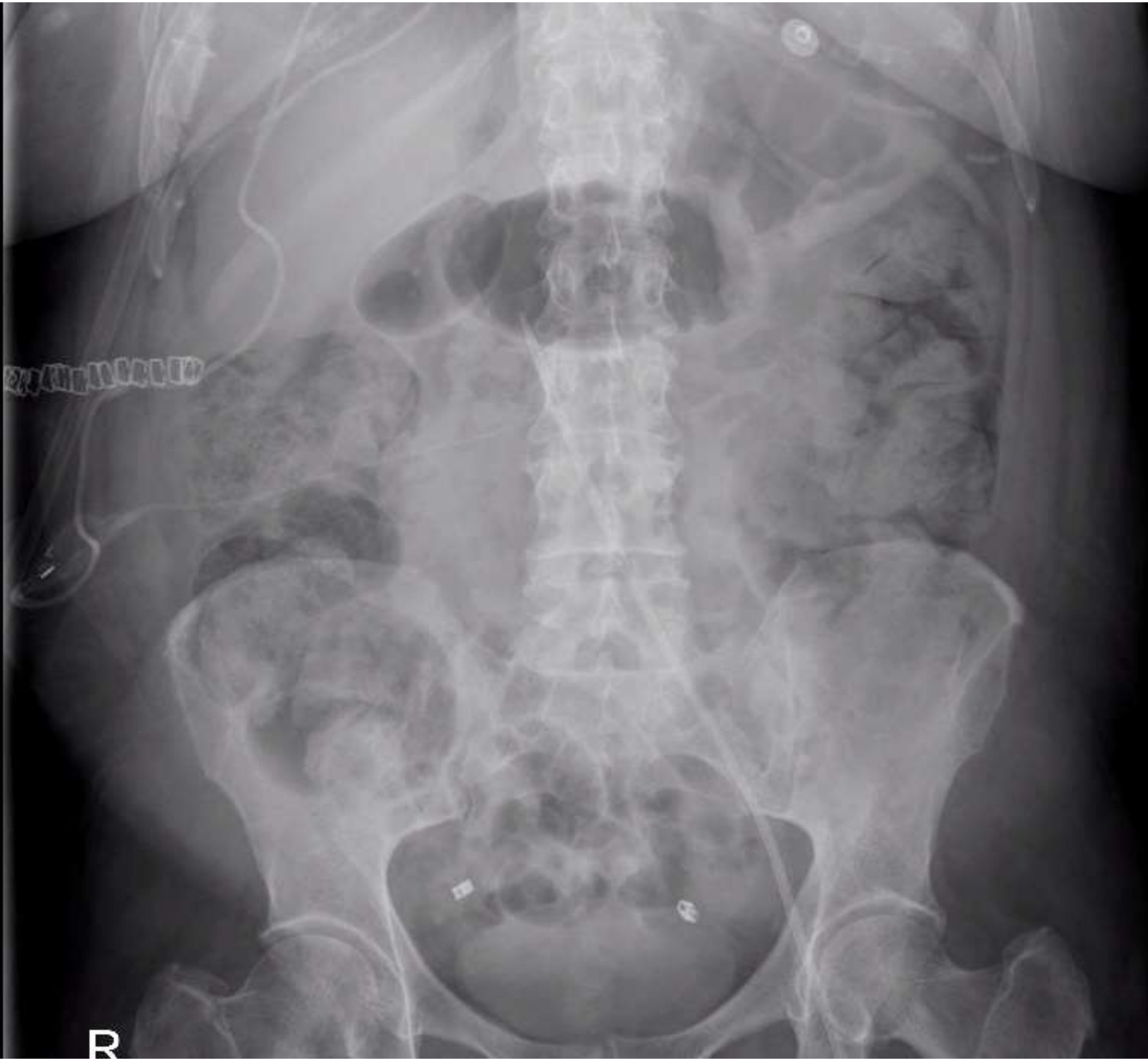
R

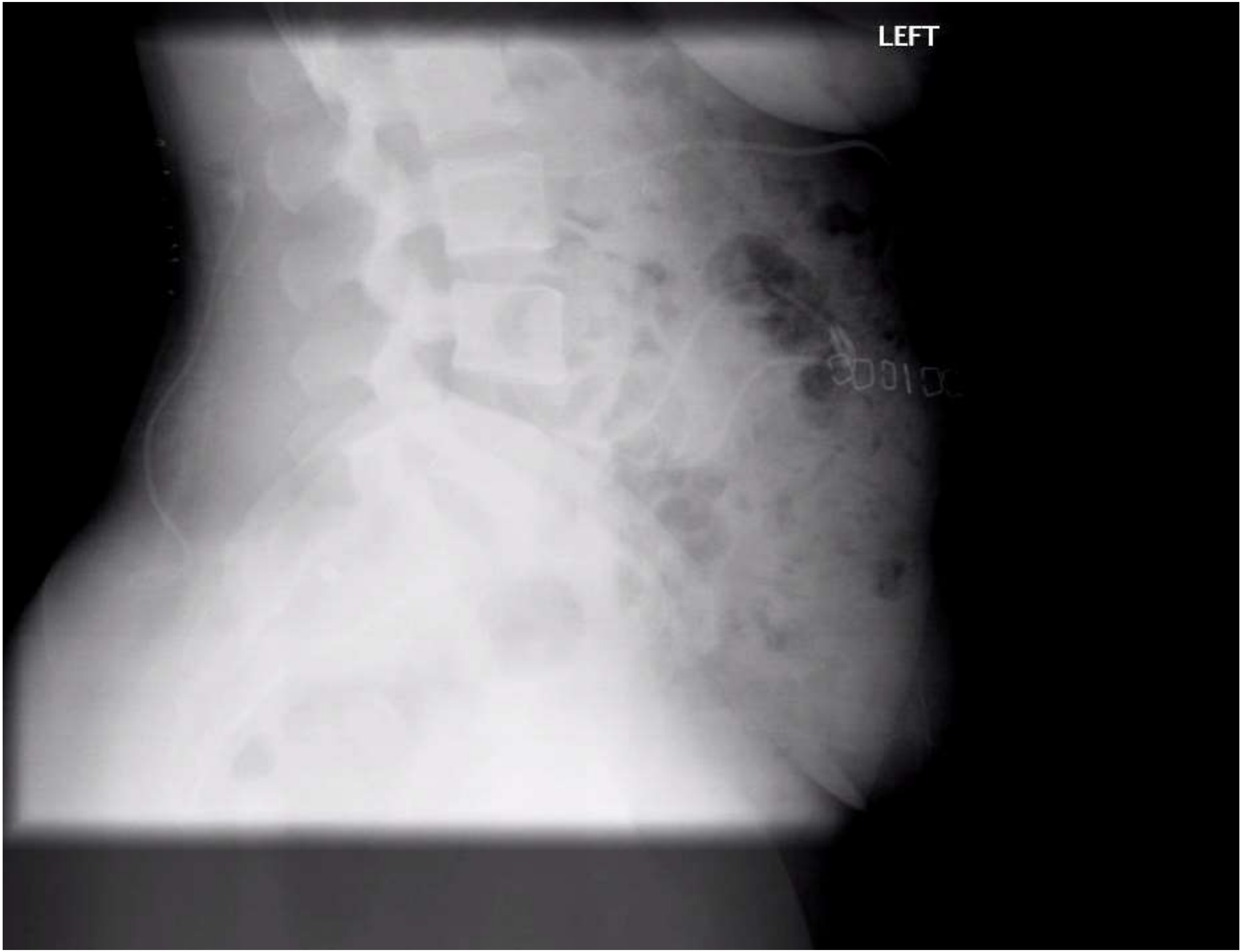
LEFT

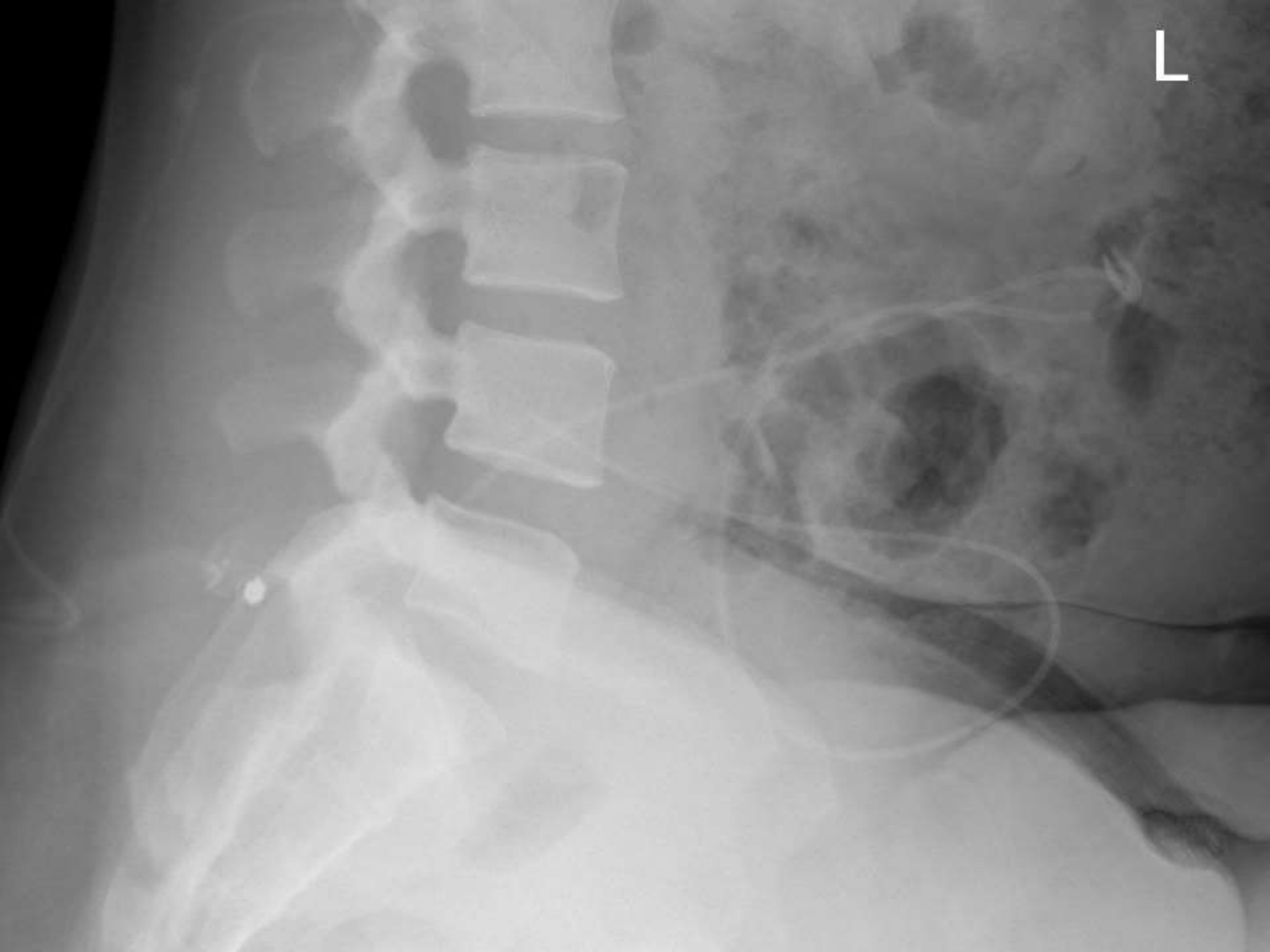
L

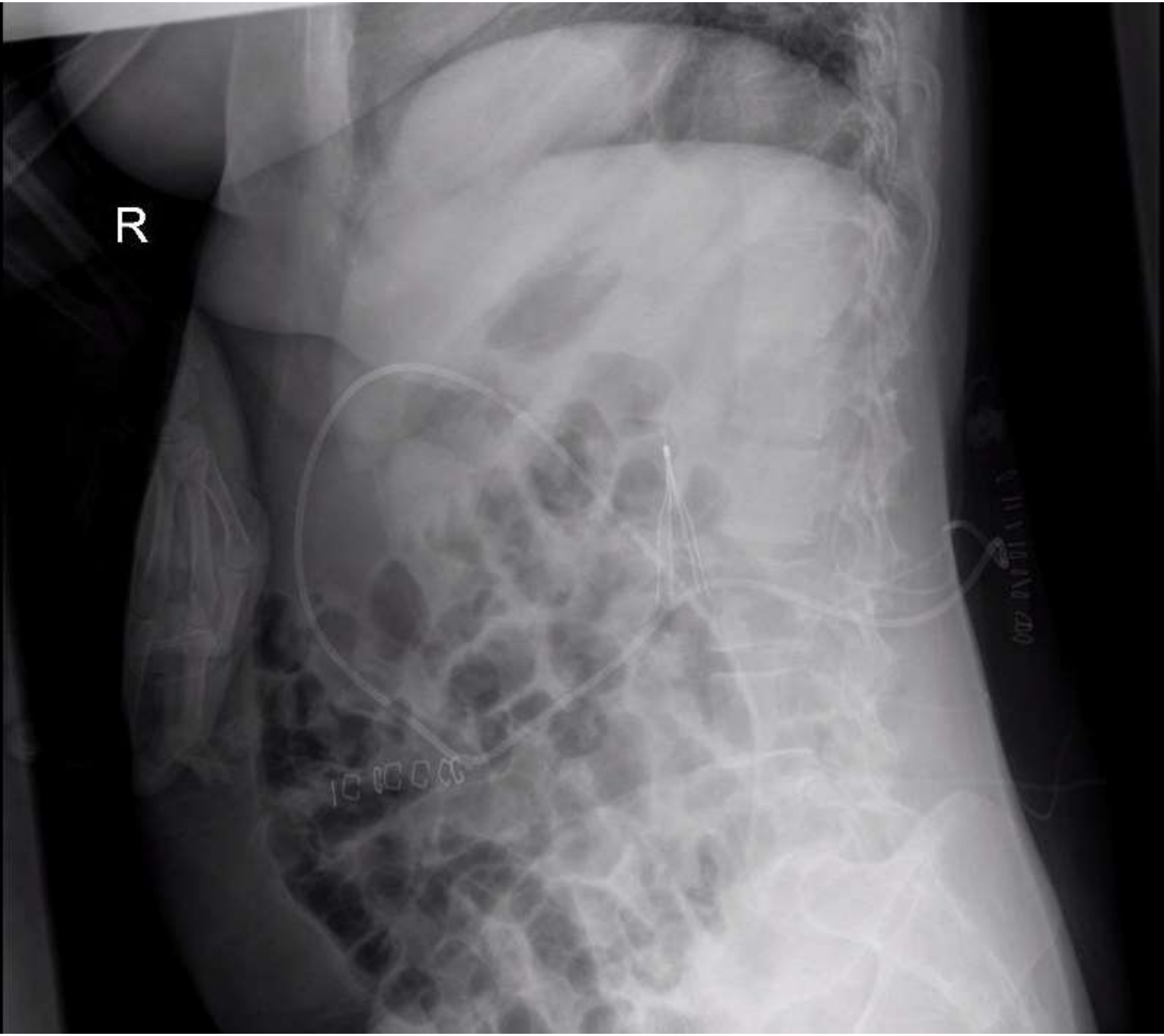
R

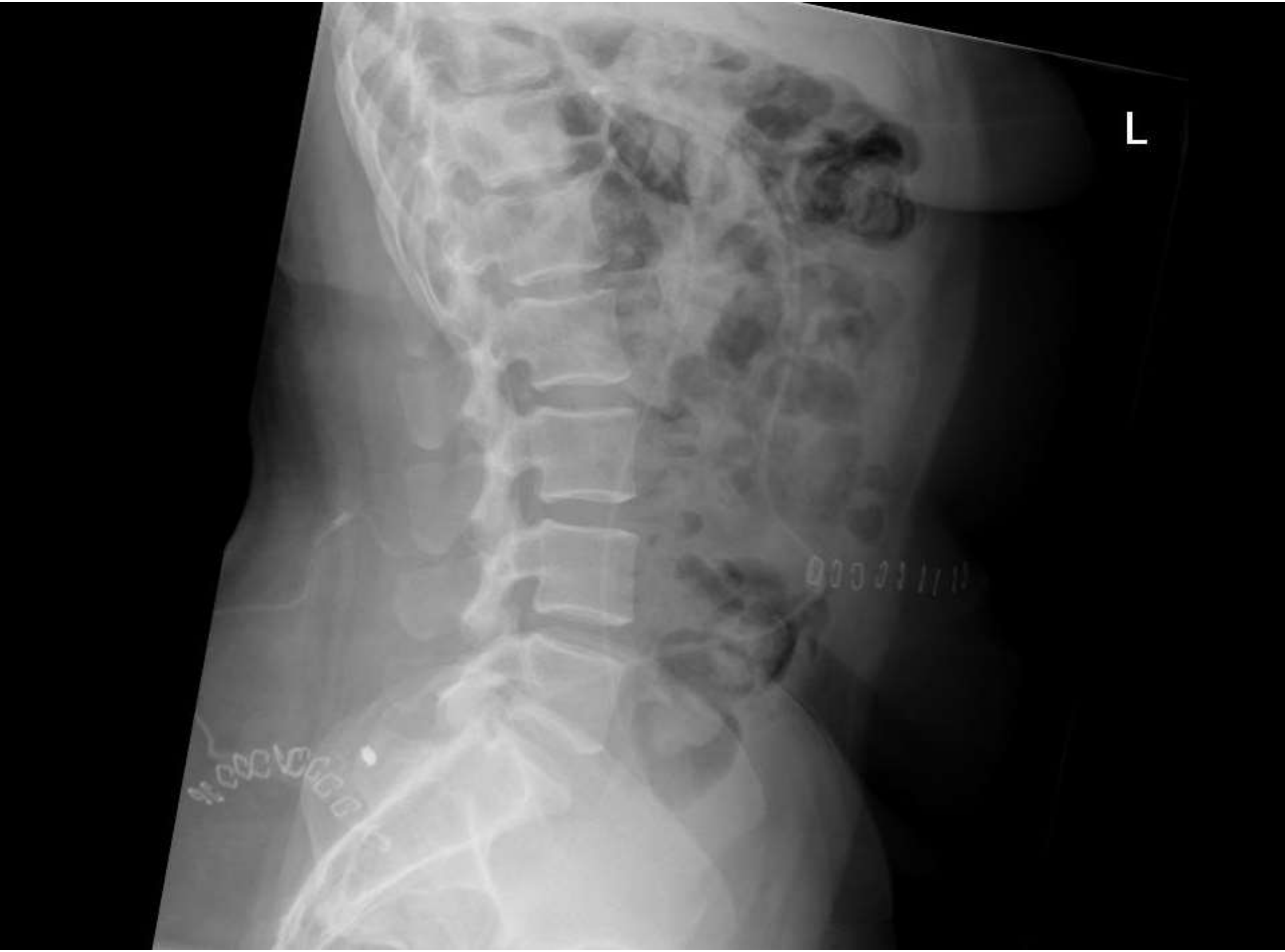
L

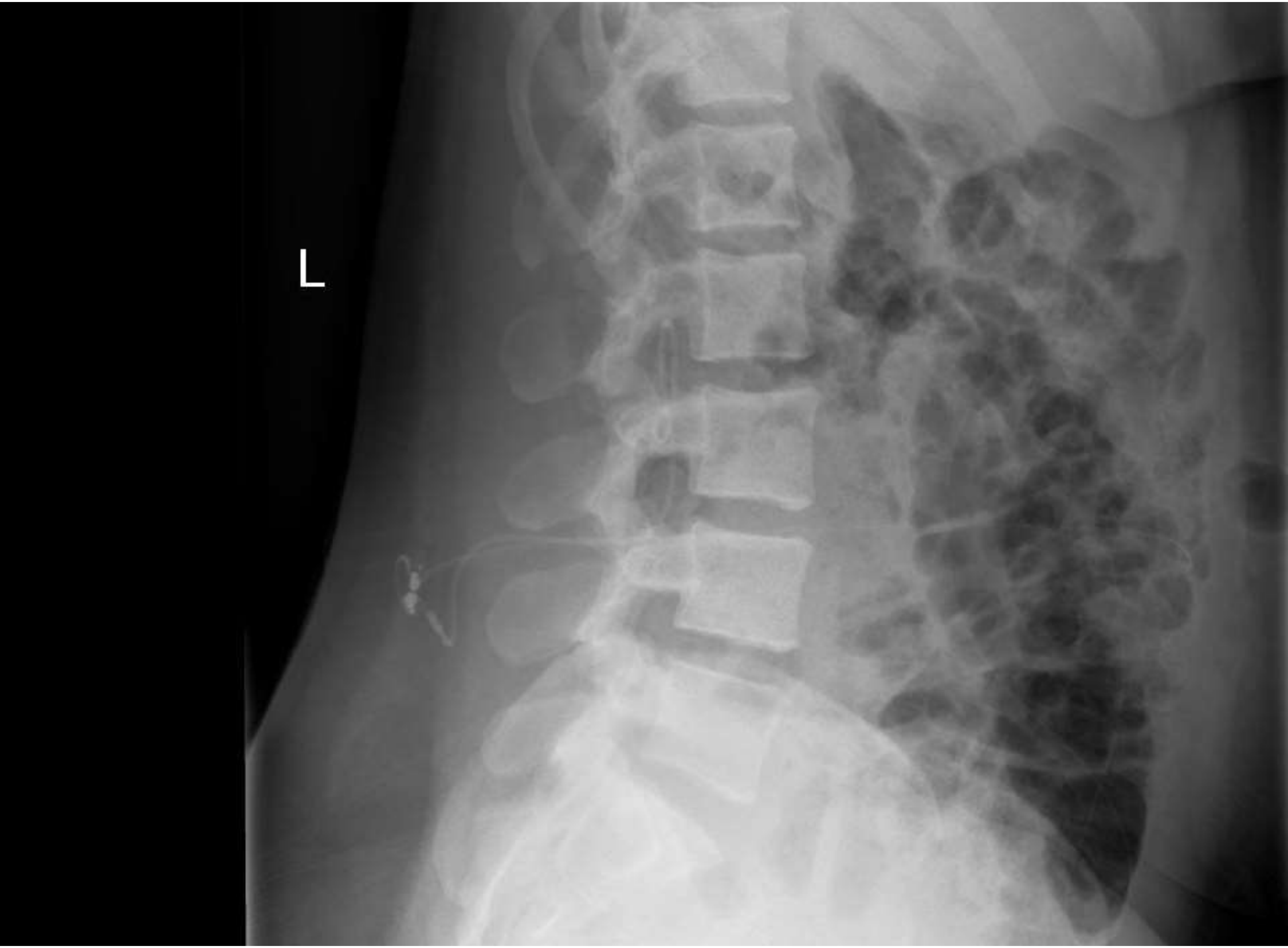
L

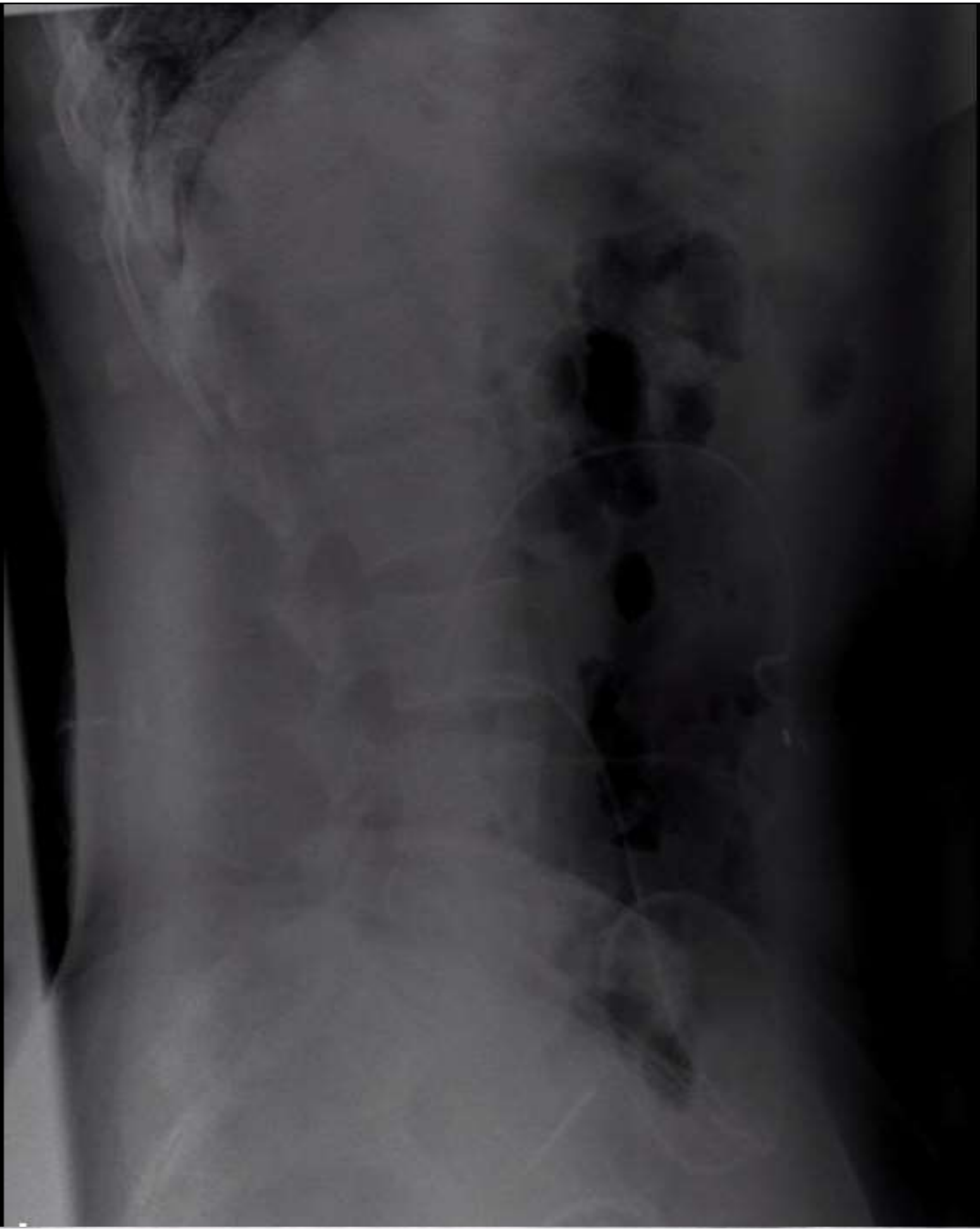

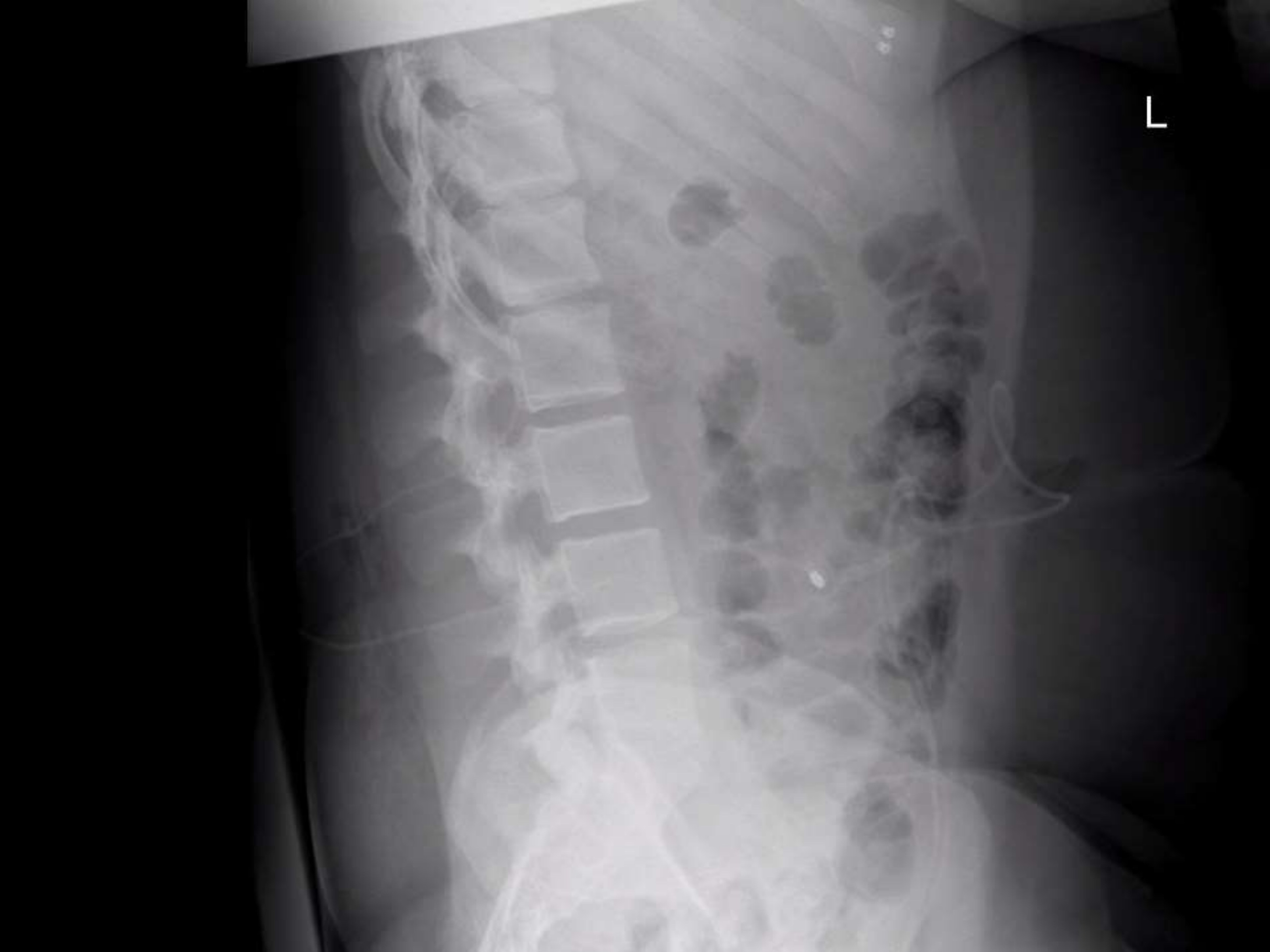
L

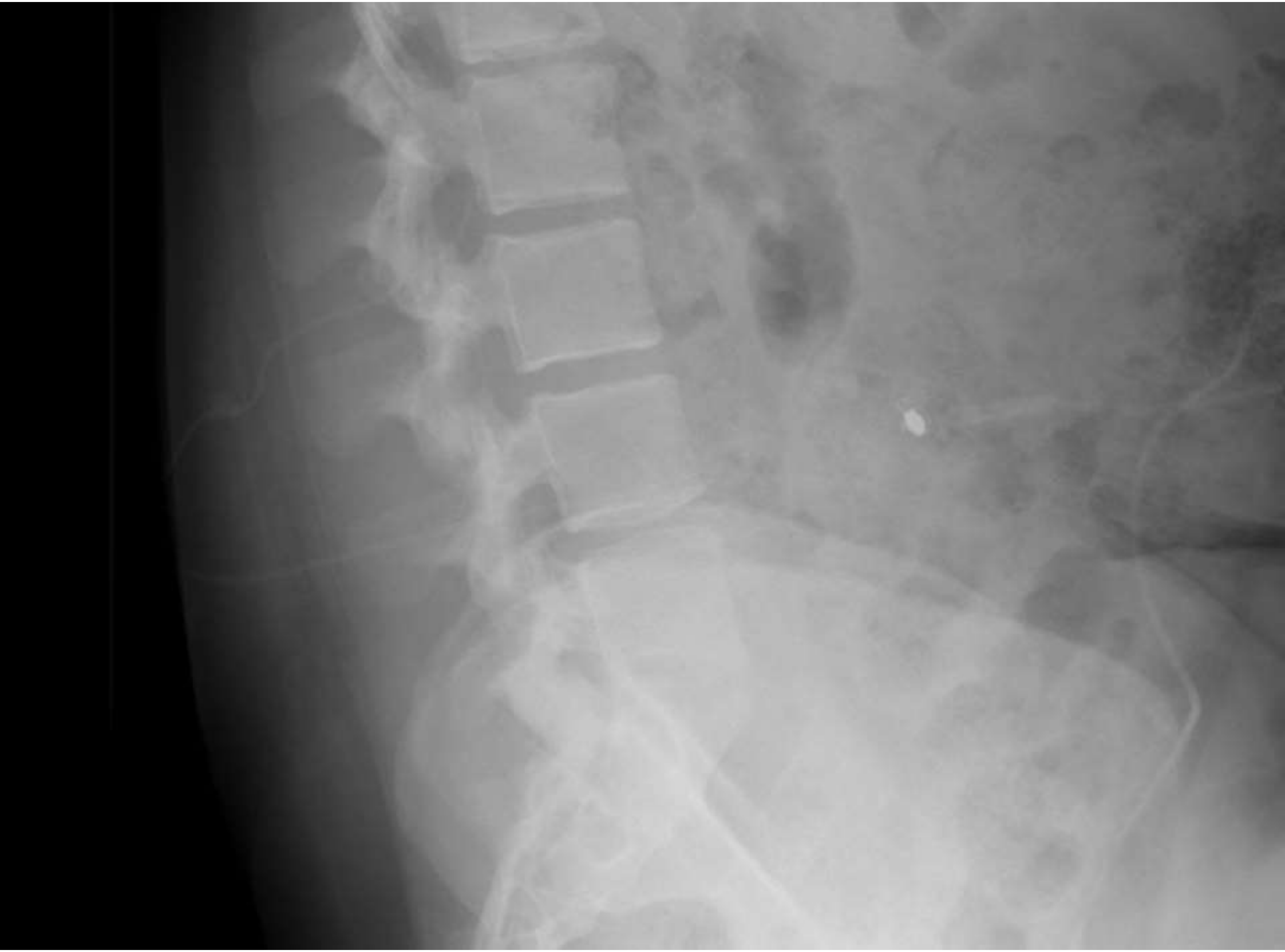

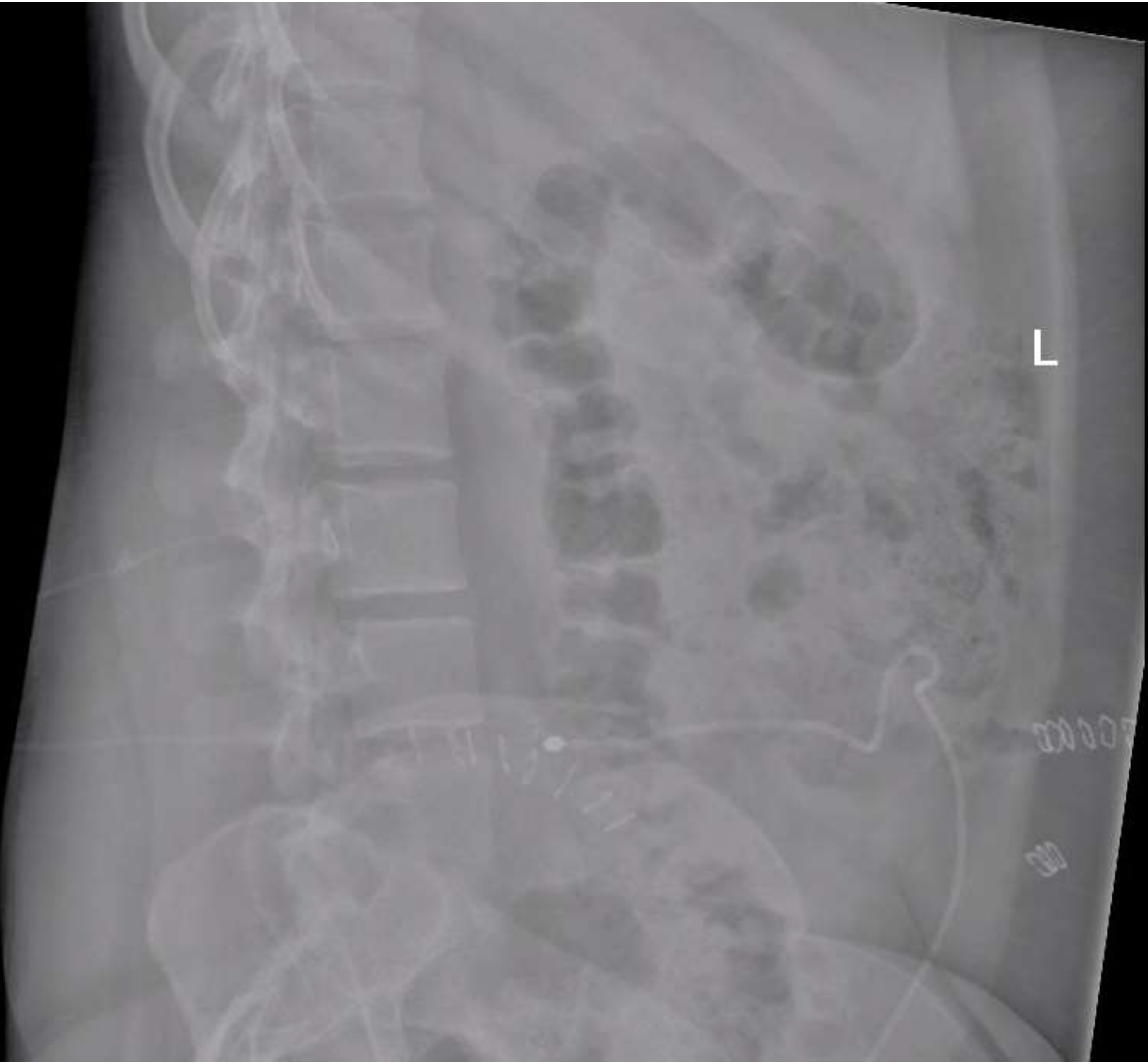
L

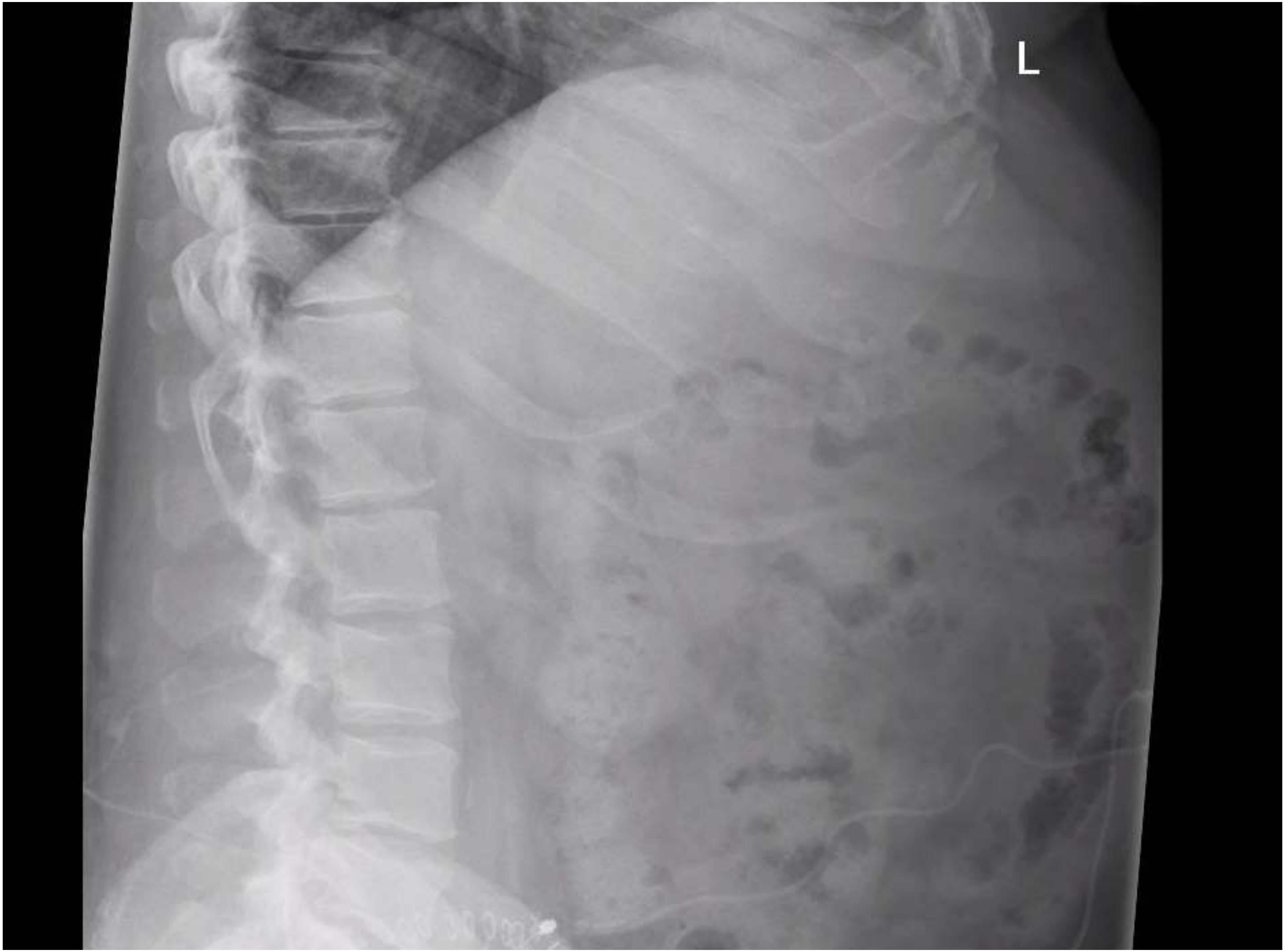
L

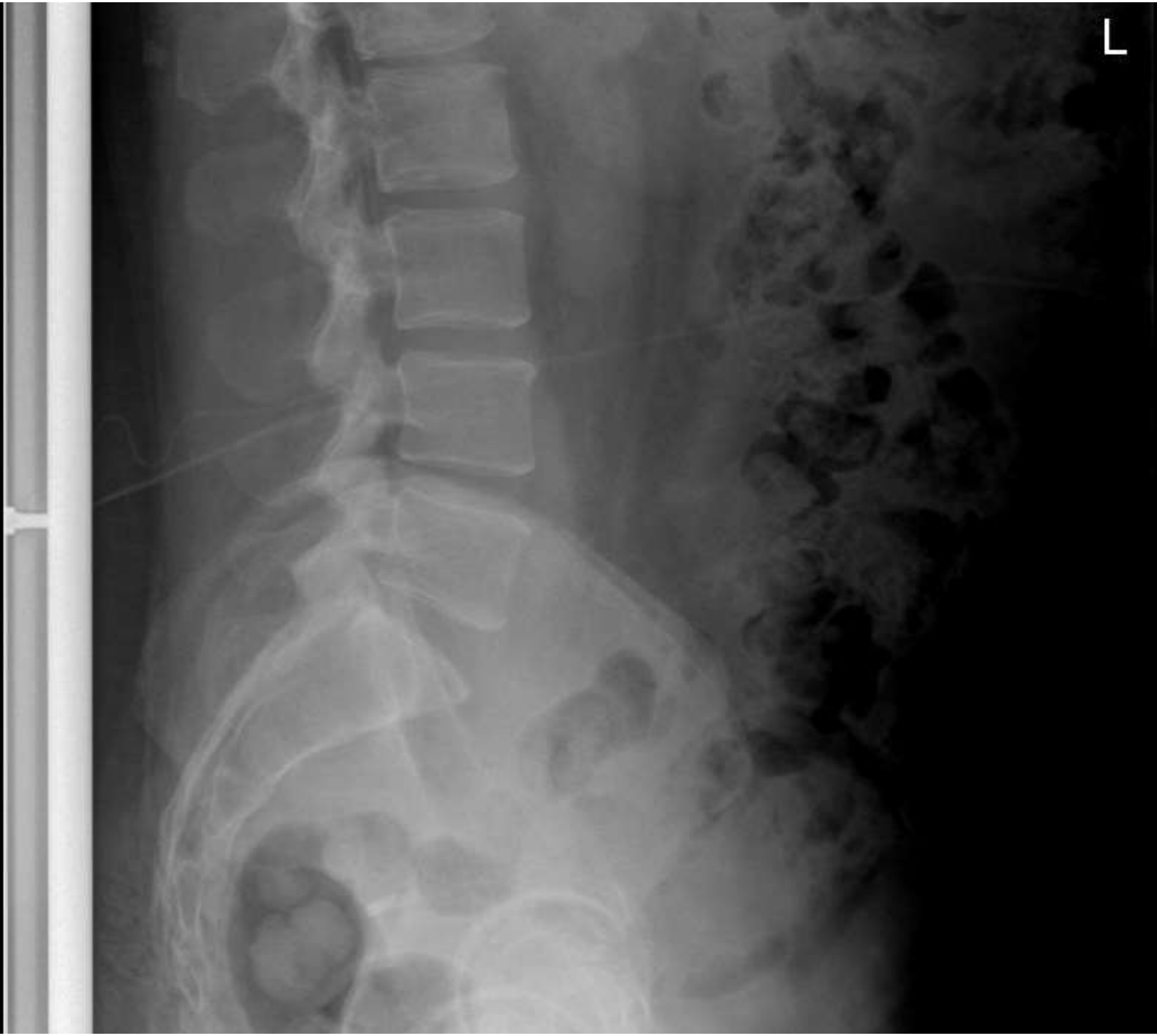
L

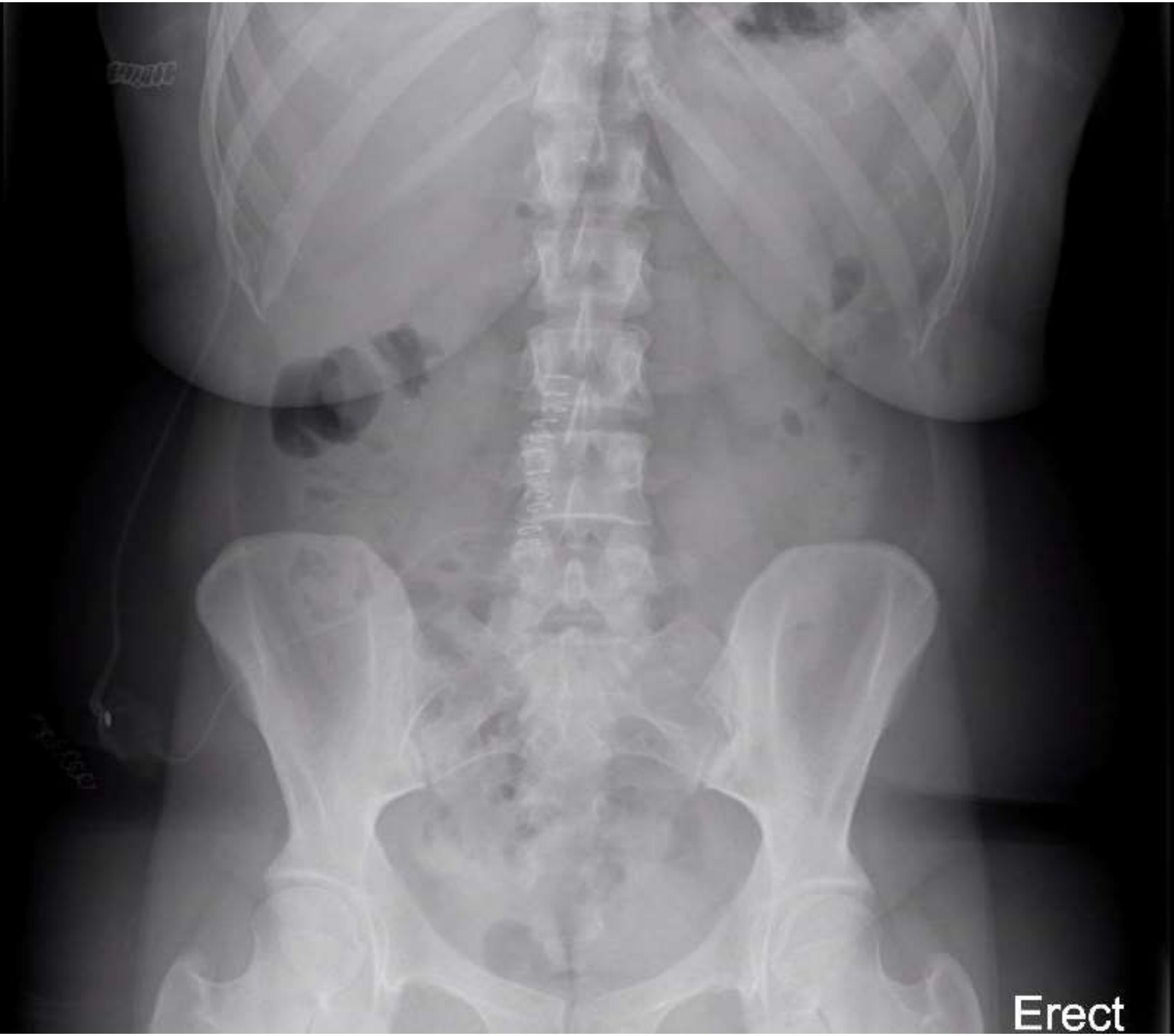
Erect

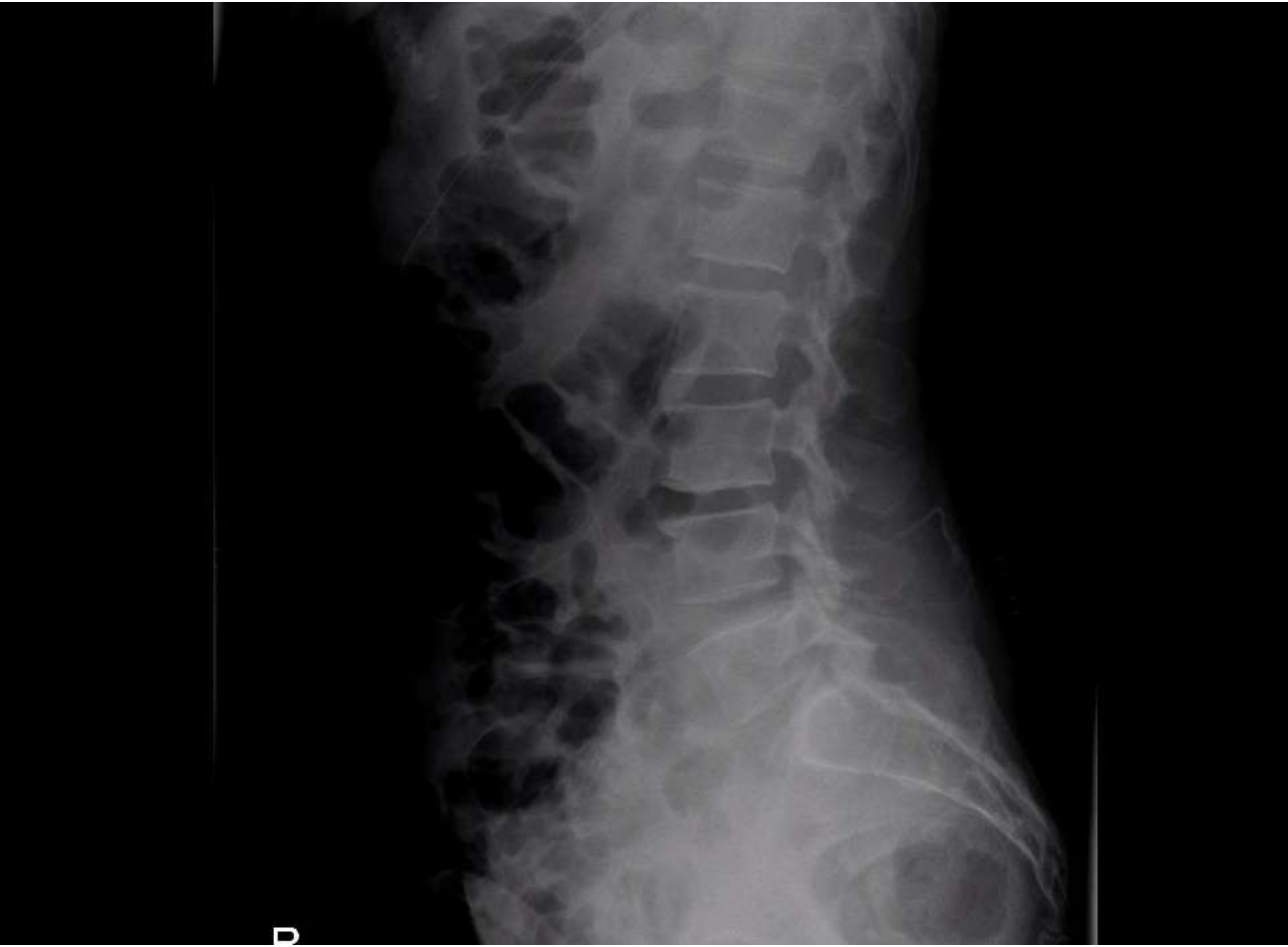

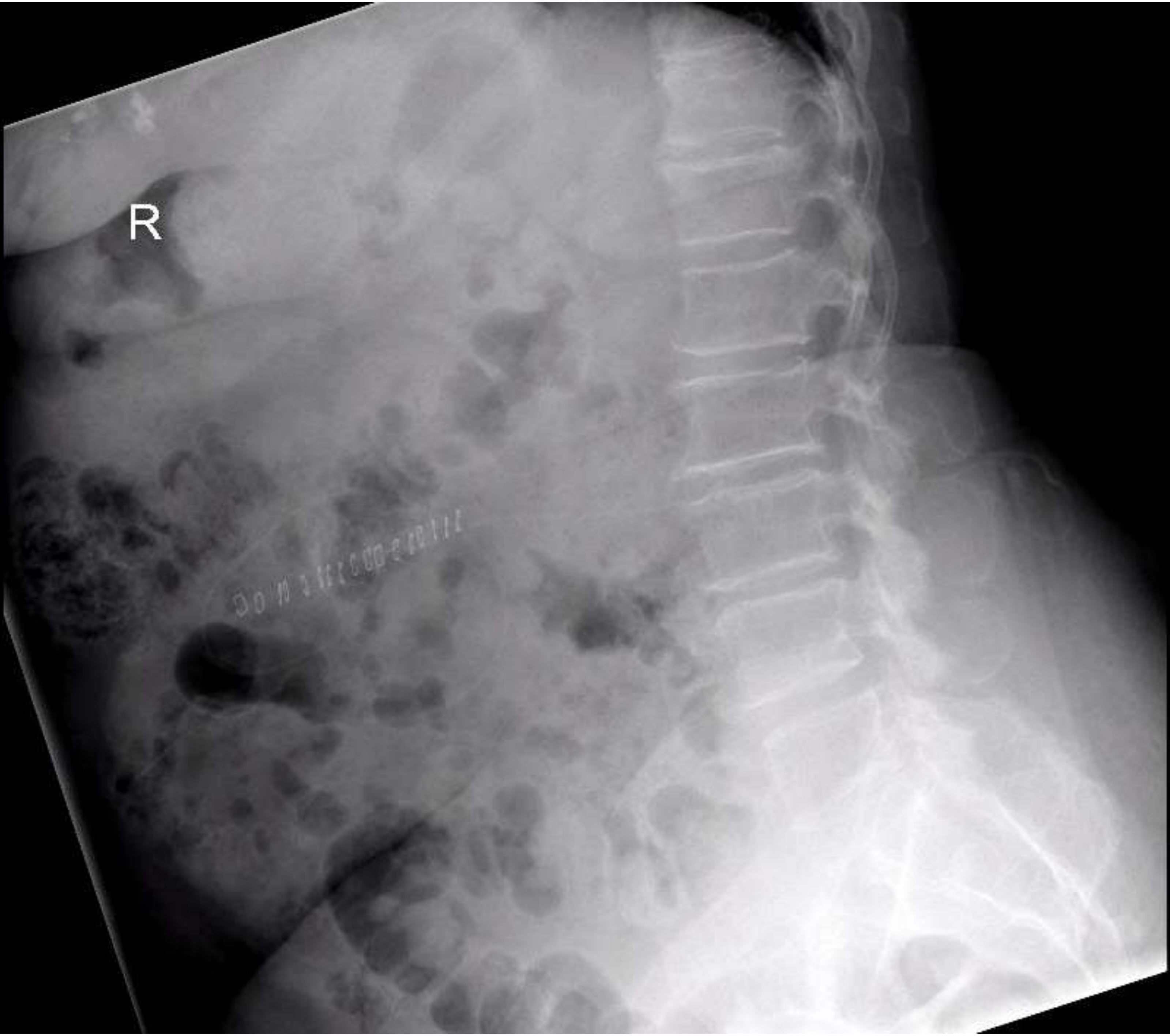
R

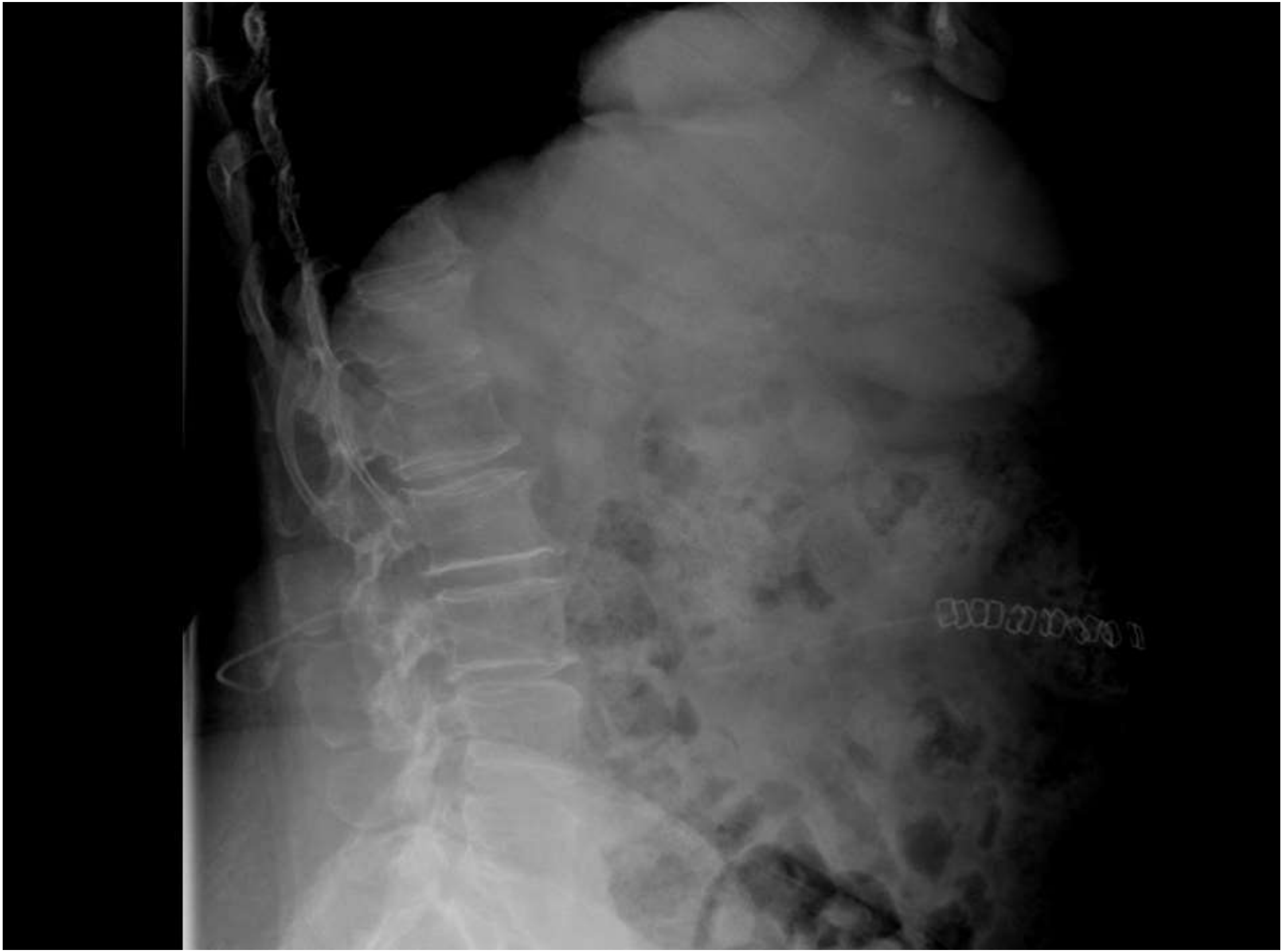

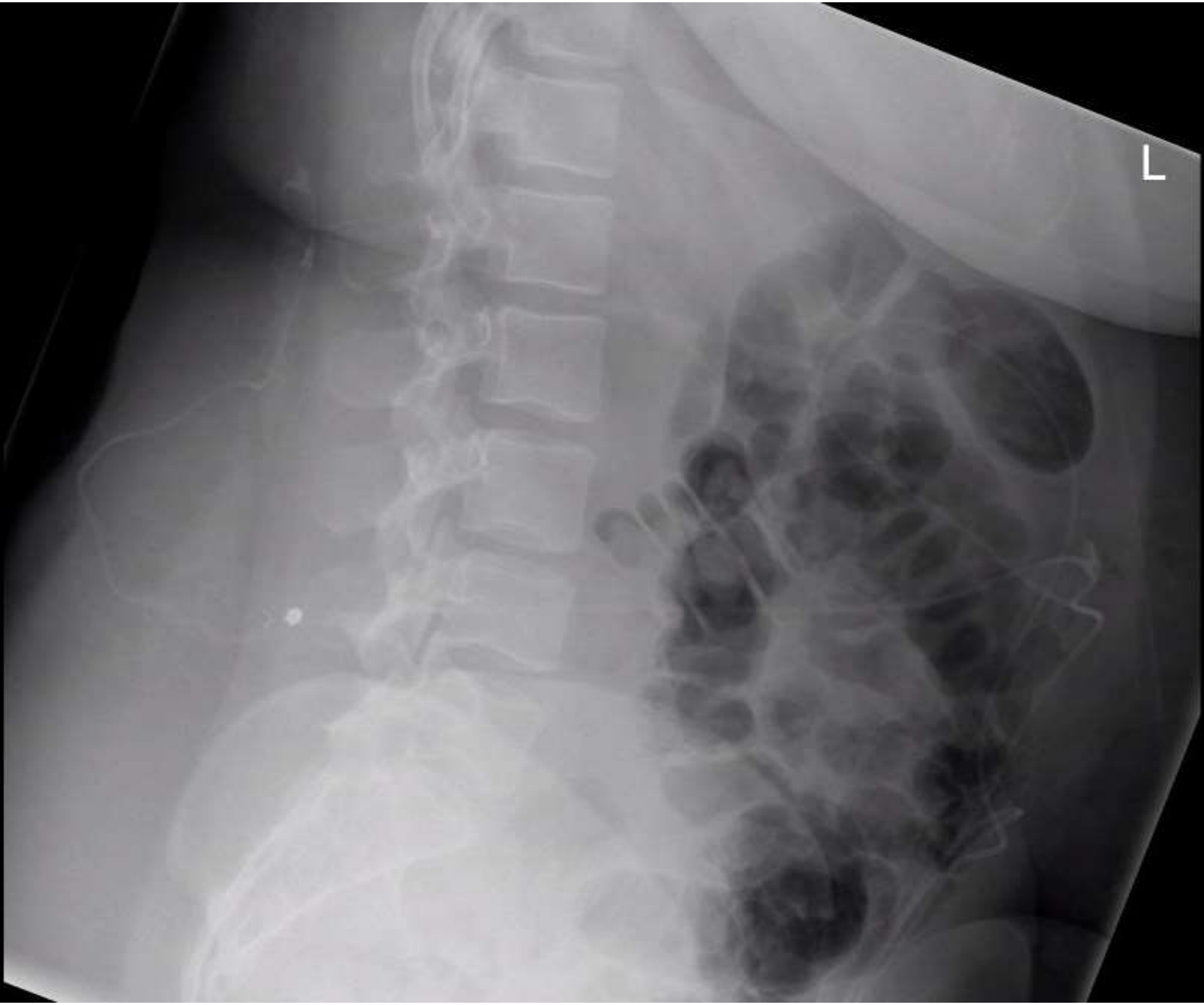
L

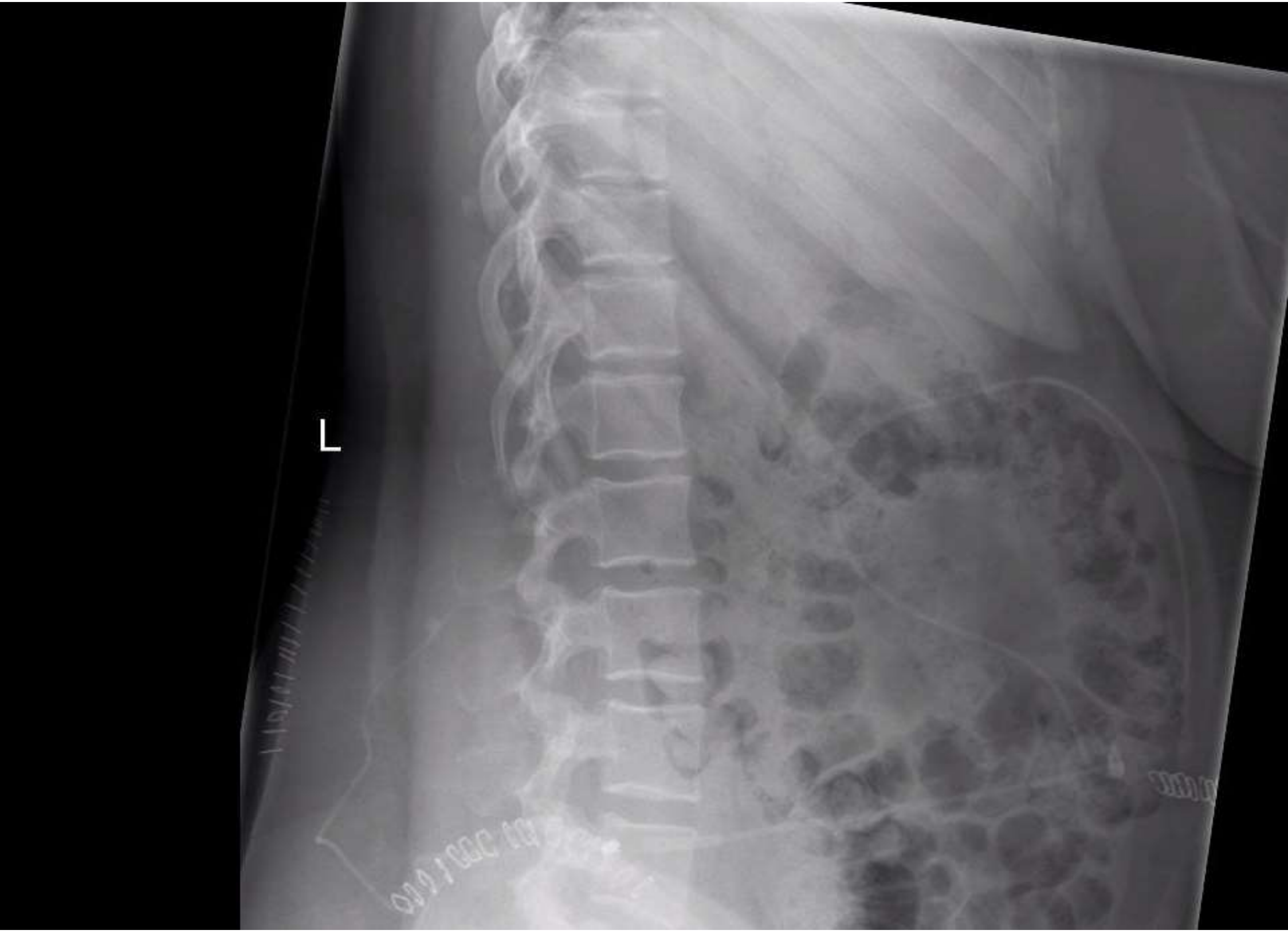
L

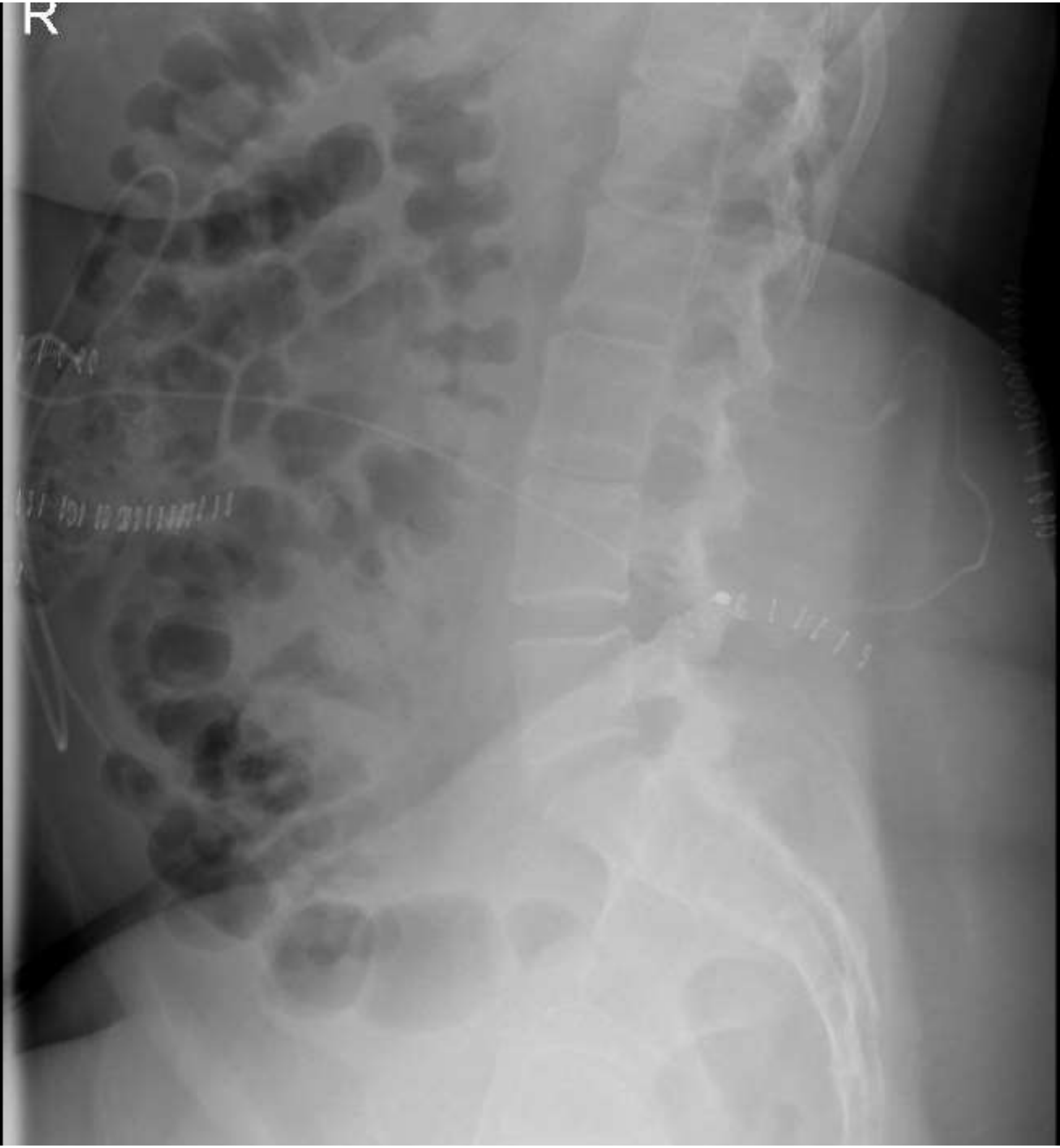
R

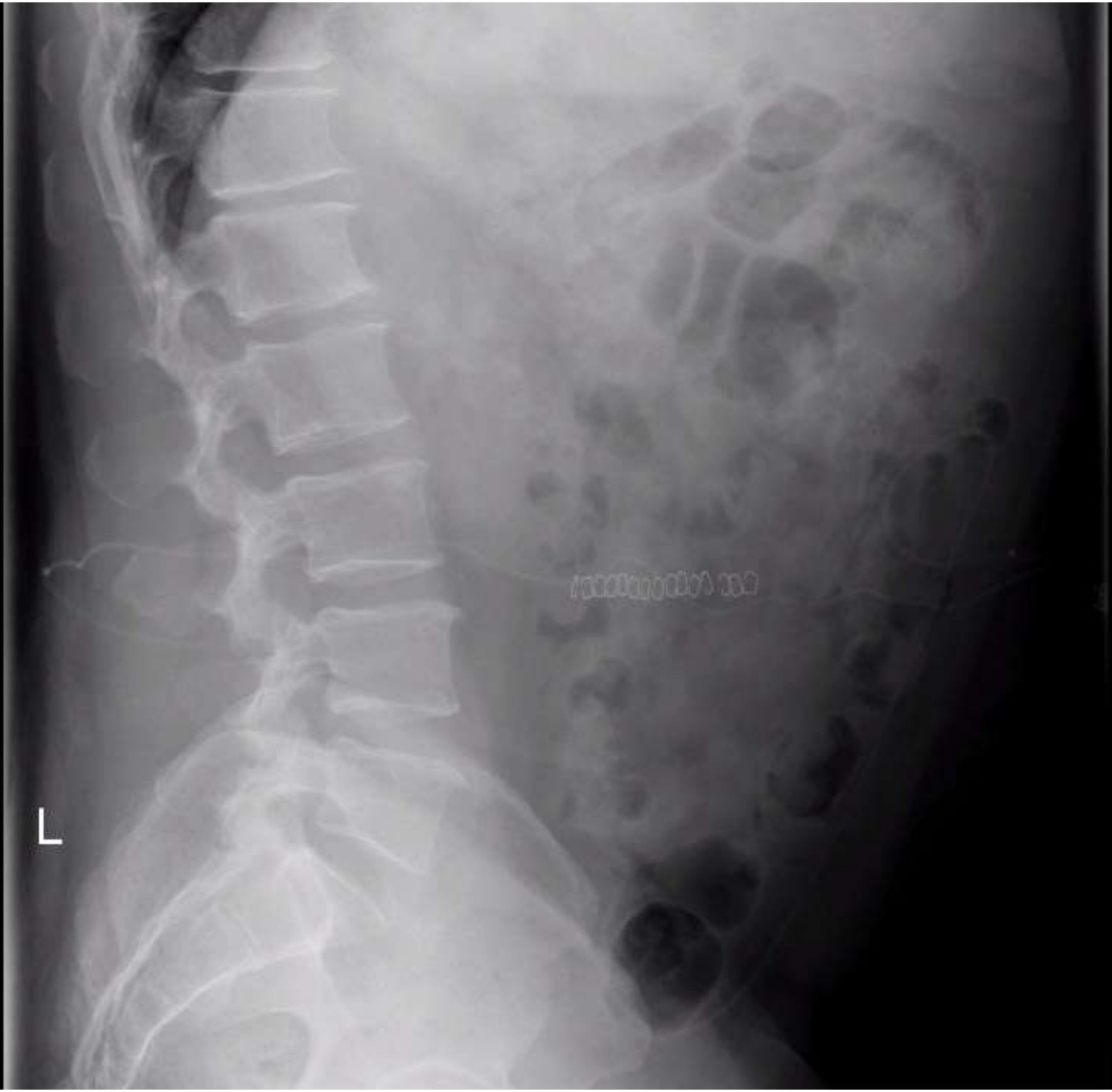
L

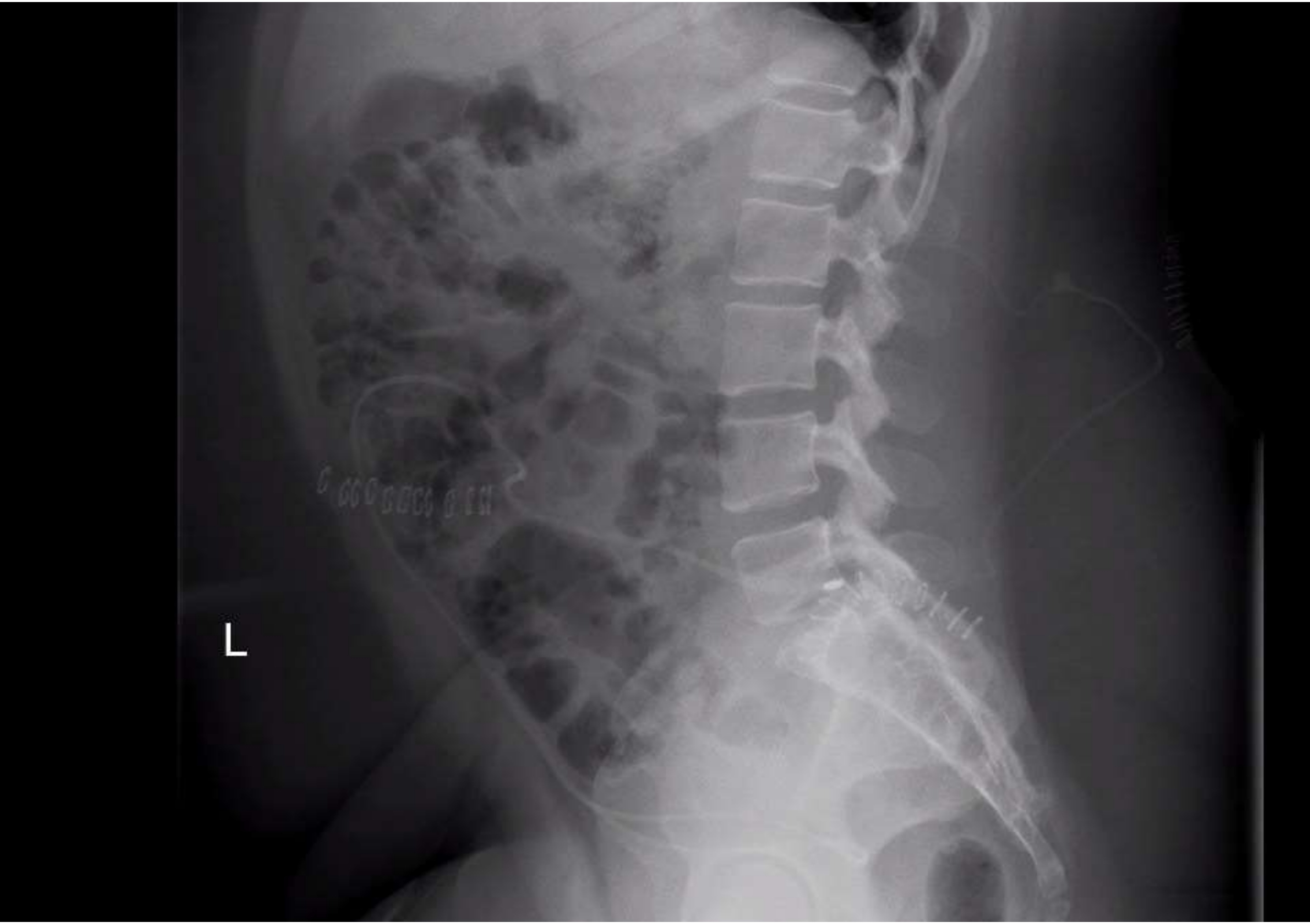
L

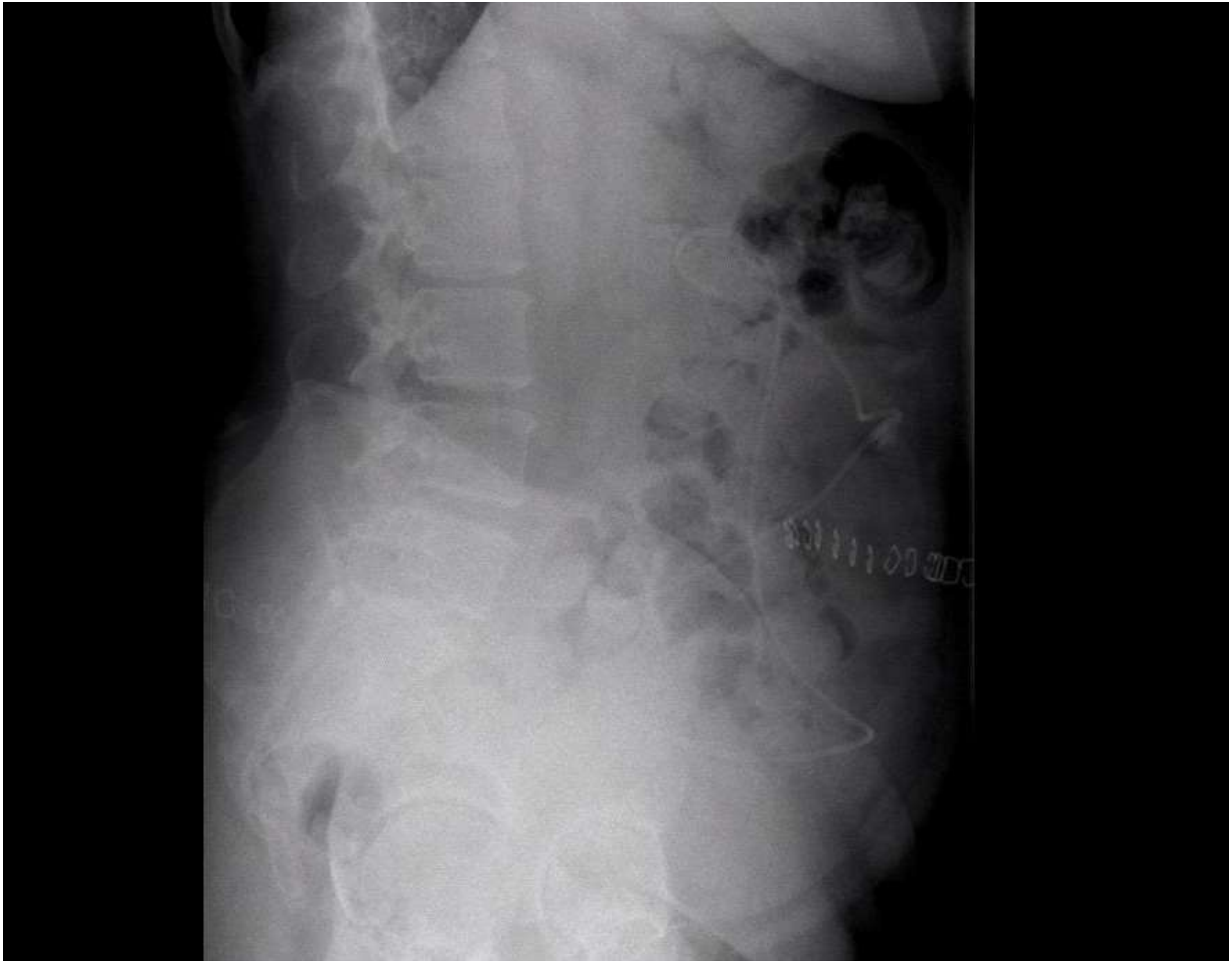

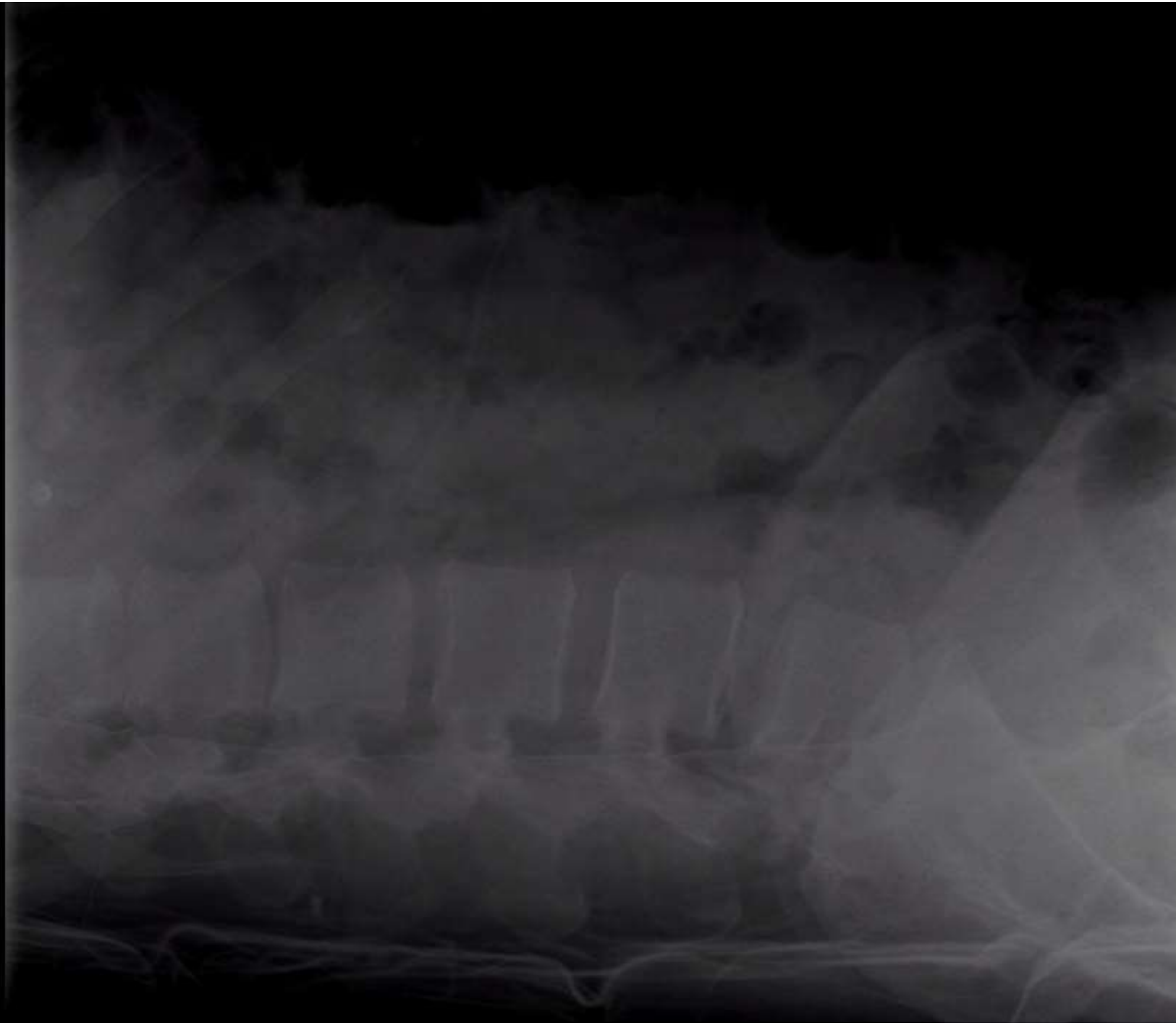

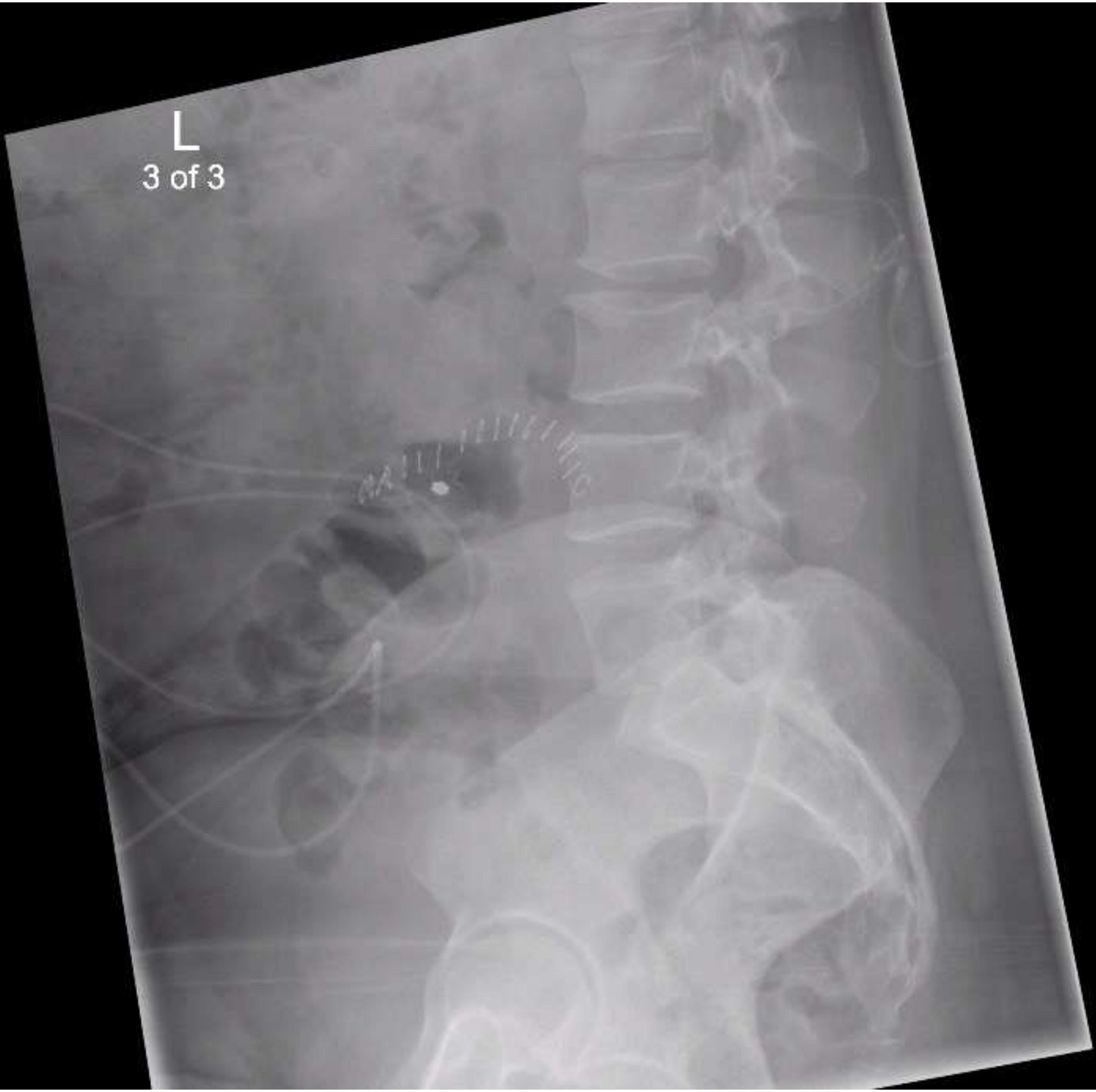
L
3 of 3

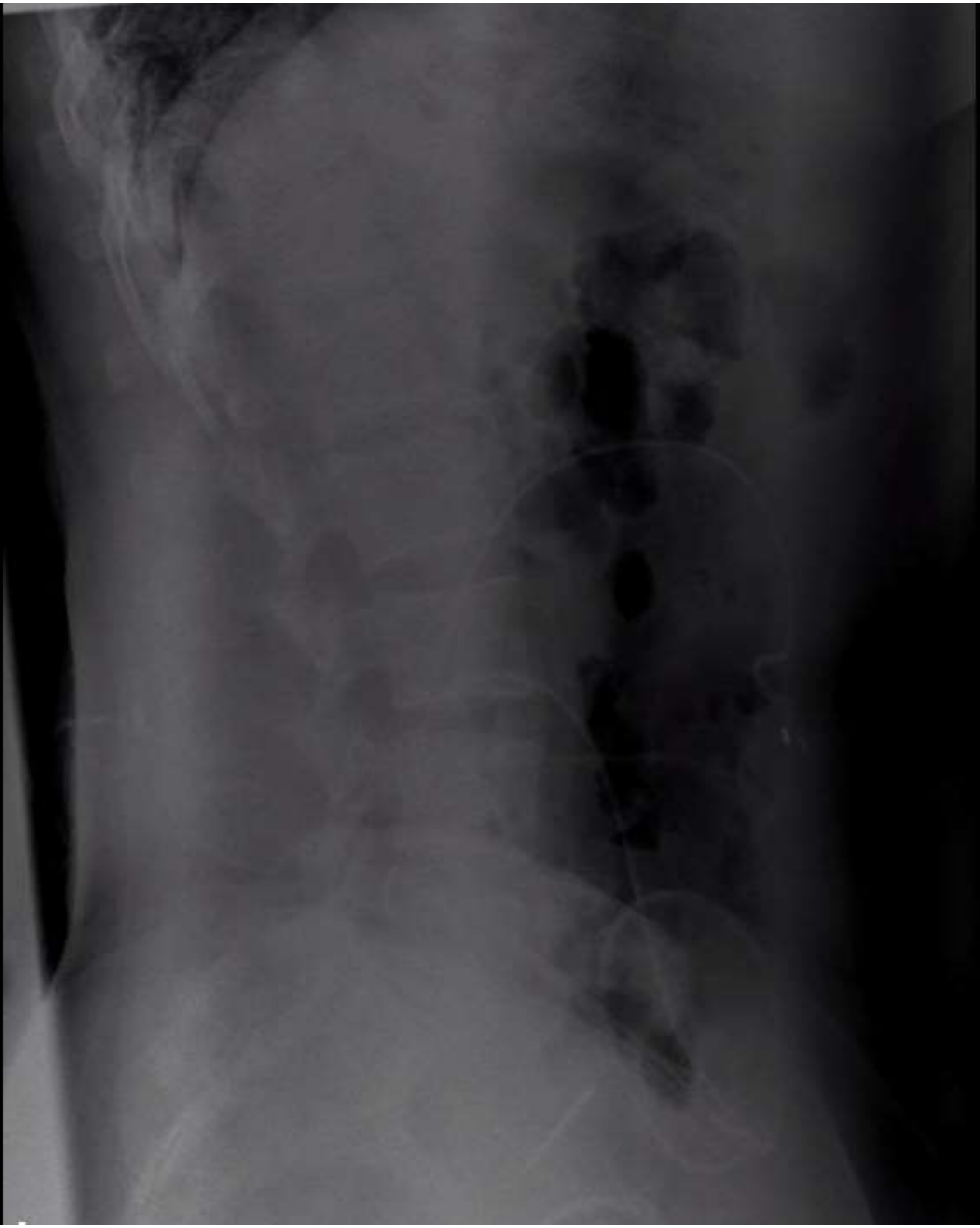

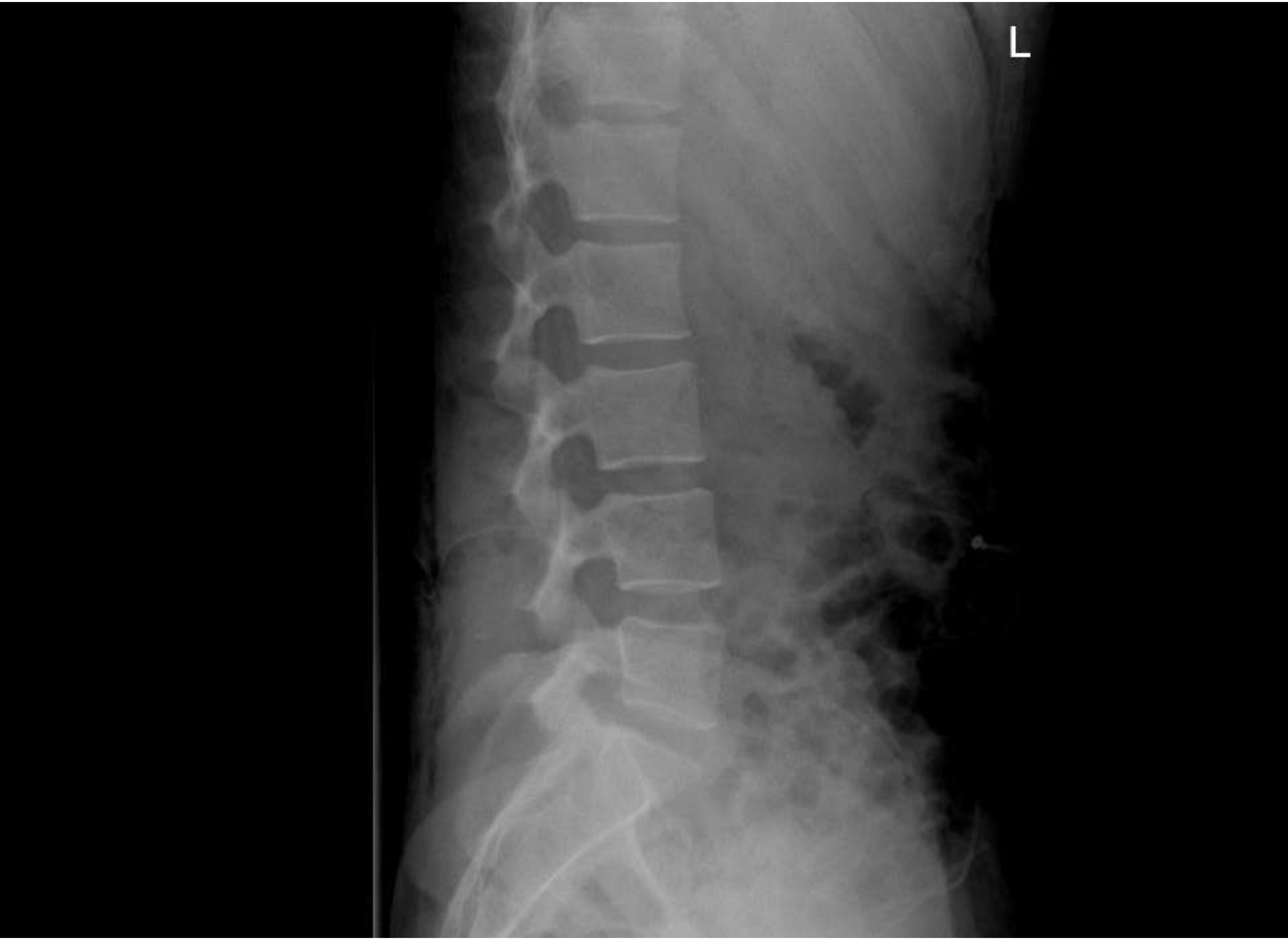
L

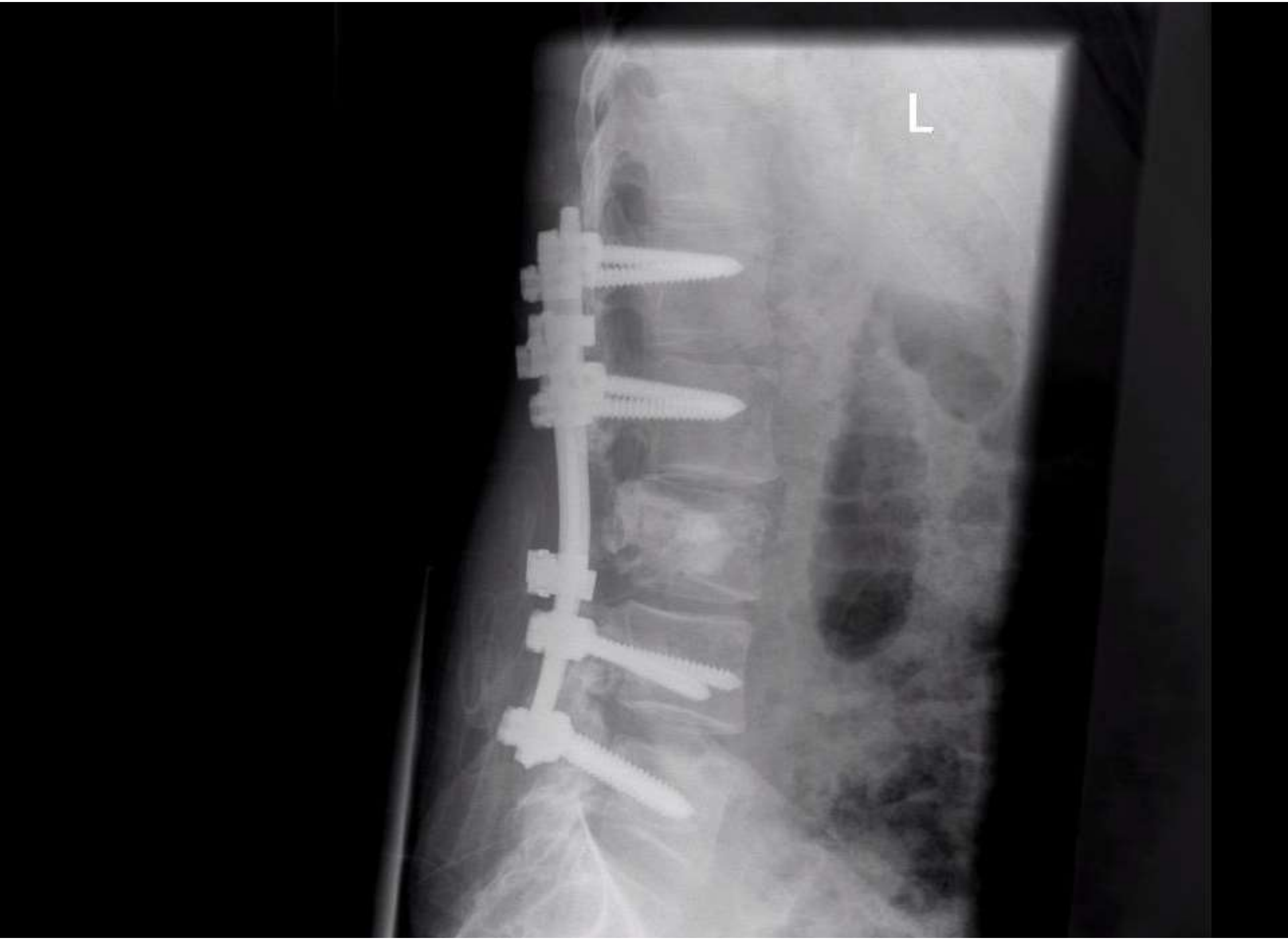
L

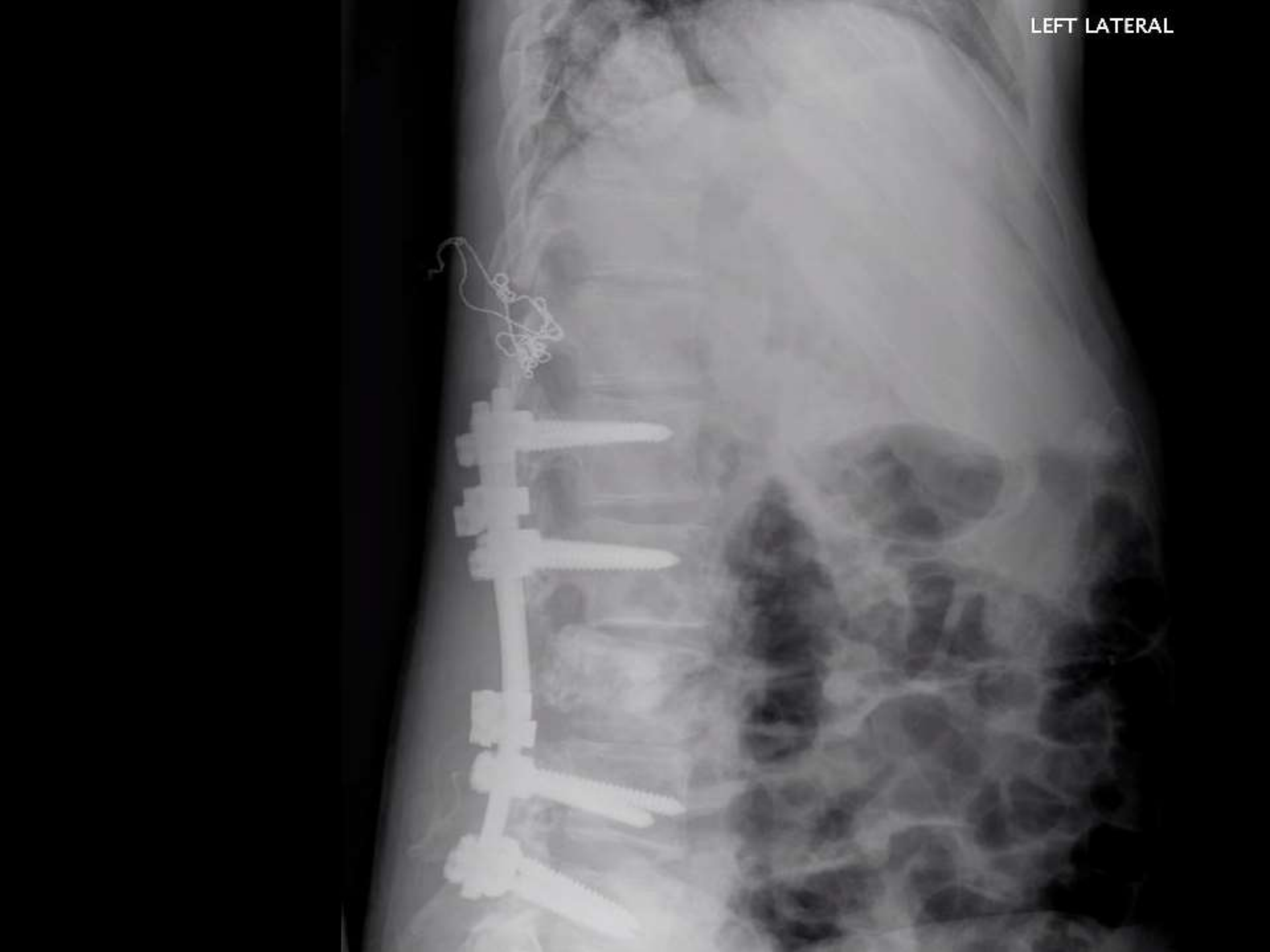
LEFT LATERAL

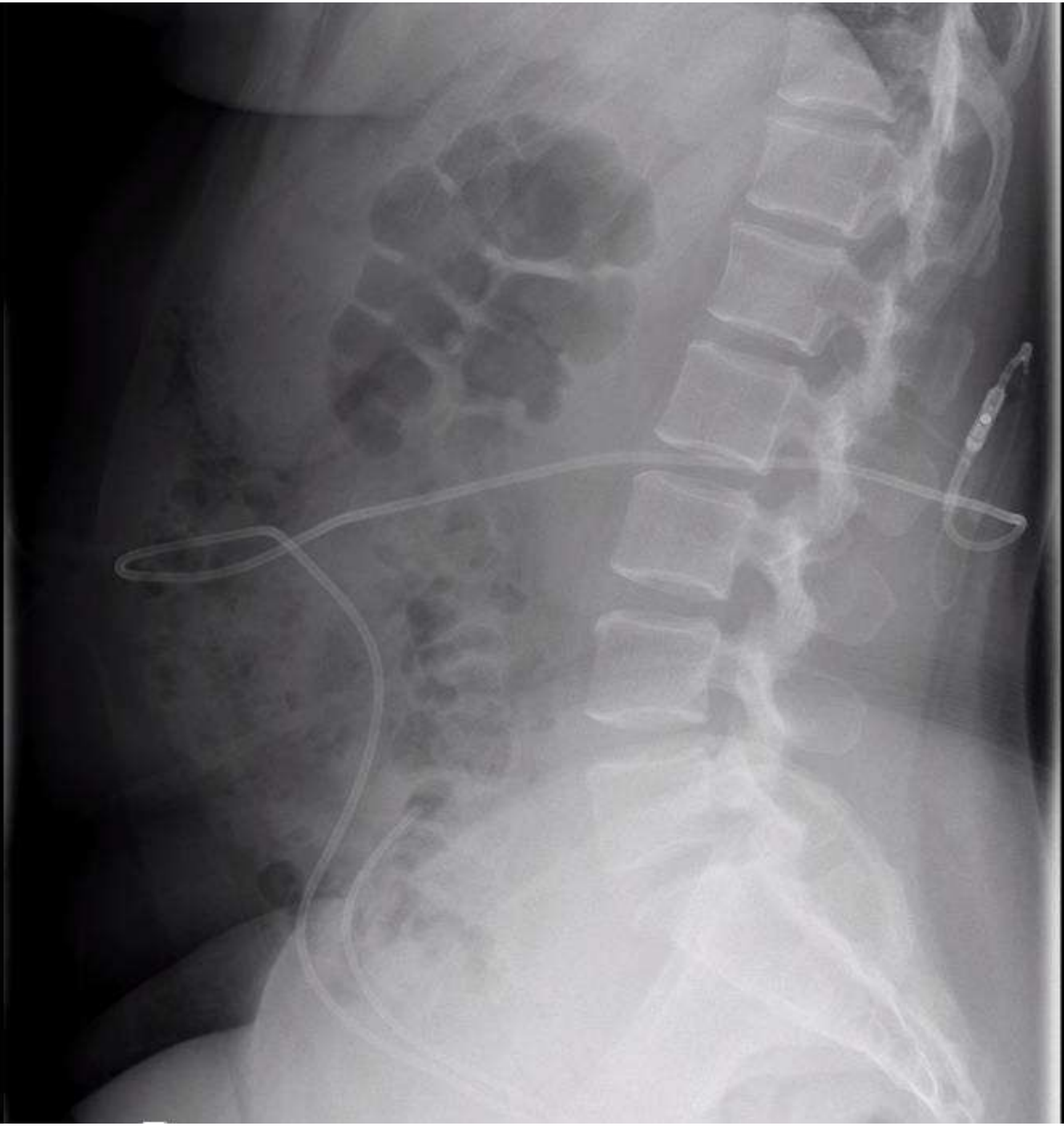

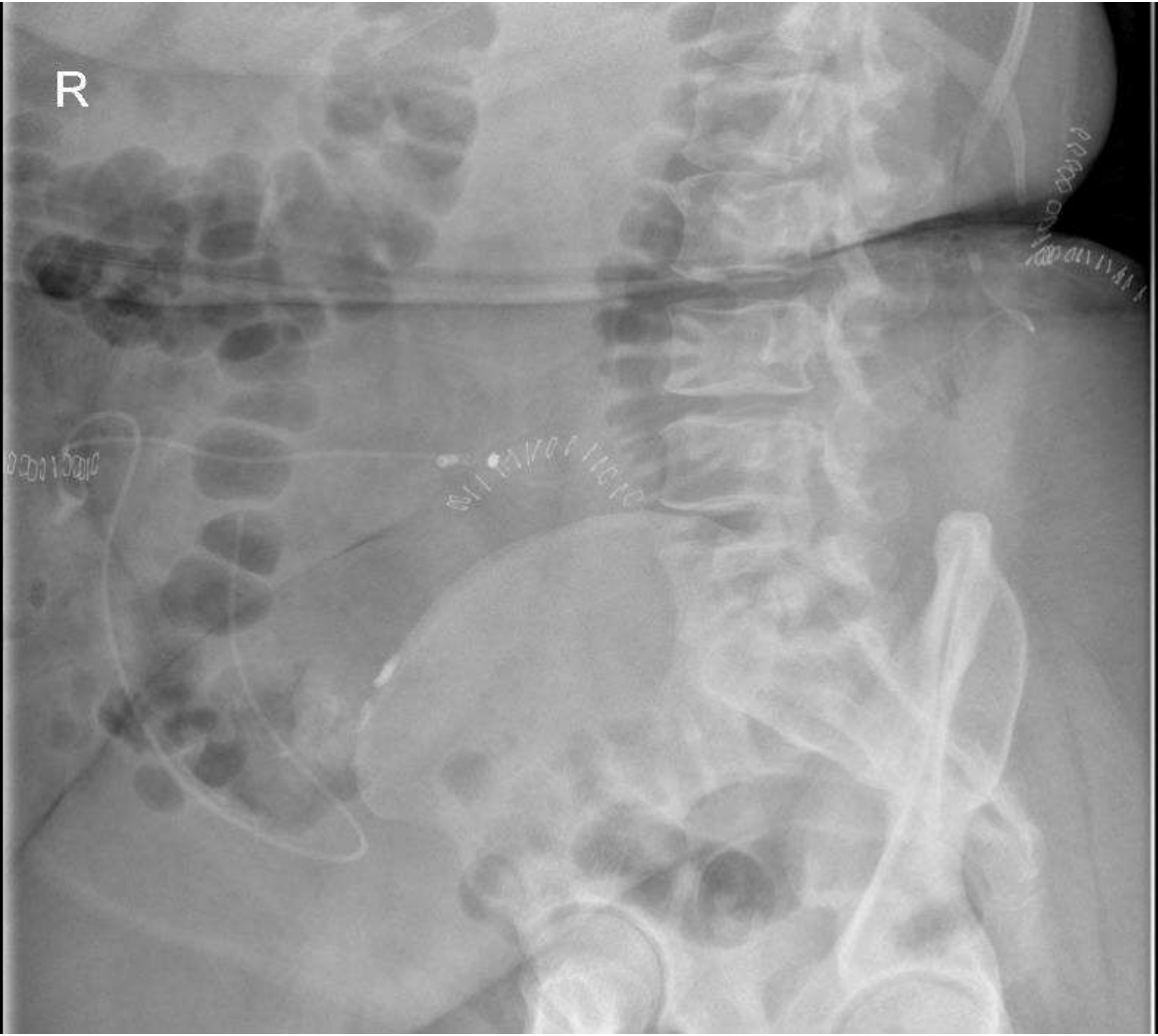
R

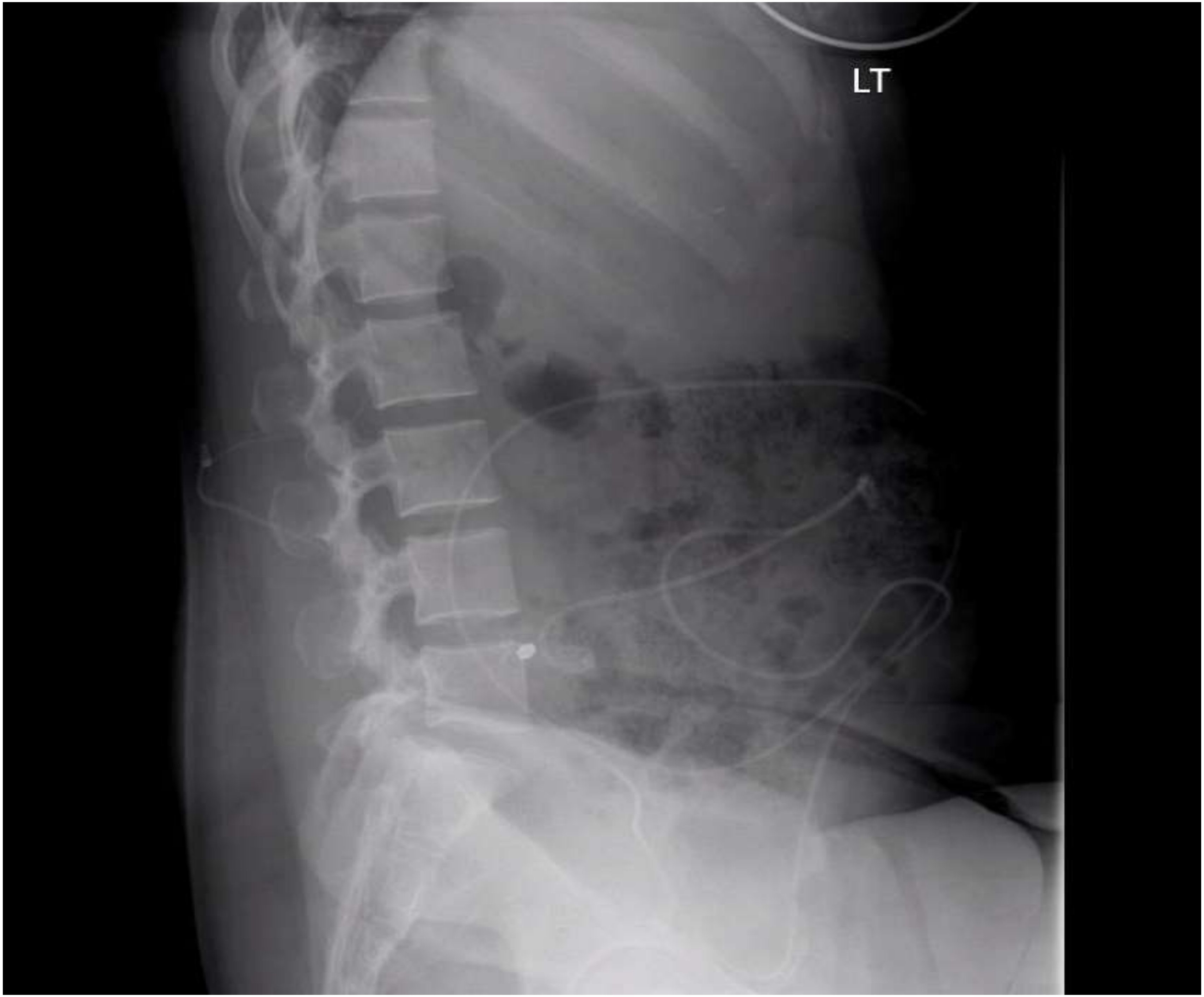
LT

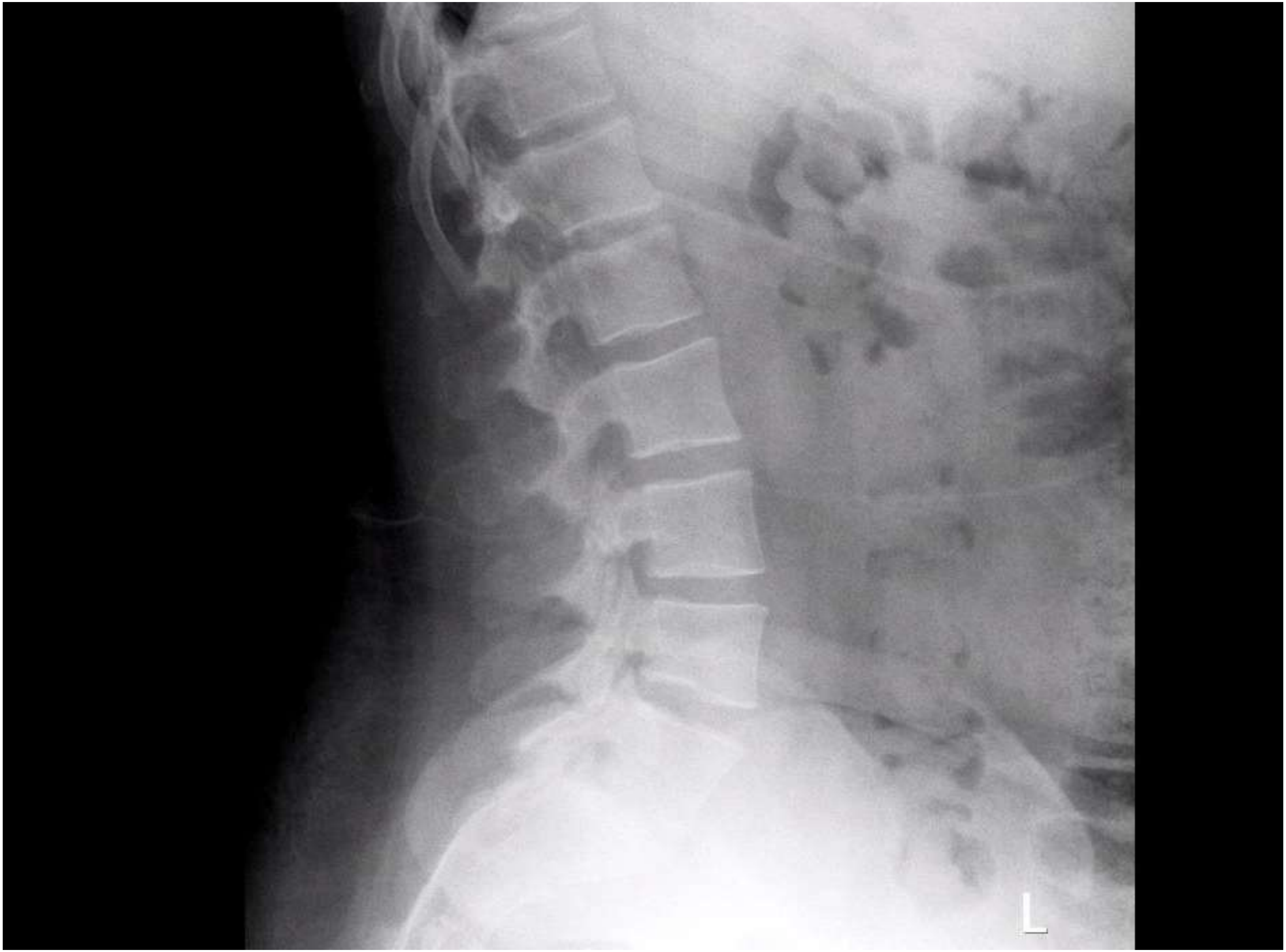
L

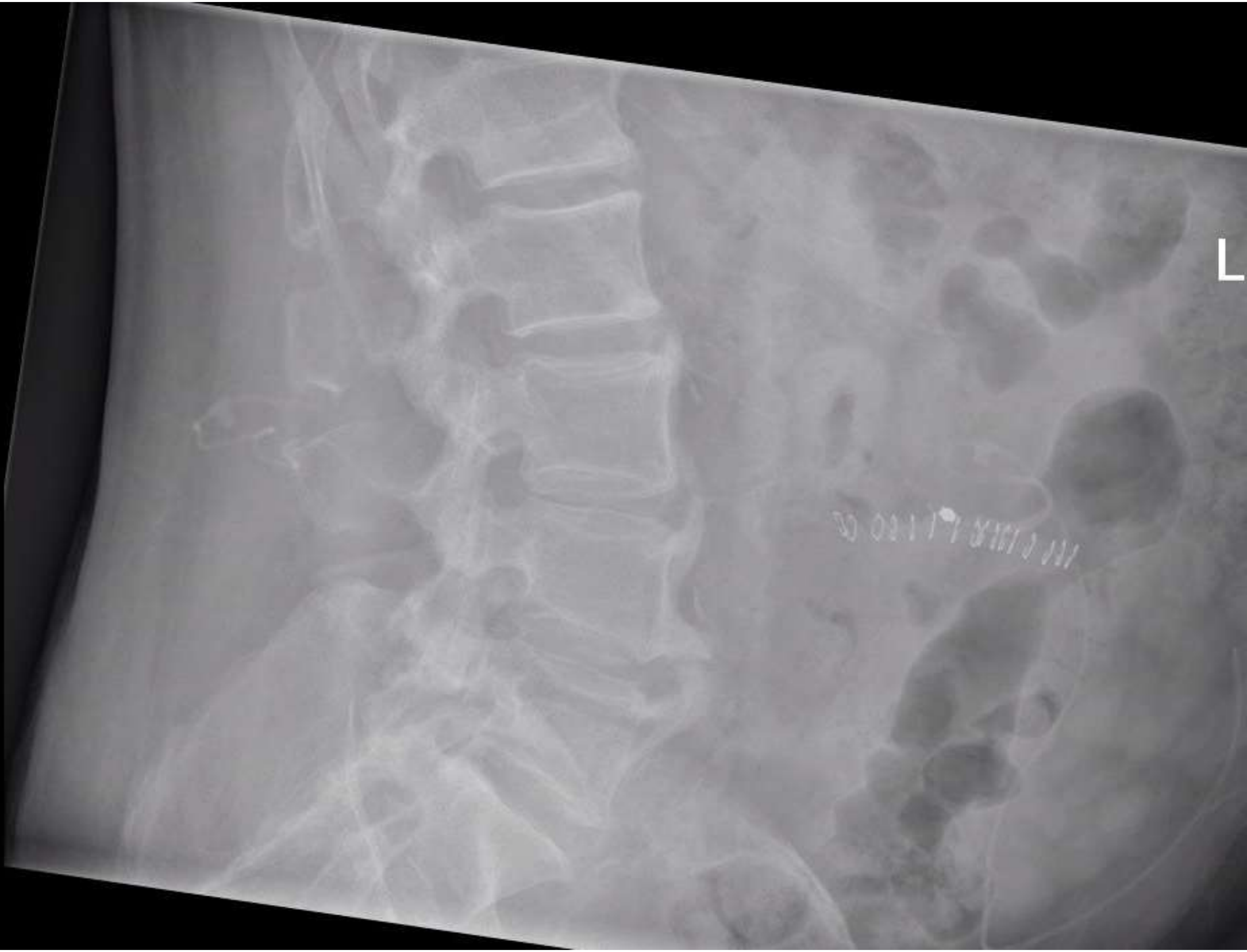
L

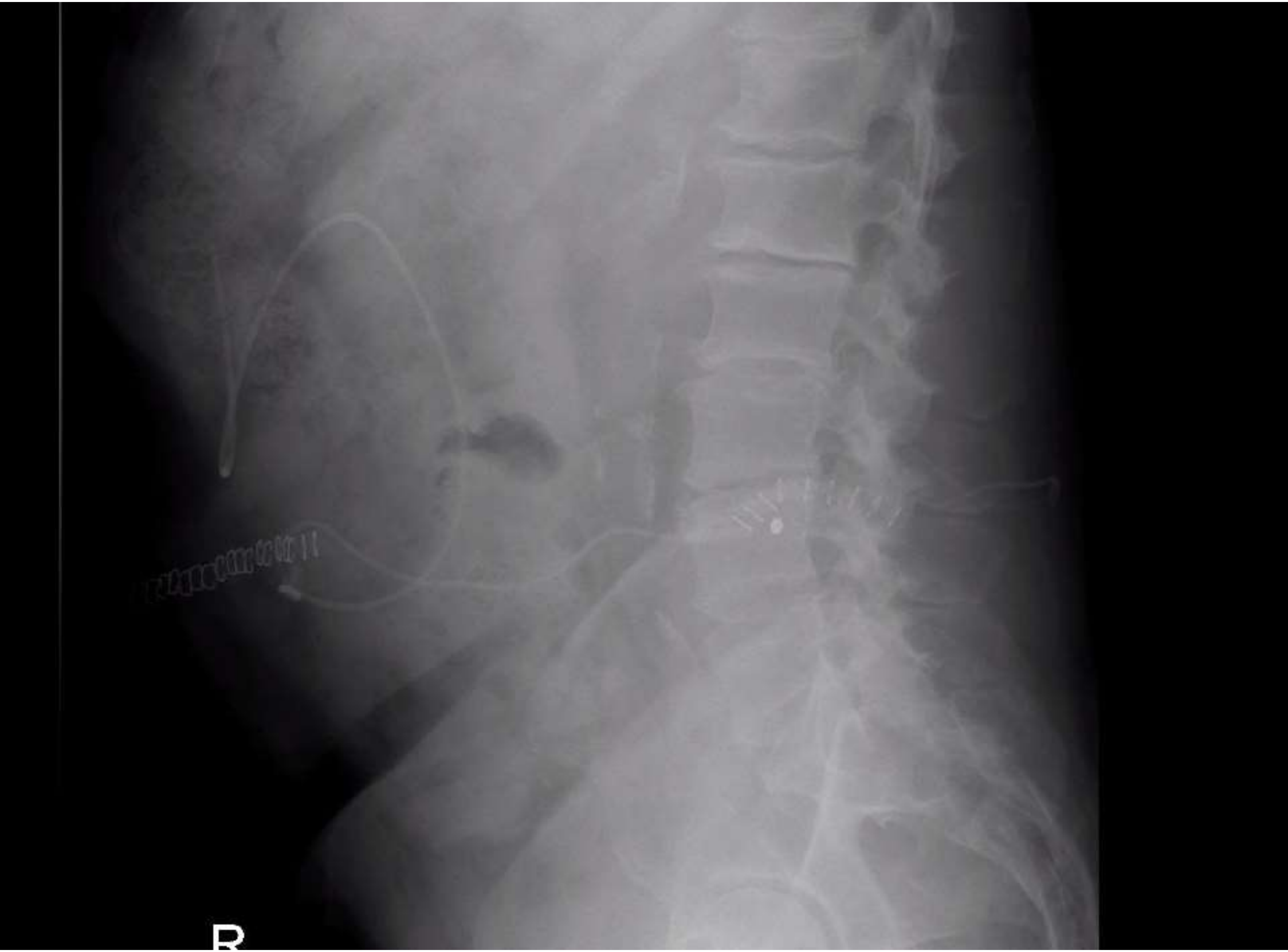
R

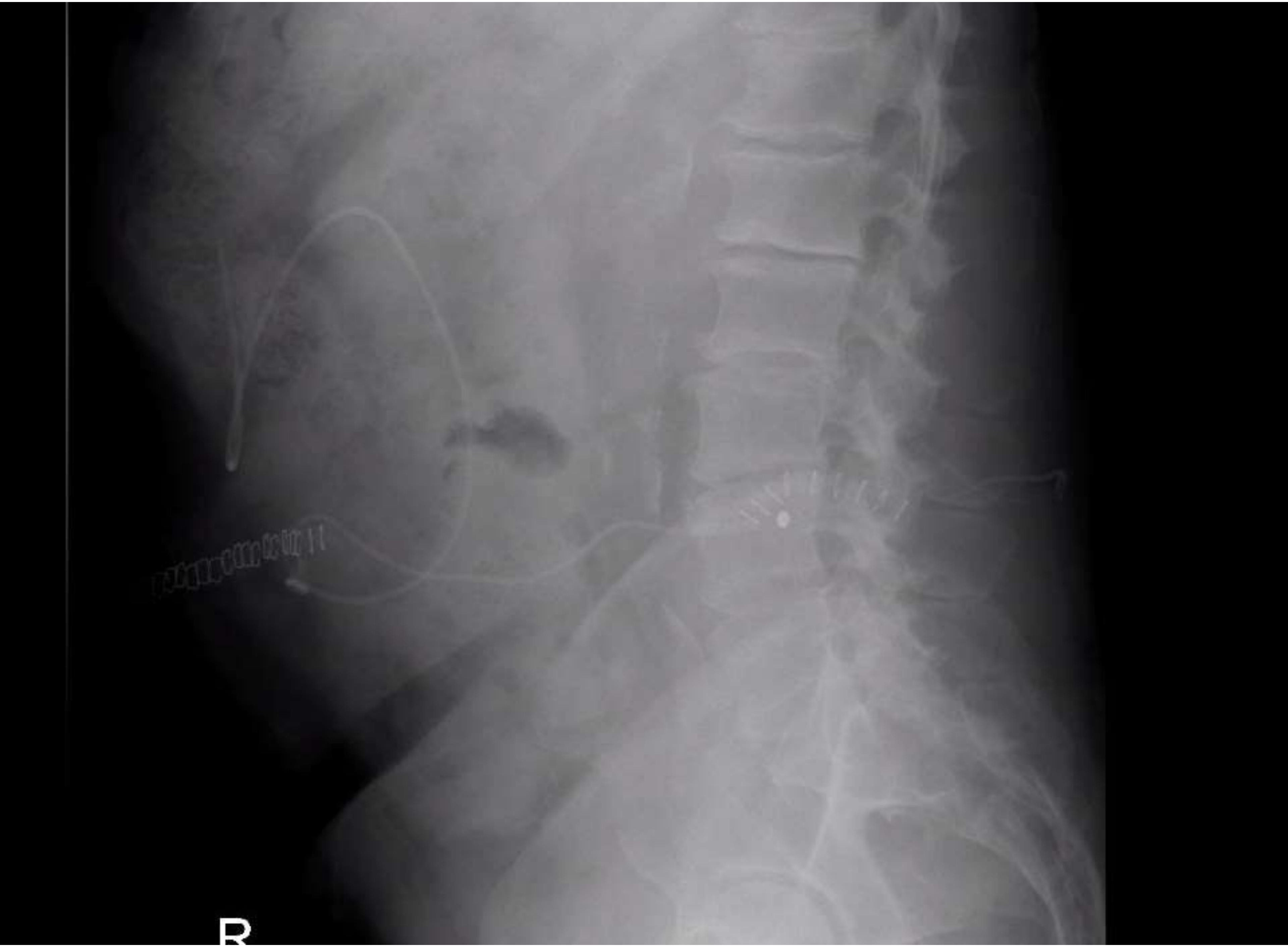
R

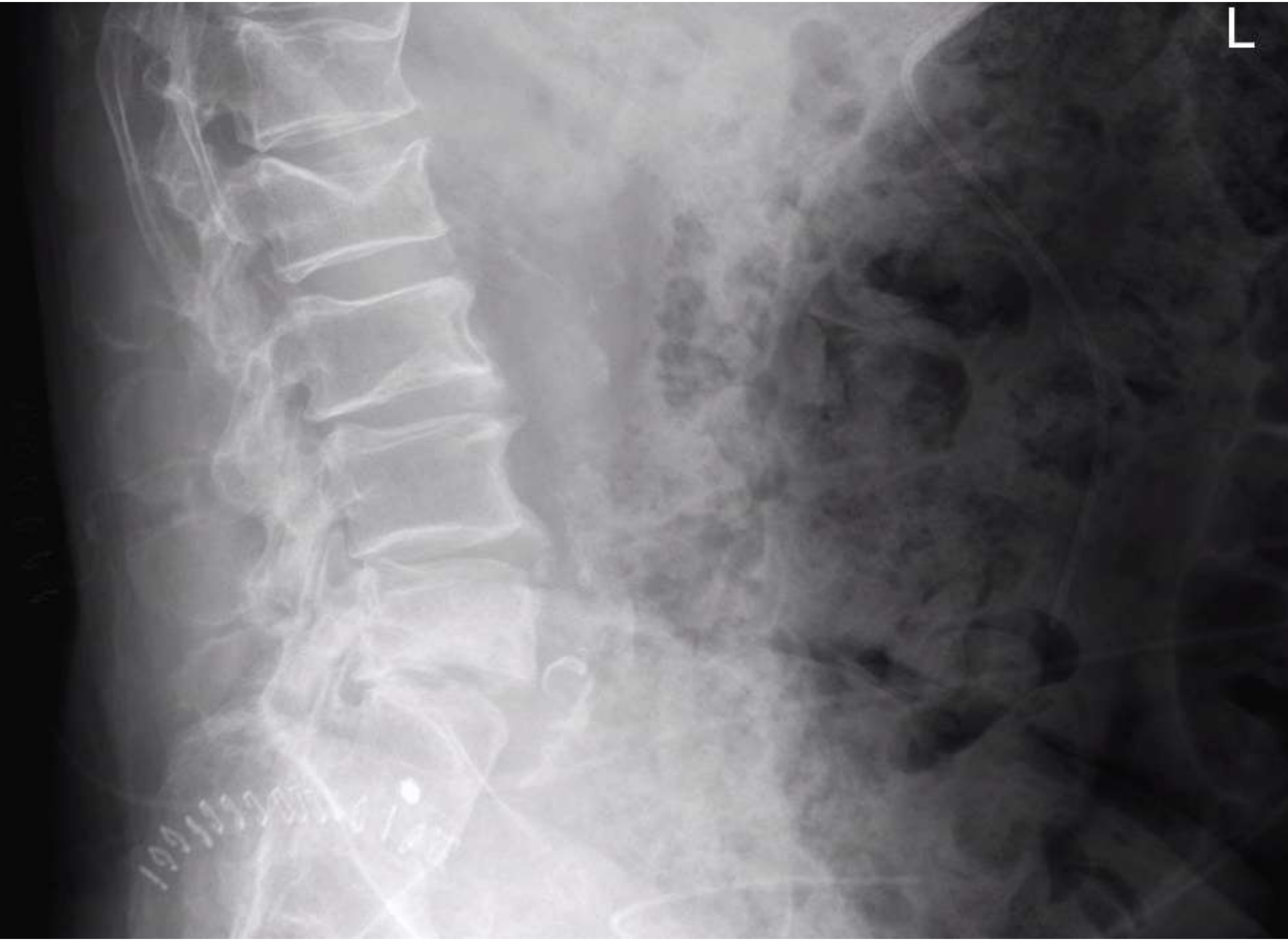
L

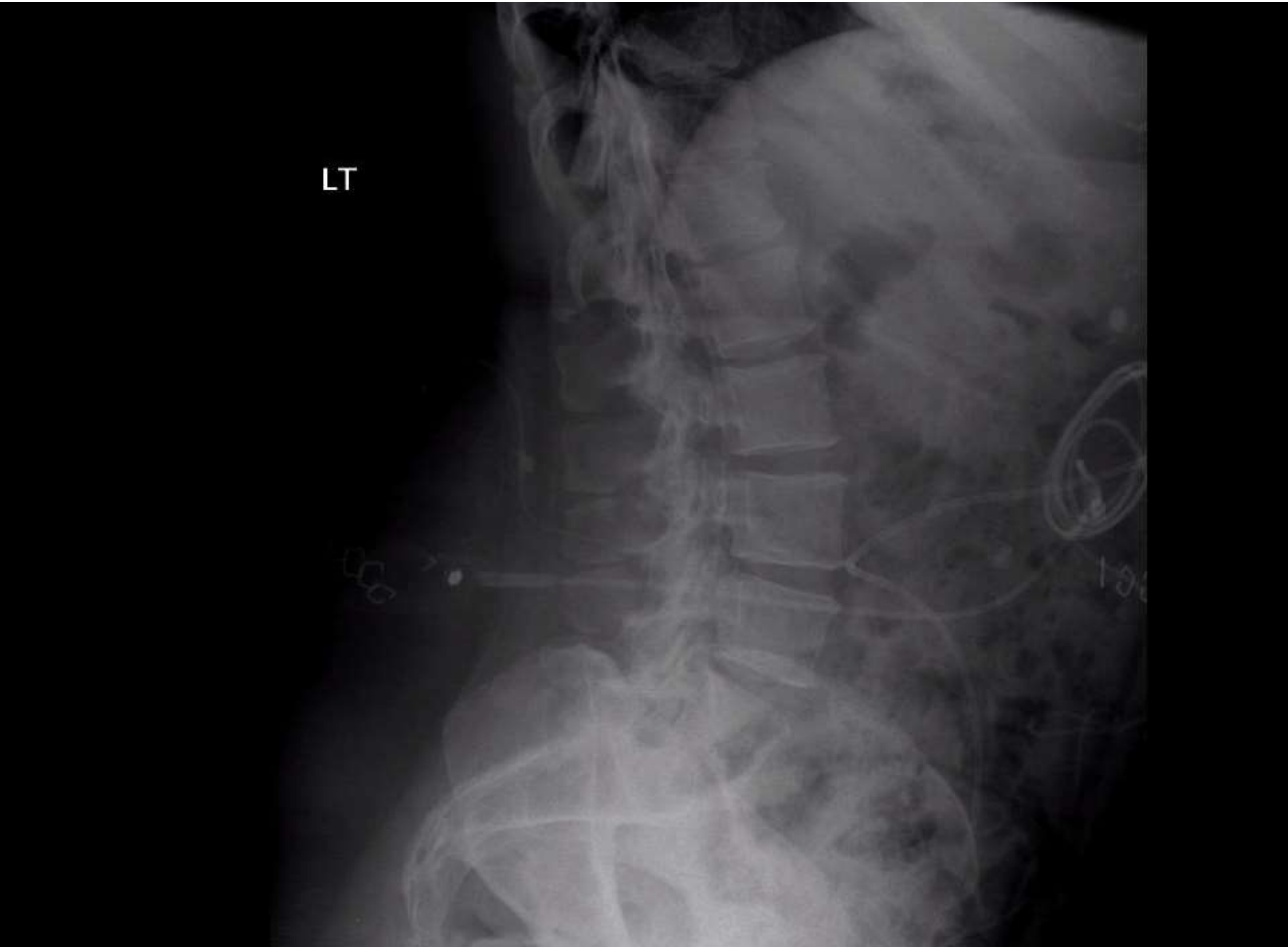
LT

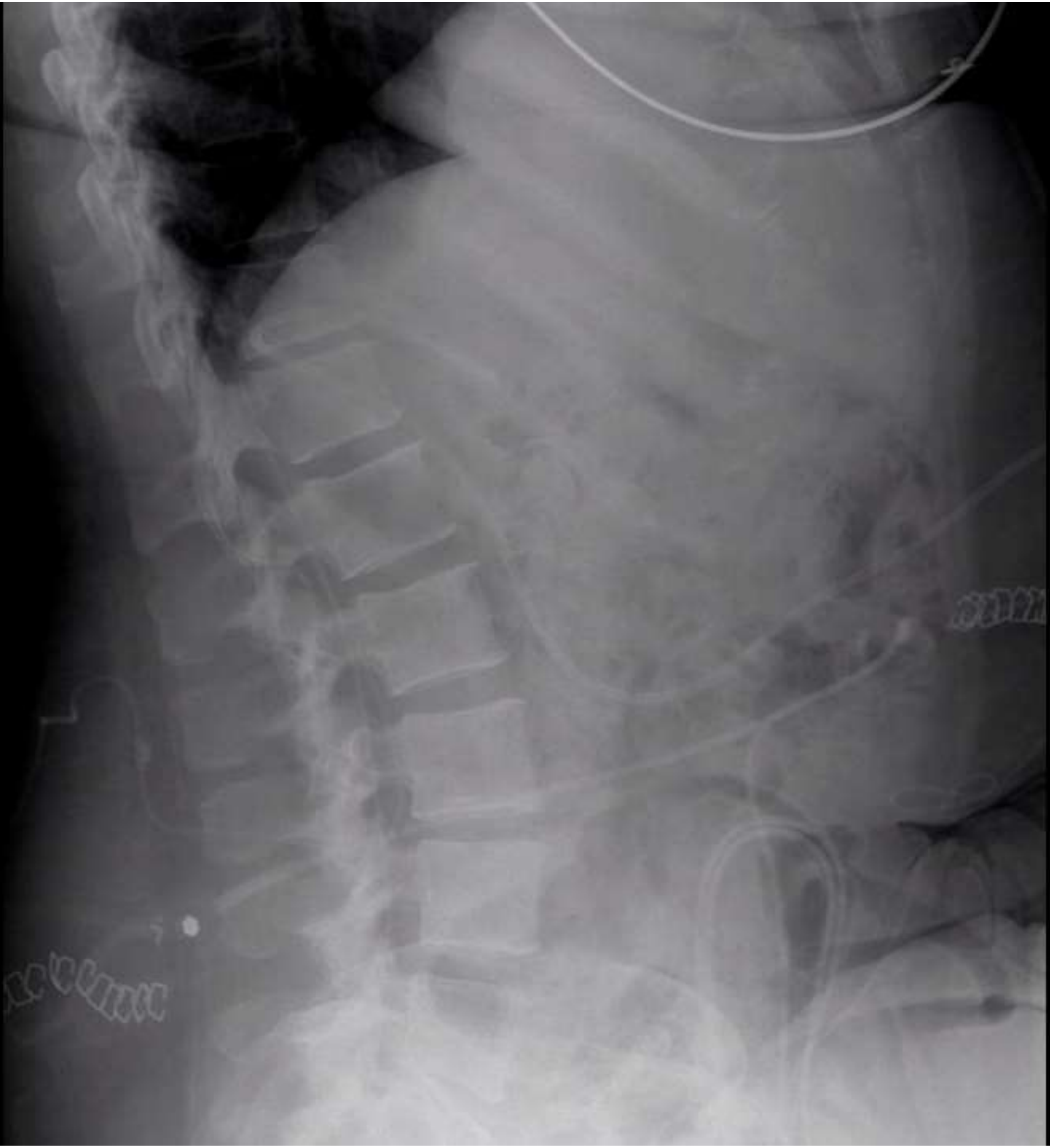

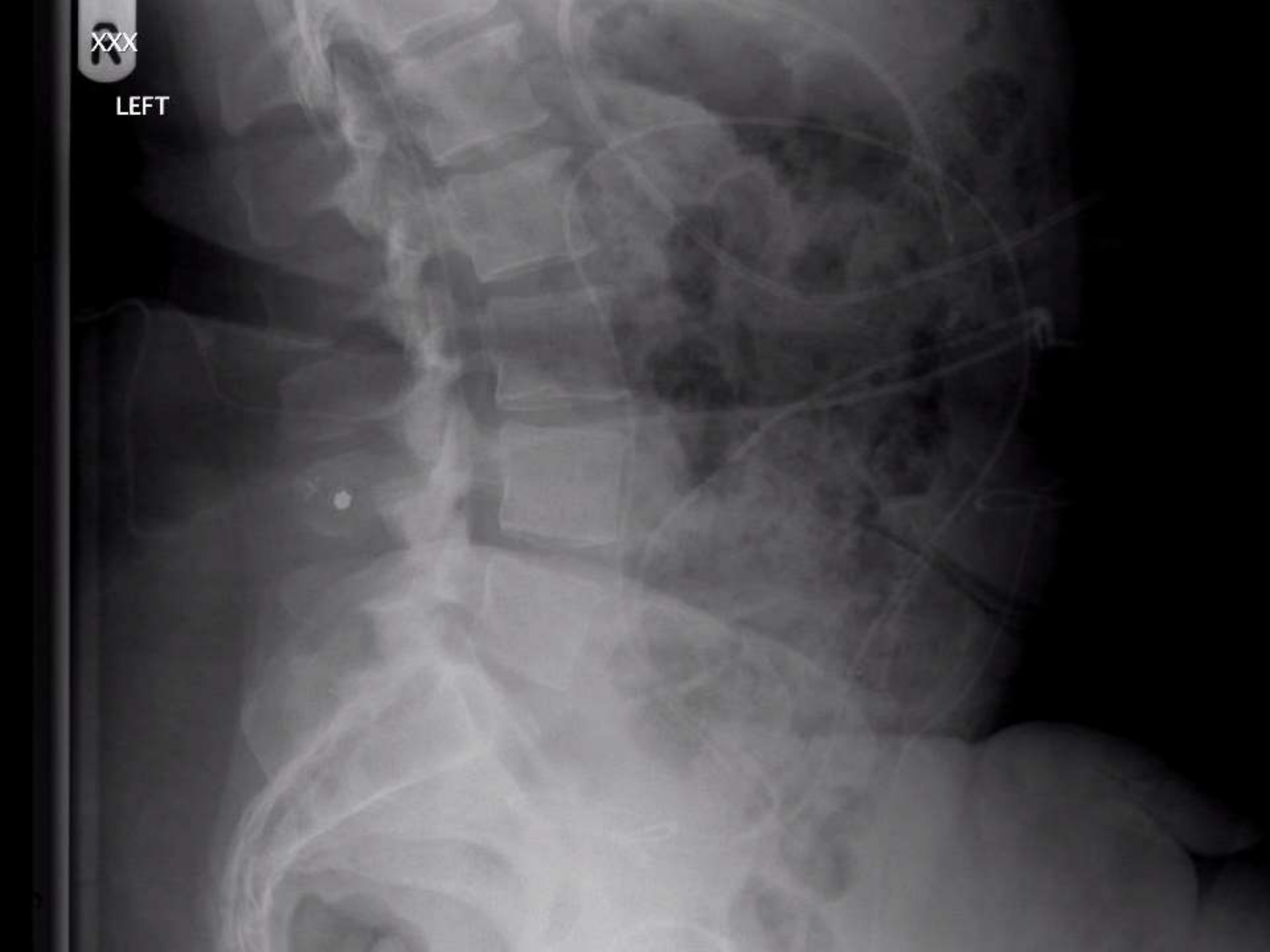
XXX
LEFT

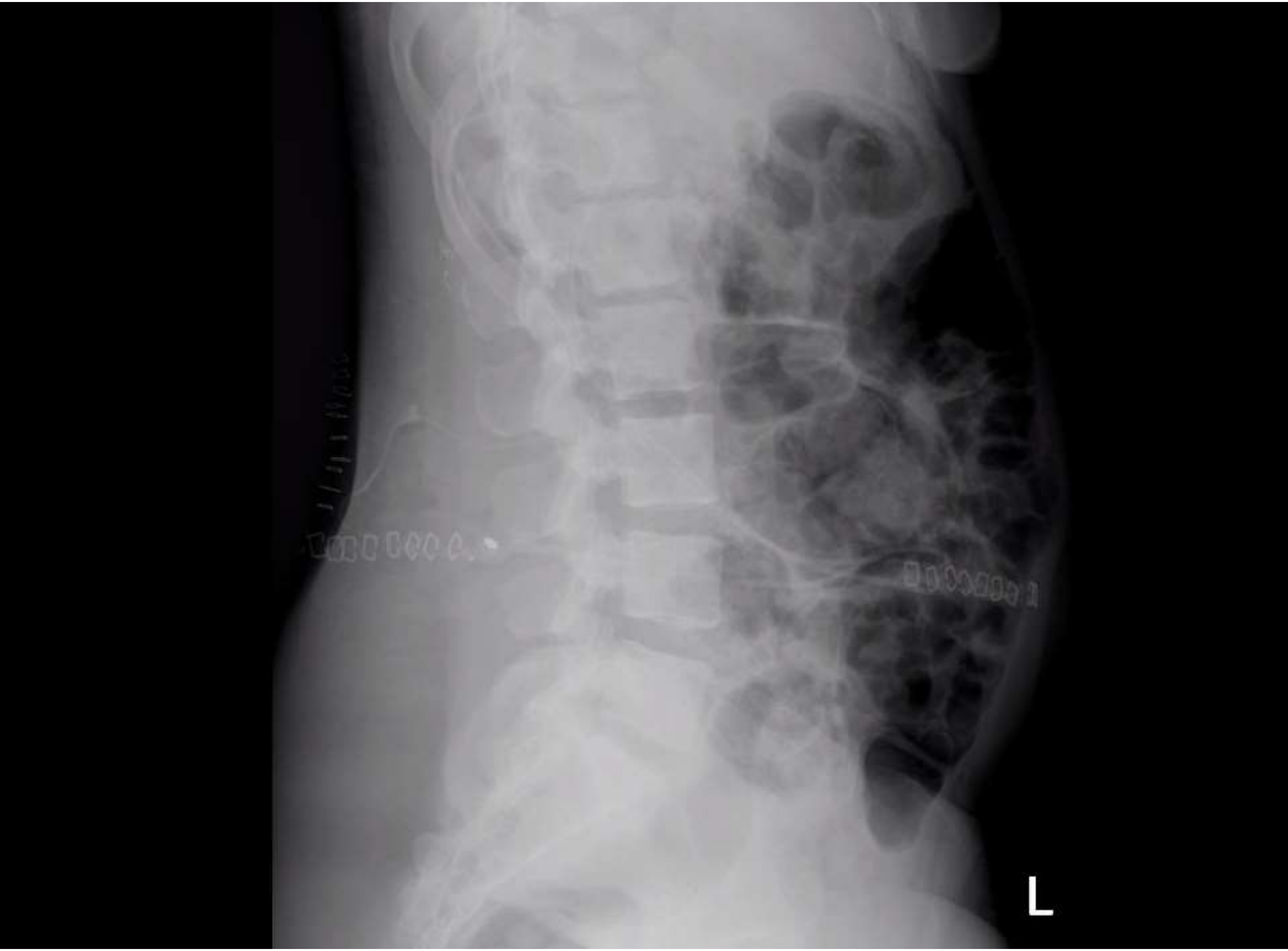
L

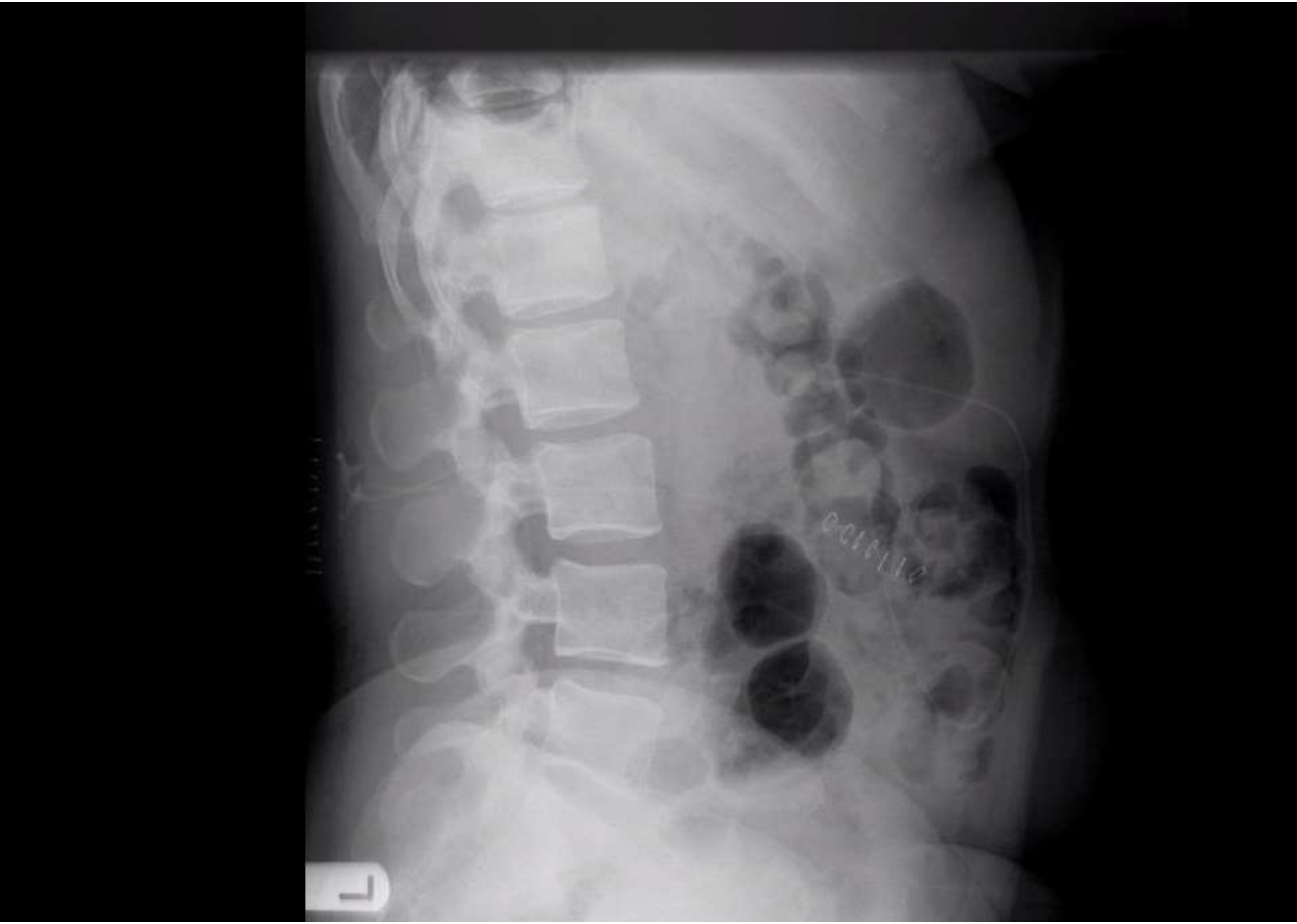
L

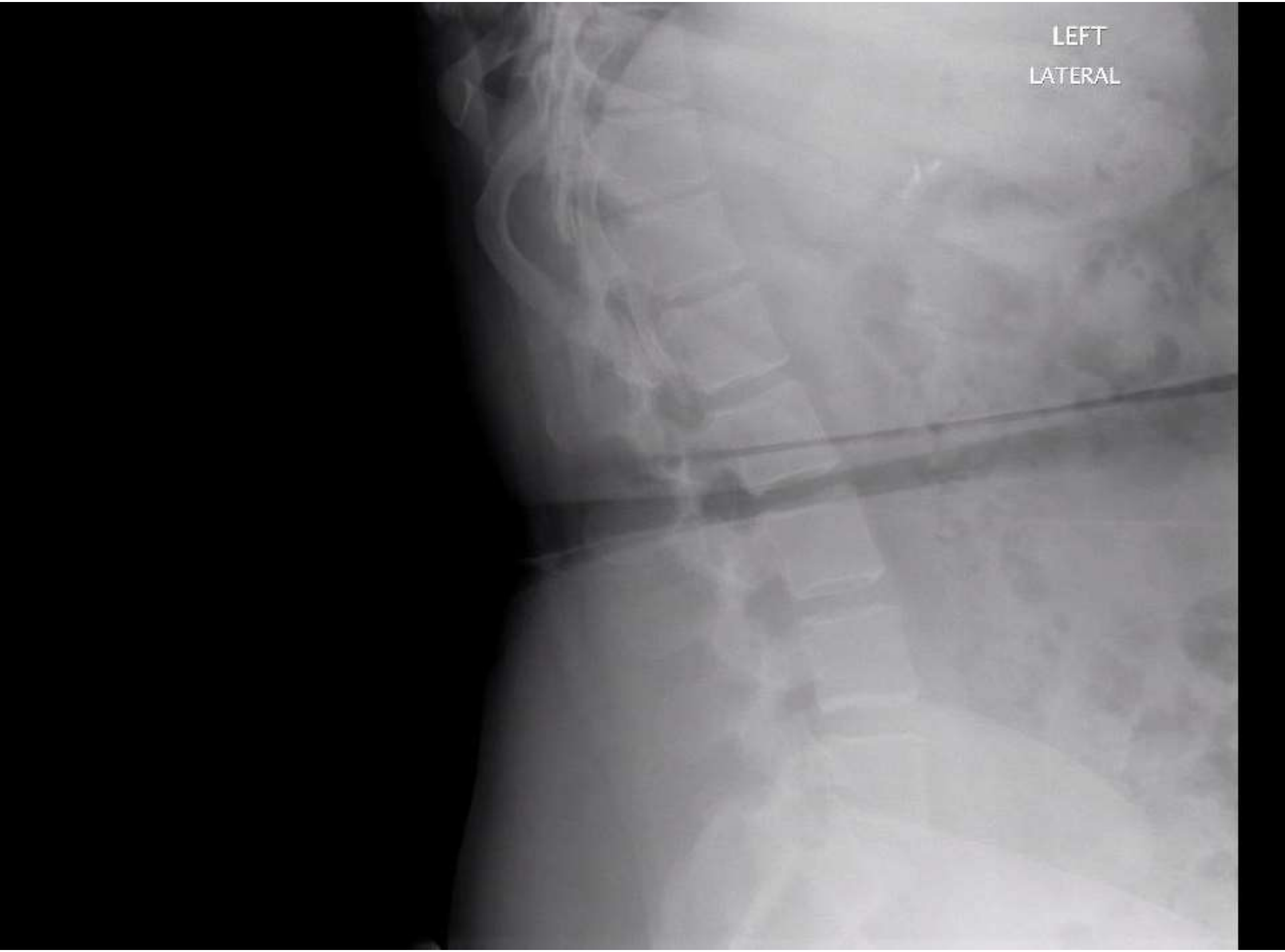
LEFT
LATERAL

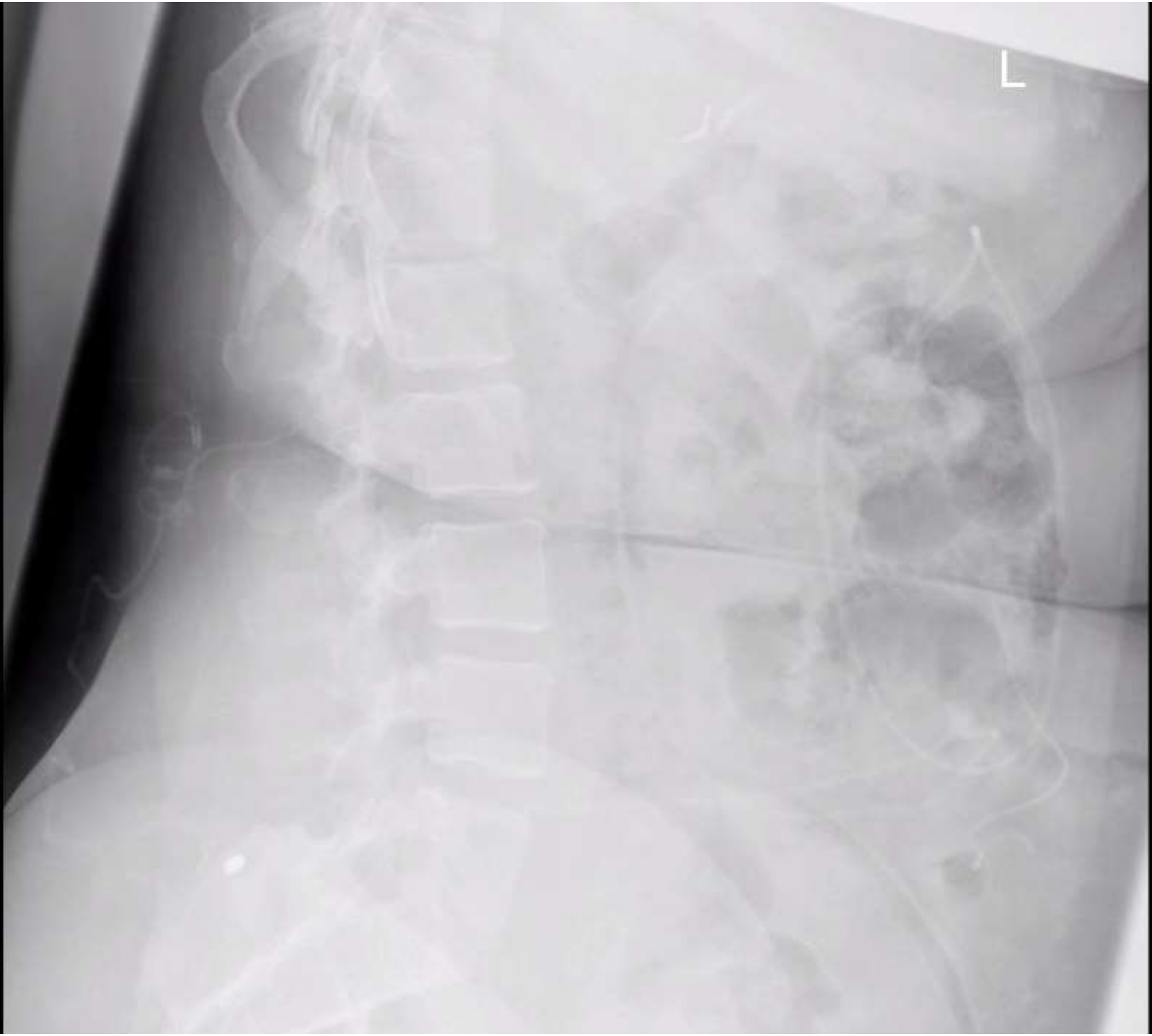
L

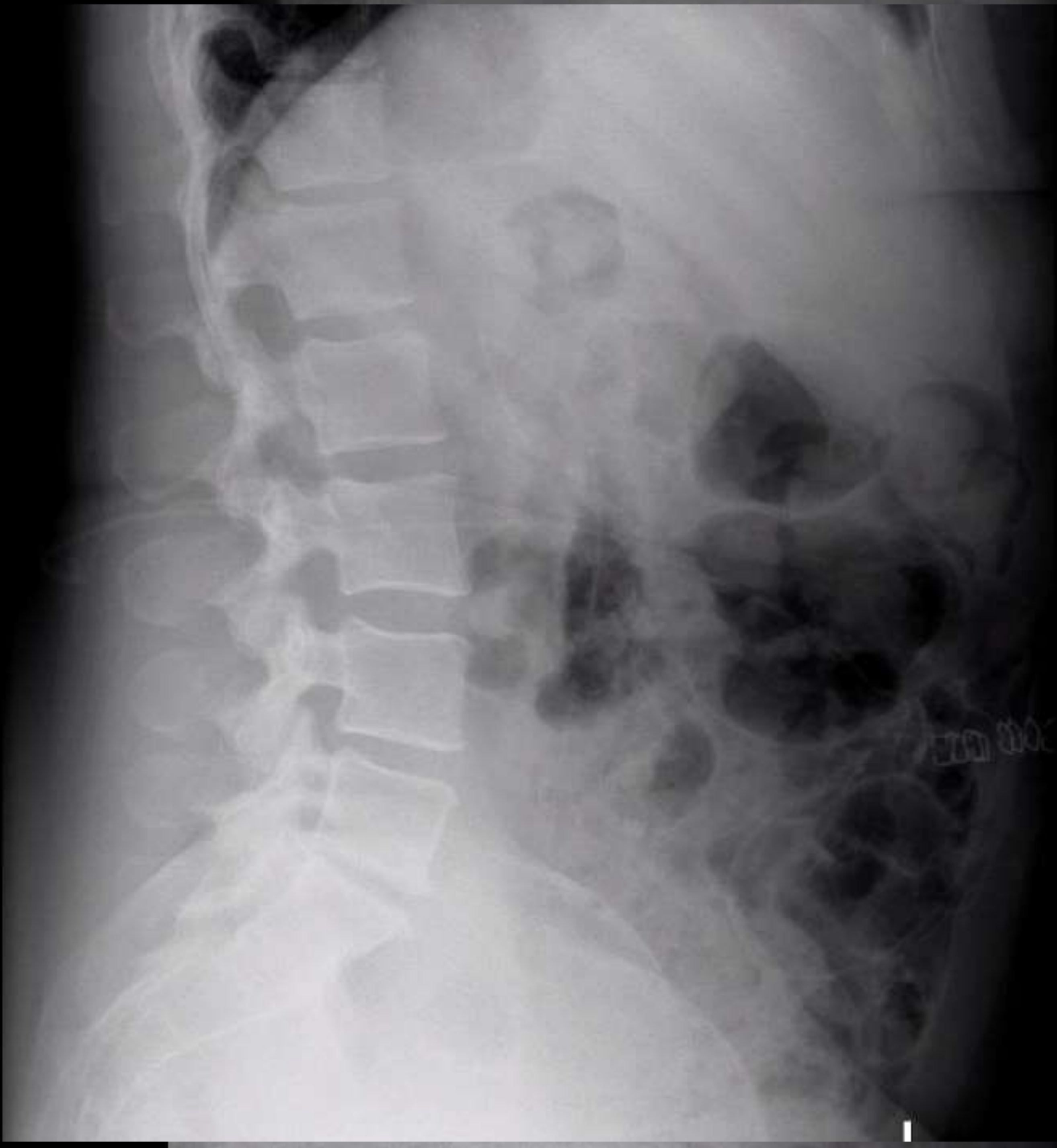

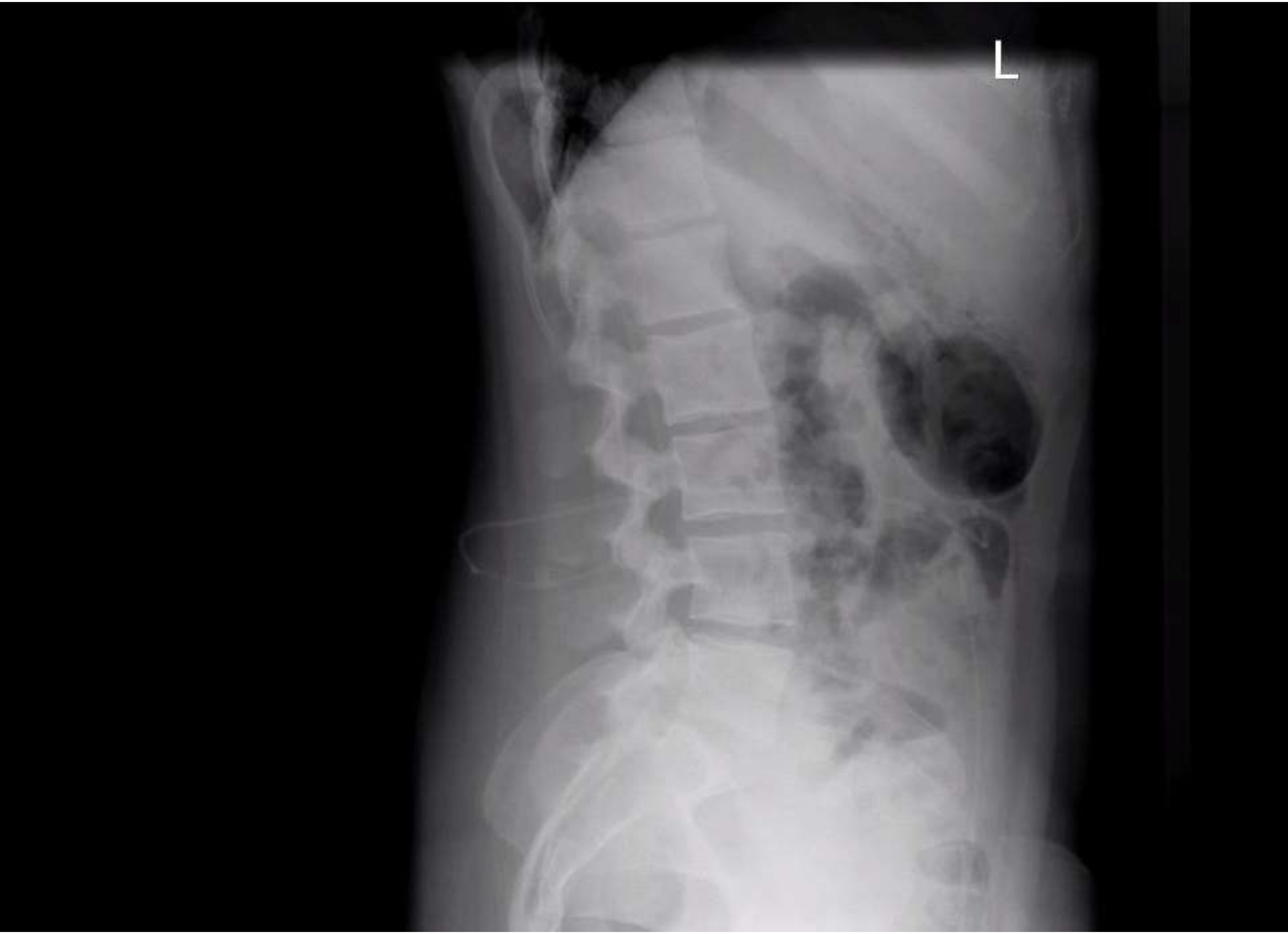
L

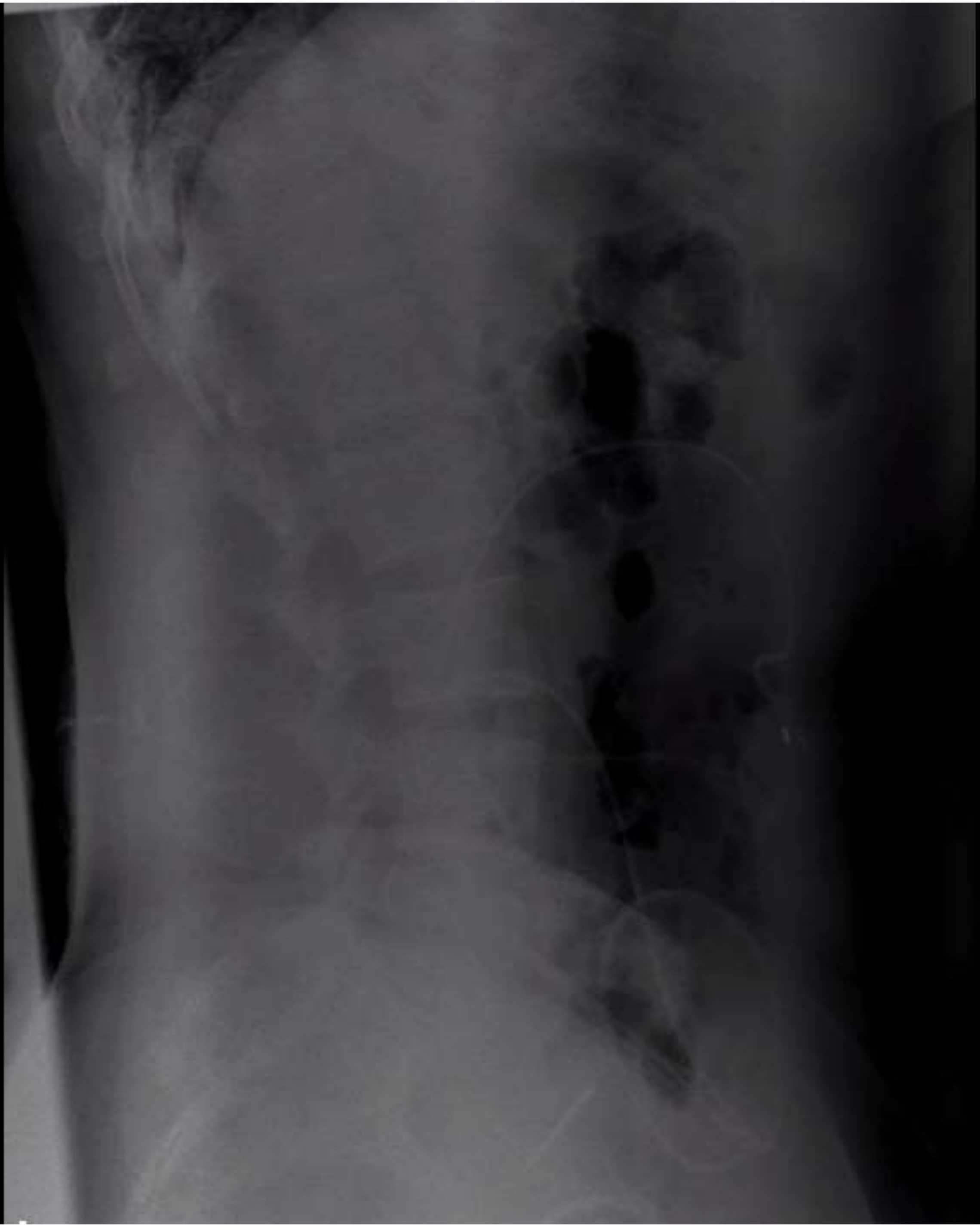

Supplement: X-rays showing the final position of the lumboperitoneal (LP) shunt in patients that underwent LP shunt insertion without fluoroscopic guidance [file f1000research-6-12972-s0000.tgz › 38188753-f5e4-49ee-a4b3-8682d9e92d57_Dataset_1.zip!/Dataset 1.pdf]
