# Supplementary material for: Lumboperitoneal shunt insertion without fluoroscopy guidance: Accuracy of placement in a series of 107 procedures
Source: F1000Res. 2017 Jun 30;6:565. Originally published 2017 Apr 25. [Version 2] doi: 10.12688/f1000research.11089.2 (PMC5506531; doi:10.12688/f1000research.11089.2)
Supplement: Peer review history

# Peer review: Referee response for version 2

**DOI:** 10.5256/f1000research.12972.r23922

**Published:** 2017-08-14

**Authors:** Rafid Al-mahfoudh

**Affiliations:** 1 Brighton and Sussex University Hospitals NHS Trust, Brighton, UK

I have read this submission. I believe that I have an appropriate level of expertise to confirm that it is of an acceptable scientific standard.

# Peer review: Referee response for version 2

**DOI:** 10.5256/f1000research.12972.r23923

**Published:** 2017-07-31

**Authors:** Rossana Romani

**Affiliations:** 1 Imperial College London, London, UK

The authors modified the article which is now ready for indexing.

I have read this submission. I believe that I have an appropriate level of expertise to confirm that it is of an acceptable scientific standard.

# Peer review: Referee response for version 2

**DOI:** 10.5256/f1000research.12972.r23924

**Published:** 2017-07-11

**Authors:** Uygur Er

**Affiliations:** 1 Neurosurgery Department, Medical School, Düzce University, Düzce, Turkey

I have read this submission. I believe that I have an appropriate level of expertise to confirm that it is of an acceptable scientific standard.

# Peer review: Referee response for version 1

**DOI:** 10.5256/f1000research.11961.r22200

**Published:** 2017-05-26

**Authors:** Rossana Romani

**Affiliations:** 1 Imperial College London, London, UK

The article is interesting and it can be indexed after revision. Suggestions for revision as follows:

1. The introduction is too long and it needs to be shortened. The majority of the concepts in the introduction should be in the paragraph of the discussion.
2. A further paragraph regarding the surgical techniques and the insertion of the lumbar catheter should be added in the Results section. Details of the surgical technique are in the Introduction section.
3. 17 patients had post-operative misplacement of the lumbar catheter with no symptoms nevertheless the authors conclude that intraoperative image guidance is suggested in all procedures to avoid misplacement of the lumbar catheter. The authors need to report the literature data regarding the misplacement of the lumbar catheter and clinical symptoms related.
4. I agree that further randomised clinical trial with a large number of patients can clarify the utility of intraoperative image guidance during lumboperitoneal shunt insertion. The conclusions need revision.

I have read this submission. I believe that I have an appropriate level of expertise to confirm that it is of an acceptable scientific standard, however I have significant reservations, as outlined above.

# Peer review: Referee response for version 1

**DOI:** 10.5256/f1000research.11961.r22202

**Published:** 2017-05-08

**Authors:** Rafid Al-mahfoudh

**Affiliations:** 1 Brighton and Sussex University Hospitals NHS Trust, Brighton, UK

The population studied needs to be accurately defined - do all the LP (Lumboperitoneal) shunt patients in this study have a diagnosis of IIH? 

 Have the authors used fluoroscopic guidance for LP shunts at their institution? This should be mentioned and if yes, then a comparison of proximal catheter placement could be made with and without fluoroscopy. 

 Some of the introduction would be best moved to the discussion (probably paragraph 5 onwards) as this mostly elaborates on possible causes which may explain the results of the study.

 Result section - clarify the indication for LP shunt (do all patients have a diagnosis of IIH)?

 The drive for quality in healthcare in general and a reduction in revision surgery specifically, continues to gain momentum worldwide. The authors discuss the possibility that intraoperative fluoroscopy can improve accurate LP shunt placement and should be praised for providing an honest appraisal of their results / misplaced proximal catheter rates.

I have read this submission. I believe that I have an appropriate level of expertise to confirm that it is of an acceptable scientific standard, however I have significant reservations, as outlined above.

# Peer review: Referee response for version 1

**DOI:** 10.5256/f1000research.11961.r22457

**Published:** 2017-05-03

**Authors:** Uygur Er

**Affiliations:** 1 Neurosurgery Department, Medical School, Düzce University, Düzce, Turkey

Authors claimed that using a fluoroscopic system may reduce the risk of misplacement of the proximal catheter. However, they did not see any spinal cord injuries or other complications due to misplacement. In conclusion section this needs clarifying. Misplacement rate was given but this was emphasized as a technical complication, not a harmful cause. 

 The second vital point is that it is unclear the meaning of misplacement according to this article. It should be clarified “misplacement”. Did they use this term as the inserting vertebral level or the end position of the proximal catheter? Figure 1 showed wrong insertion points, but in the introduction section this was defined as “misplaced inside thecal sac due to migration”. Besides, position of the patient doesn’t change vertebral levels due to anatomical landmarks. For example, in lateral position, superior iliac line passes L-4/5 level like in anatomical position. Authors should give some strictly defined methods using fluoroscopy, positioning and evaluation to allow replication by others.

 Following clarifying these points, this article may accepted for indexing after reviewing of the revised version.

I have read this submission. I believe that I have an appropriate level of expertise to confirm that it is of an acceptable scientific standard, however I have significant reservations, as outlined above.
